# Predicting HLA genotypes using unphased and flanking single-nucleotide polymorphisms in Han Chinese population

**DOI:** 10.1186/1471-2164-15-81

**Published:** 2014-01-29

**Authors:** Ai-Ru Hsieh, Su-Wei Chang, Pei-Lung Chen, Chen-Chung Chu, Ching-Lin Hsiao, Wei-Shiung Yang, Chien-Ching Chang, Jer-Yuarn Wu, Yuan-Tsong Chen, Tien-Chun Chang, Cathy SJ Fann

**Affiliations:** 1 Institute of Biomedical Sciences, Academia Sinica, Nankang, Taipei, Taiwan; 2 Clinical Informatics and Medical Statistics Research Center, Chang Gung University College of Medicine, Taoyuan, Taiwan; 3 Department of Medical Genetics, National Taiwan University Hospital, Taipei, Taiwan; 4 Department of Internal Medicine, National Taiwan University Hospital, Taipei, Taiwan; 5 Graduate Institute of Clinical Medicine, College of Medicine, National Taiwan University, Taipei, Taiwan; 6 Department of Internal Medicine, College of Medicine, National Taiwan University, Taipei, Taiwan; 7 National Genotyping Center, Academia Sinica, Taipei, Taiwan; 8 Graduate Institute of Chinese Medical Science, China Medical University, Taichung, Taiwan; 9 Department of Pediatrics, Duke University Medical Center, Durham, North Carolina, USA; 10 Immunogenetics laboratory, Medical Research Department, Mackay Memorial Hospital, Taipei, Taiwan; 11 Research Center for Developmental Biology and Regenerative Medicine, National Taiwan University, Taipei, Taiwan; 12 Graduate Institute of Medical Genomics and Proteomics, National Taiwan University College of Medicine, Taipei, Taiwan; 13 Graduate Institute of Biostatistics, China Medical University, Taichung, Taiwan

**Keywords:** Major histocompatibility complex (MHC), *Human leukocyte antigen* (*HLA*), Single-nucleotide polymorphisms (SNPs)

## Abstract

**Background:**

Genetic variation associated with human leukocyte antigen (*HLA*) genes has immunological functions and is associated with autoimmune diseases. To date, large-scale studies involving classical *HLA* genes have been limited by time-consuming and expensive *HLA*-typing technologies. To reduce these costs, single-nucleotide polymorphisms (SNPs) have been used to predict *HLA*-allele types. Although *HLA* allelic distributions differ among populations, most prediction model of *HLA* genes are based on Caucasian samples, with few reported studies involving non-Caucasians.

**Results:**

Our sample consisted of 437 Han Chinese with Affymetrix 5.0 and Illumina 550 K SNPs, of whom 214 also had data on Affymetrix 6.0 SNPs. All individuals had *HLA* typings at a 4-digit resolution. Using these data, we have built prediction model of *HLA* genes that are specific for a Han Chinese population. To optimize our prediction model of HLA genes, we analyzed a number of critical parameters, including flanking-region size, genotyping platform, and imputation. Predictive accuracies generally increased both with sample size and SNP density.

**Conclusions:**

SNP data from the HapMap Project are about five times more dense than commercially available genotype chip data. Using chips to genotype our samples, however, only reduced the accuracy of our *HLA* predictions by only ~3%, while saving a great deal of time and expense. We demonstrated that classical *HLA* alleles can be predicted from SNP genotype data with a high level of accuracy (80.37% (*HLA-B*) ~95.79% (*HLA-DQB1*)) in a Han Chinese population. This finding offers new opportunities for researchers in obtaining *HLA* genotypes via prediction using their already existing chip datasets. Since the genetic variation structure (e.g. SNP, *HLA*, Linkage disequilibrium) is different between Han Chinese and Caucasians, and has strong impact in building prediction models for *HLA* genes, our findings emphasize the importance of building ethnic-specific models when analyzing human populations.

## Background

With the advent of high-throughput genotyping technologies, it is now relatively easy to obtain large-scale, genome-wide data concerning single-nucleotide polymorphisms (SNPs) in humans. This allows for more thorough analyses of questions that involve population genetics. Multiple SNPs often cover or flank functionally important genes, such as *human leukocyte antigen* (*HLA*) genes that comprise the major histocompatibility complex (MHC) in humans. The *HLA*s localize to chromosome 6p and include MHC class I (*HLA-A*, *HLA-B*, and *HLA–C*) and MHC class II (*HLA-DR*, *HLA-DQ* and *HLA-DP*) genes. Unlike bi-allelic SNPs, *HLA* genes are extremely polymorphic. Currently, more than 8794 alleles for the *HLA* loci have been identified in various populations, including 2862 *HLA-B* alleles according to the IMGT/HLA database 3.11.0 version [1]. Mismatched *HLA* alleles can lead to graft rejection and graft-versus-host diseases [1,2]. *HLA* genes also play critical roles in both population genetics and immune-related disease status [3,4]. Furthermore, previous comparative studies have shown that immune systems are generally under strong selective pressures, which are likely driven by virus-host interactions [5,6]. Because of these selective pressures, comparisons between ethnic groups reveal linkage disequilibrium and highly variable patterns of allelic distributions for *HLA* genes [5,7].

The Taiwanese comprised the Minnan and Hakka people groups. The genetic profile of the Taiwanese shows many affinities to southern Asian populations [8]. HLA-B* 4601/B46 displays higher frequencies in Southern Han (15.4%), Singaporean (15.1%) and Vietnamese (13.2%) than in Northern Han (2.8%) [9]. There were a few studies that have focused on the association between *HLA* markers and disease using Southern Han Chinese data. Most of the studies found them a homogeneous group [8-11].

Recent advances in array-based SNP genotyping technologies have led to a revolution in the nature and scale of disease-association studies. These developed technologies yield economical ways to genotype one million SNPs across the entire genome in large-population studies. To date, direct experimental methods for typing *HLA* genes (via serology or PCR) remain laborious, expensive, and time consuming. This has limited large-scale studies involving *HLAs*[5,12]. To reduce the cost of these studies, researchers have begun to use data from SNPs around the *HLA* regions to make predictions concerning *HLA* allele types [13,14]. Leslie et al. [14] developed a statistical method based on identity by descent, which uses phase-resolved genotype data (i.e. full haplotype information was inferred by multiple SNPs) to predict *HLA* alleles. Li et al. [13] proposed a complementary method for predicting *HLA* alleles based on unphased genotype data. Given a training data set of unphased genotypes and their corresponding phased *HLA* alleles, they used a likelihood method to compute probabilities associated with all possible pairs of *HLA* alleles and their particular haplotypes. Because of high-throughput SNP-genotyping technologies, the method developed by Li et al. [13] analyzes many less-common *HLA* alleles. This results in more accurate predictions concerning *HLA* alleles.

After a decade of research, *HLA* prediction methodologies have achieved a high level of accuracy. For classical *HLA* class I and class II genes, there is typically a 97% accuracy when Caucasian populations are analyzed [13,14]. These types of studies, however, have not been rigorously applied to other ethnic groups [7]. Zhang et al. [7] revealed that predictive accuracies were poor if *HLA* alleles from non-Caucasian subjects were used as the training data set to build predictive *HLA* models for different ethnic groups. Hence, the African-specific *HLA*-prediction model has been developed [15]. Unfortunately, there is very little information concerning *HLA* allele prediction in Han Chinese populations. This need must be addressed before effective, ethnic-specific prediction model of *HLA* genes can be formulated.

Leslie et al. [14] achieved a high level of accuracy using data from the Haplotype Map (HapMap) Project as the training data. They validated their *HLA*-prediction model using genotypes and *HLA* allele information from the British 1958 birth cohort study. Unfortunately, genotype datasets with densities that approach the HapMap Project are not commonly available. As such, the use of chip data may represent an alternative method for constructing prediction model of *HLA* genes that are specific for Han Chinese populations.

Toward this goal, we applied the Li et al. [13] method to samples from Taiwan to build ethnic-specific prediction model of *HLA* genes for Han Chinese populations. This study comprised 437 Han Chinese with Affymetrix 5.0 and Illumina 550 K SNPs, of whom 214 also had data on Affymetrix 6.0 SNPs. All individuals were *HLA* typed at a 4-digit level resolution at 6 *HLA* loci and were used for training and testing the prediction model of *HLA* genes. To optimize these prediction models for classical *HLA* class I and class II genes, we addressed the following questions: 1) would there be differences in *HLA* allele distributions and optimal flanking regions between our Han Chinese data set and the HapMap Caucasian samples, 2) could MHC SNP data generated by different platforms yield comparably accurate *HLA* allele predictions, and 3) could imputation of untyped MHC SNPs improve the accuracy and robustness of the model? We provide practical recommendations concerning ethnic-specific prediction model of *HLA* genes regarding *HLA* gene regions, platforms, and imputation.

## Methods

### Ethics statement

Blood samples from 437 Han Chinese subjects residing in Taiwan were obtained from the Taiwan Han Chinese Cell and Genome Bank [16] and were used for this analysis. This study was approved by the Internal Review Board of Academia Sinica. A written informed consent was signed by every participant at his/her initial clinic visit. All individuals in this study were Han Chinese. All participants in this study have full Han ethnicity through both maternal and paternal grandparents and familial residence in the area of Taiwan for the last 3 generations. Our data consisted of three Taiwanese subgroups: Minnan (70%), Hakka (13%) and Mainlanders (14%). In one of our previous study for MHC SNPs in Han Chinese residing in Taiwan, we have shown that the Taiwanese population is homogeneous [17].

### DNA samples and MHC SNP data

Genomic DNA was extracted from blood using the Puregene DNA Isolation Kit (Gentra Systems, Minneapolis, USA). Four hundred and thirty seven samples were genotyped using the Affy 5.0 (Affymetrix, Santa Clara, CA, USA) and the Illumina 550 K chips (Illumina, San Diego, CA, USA). Two hundred fourteen of these samples were also genotyped using the Affy 6.0 chip. The Affy 5.0 chip contains 31,393 SNPs on chromosome 6 among 488,756 SNPs across 22 chromosomes. The Affy 6.0 chip contains 56,202 SNPs on chromosome 6 among 906,600 SNPs across 22 chromosomes. The Illumina 550 K chip contains 36,591 SNPs on chromosome 6 among 546,401 SNPs across 22 chromosomes. The National Center for Genome Medicine at Academia Sinica carried out all genotyping. All of the sample call rates were > 97%, and the mean individual-sample call rate was 98.4 ± 0.7%.

Within the extended human MHC region, which includes ~6 megabases (Mb) on chromosome 6p (position 28–34 Mb), the Affy 6.0 chip contains 2,203 SNPs, the Affy 5.0 chip contains 1,406 SNPs, and the Illumina 550 K chip contains 1,939 SNPs (Additional file 1). The intra-MHC region spanning the *HLA-A* gene at the telomeric end and the *HLA-DPB1* gene at the centromeric end actually harbors class III genes (which are also termed complement genes), in addition to both class I loci (*HLA-A, HLA-B, HLA–C*) and class II loci (*HLA-DRB1, HLA-DQB1, HLA-DPB1*).

### *HLA* genotyping

*HLA* genotypes were observed from our previous study [18]. Briefly, six classic *HLA* genes (*HLA-A*, *-B*, *–C*, *-DQB1*, *-DRB1*, and *-DPB1*) to a 4-digit resolution were analyzed in the present study. Among these genes, the *HLA-A*, *-B*, *–C*, *-DQB1*, and *-DRB1* alleles were genotyped using the Dynal RELI SSO typing kit (Dynal Biotech Ltd., Bromborough, Wirral, UK; now part of Life Platforms, Carlsbad, CA, USA). In brief, both exon 2 and exon 3 of class I genes (*HLA-A*, *-B* and *–C*) and exon 2 of class II genes (*HLA-DQB1* and *-DRB1*) were amplified by PCR using locus-specific primer sets. After amplification, PCR products were hybridized to sequence-specific oligonucleotide (SSO) probes that had been previously fixed to a nylon membrane in a linear array. SSO probes included 48 *HLA-A* probes, 61 *HLA-B* probes, 37 *HLA–C* probes, 41 *HLA-DQB1* probes, and 60 *HLA-DRB1* probes. The Pattern-Matching Program (Dynal Biotech, Ltd.) was used to interpret genotypes. Because the Dynal RELI SSO system lacked an *HLA-DPB1* genotyping kit, *HLA-DPB1* was genotyped using the Gold SSP *HLA-DPB1* High Resolution Kit (Invitrogen; now part of Life Platforms; California, USA). This genotyping technique is based on the sequence-specific primer amplification method. The *HLA-DPB1* genotyping was based on 48 PCR reactions for each DNA sample. The UniMatch software (Invitrogen) used the pattern of PCR amplification to interpret *HLA-DPB1* genotypes. While medium to high resolution typing results were obtained from those reverse SSO typing systems, several genotype combinations produced same reaction patterns. For these conditions, allele designation were assigned according to the most common alleles (allele frequency > 0.01) found in Taiwanese populations and southern Chinese populations as determined in the population studies of 13^th^ international histocompatibility workshop or to the allele with the lowest definition number. The four digit alleles detected at each *HLA* locus and allele frequencies in our Han Chinese data (n = 214) were listed in Additional file 2.

### *HLA* prediction methodology

To build prediction model of *HLA* genes using unphased genotypes, we adopted an estimating equation approach [13]. For each gene, the *HLA*-predictive methodology was then carried out as two separate procedures. The first procedure constructed a prediction model, whereas the second procedure validated the model generated by the first procedure. In the first procedure, a set of unphased genotypes was selected to build a prediction model. This selection process was evaluated using an objective function [13], which was the negative log-likelihood of the *HLA* allele given unphased genotypes (based on the Akaike Information Criterion) [19]. Genotype selection was then performed using the forward-selection and backward-elimination scheme. This started with genotypes associated with an *HLA* allele and we gradually added one genotype at a time. The second procedure validated the prediction model using an independent set of samples. For these independent samples, unphased genotypes and phased *HLA* alleles were provided. Following the parsimonious rule, the best prediction model should use the smallest possible flanking region and the fewest possible SNPs to generate the most accurate predictions.

### Scenarios

#### Differences in HLA allele frequency distributions and flanking-region sizes between ethnic groups

*HLA* alleles and their allelic distributions differ substantially between ethnic groups, reflecting their recent evolutionary histories [7,20]. Furthermore, *HLA* genes cover different regions on chromosome 6p and include various numbers of SNPs [12]. Here we explored allele frequency distributions within our Han Chinese samples and within Caucasian samples from the HapMap Project [21]. For each *HLA* allele, we used chi-square and Fisher’s exact tests to determine whether *HLA* allele proportions were different between these two populations. Following Li et al.’s [13], we assessed flanking regions that extended ±10 kb to ±400 kb to construct the prediction model of *HLA* genes. Within the Han Chinese population, the most appropriate flanking region for each *HLA* gene was determined using the parsimonious rule described above. Furthermore, we compared flanking-region sizes derived from our Han Chinese population (from the Affy 5.0 chip) with flanking-region sizes from the Caucasian population in Li et al.’s [13].

#### Different platforms

Three platforms were used in this study: 1) the Affymetrix Genome-Wide Human SNP Array 5.0 (Affy 5.0) [22,23], 2) the Affymetrix Genome-Wide Human SNP Array 6.0 (Affy 6.0), and 3) Illumina’s HumanHap550 Genotyping BeadChip (Illumina 550) [24,25]. To measure the compatibility between these platforms we compared overlapping SNP data from each pair of platforms. Genotype data concordance was computed using Cohen’s kappa coefficient [26], a measurement commonly used to assess the degree of concordance between two independent groups [7]. Values of kappa > 0.9 generally indicate excellent reliability [27]. For each platform pair, genotype concordance was evaluated between all observed SNPs and all imputed SNPs. For each pair, we also compared variation between genotypes that were selected during the *HLA* model-building process. This was done to determine whether selected genotypes were platform specific. Variation was defined as $\frac{\cup \left(\mathrm{pla}t_{i},\mathrm{pla}t_{j}\right)-\cap \left(\mathrm{pla}t_{i},\mathrm{pla}t_{j}\right)}{\cup \left(\mathrm{pla}t_{i},\mathrm{pla}t_{j}\right)}$, where *plat*_*i*_ and *plat*_*j*_ are two different platforms, ∪(*plat*_*i*_, *plat*_*j*_) is the union of the SNPs for two different platforms, and ∩(*plat*_*i*_, *plat*_*j*_) is the intersection of the SNPs for two different platforms.

### Imputation

“Genotype imputation” is the term used to describe the process of imputing genotypes that are not directly assayed in a sample of individuals [28-30]. Genotype imputation has become a routine practice in genome-wide association studies (GWASs). Here we evaluated the usefulness of imputed genotypes in constructing prediction model of *HLA* genes. For data consistency and optimal-imputation performance, we used the MaCH [31] software and the Chinese Han Beijing (CHB) + Japanese Tokyo (JPT) data set [32] as a reference. This was used to impute genotypes beyond our SNPs, which were derived from the HapMap Project. Standard GWAS quality-control filters (e.g., minor-allele frequency < 0.01, genotype call rates < 0.95, and a significant departure from Hardy-Weinberg equilibrium at p < 10^–4^) are usually sufficient before genotype imputation [33-35]. We applied these recommendations, therefore, and checked our genotype data for all SNPs within the MHC region. Furthermore, each imputed SNP that were included in this study should have an imputation posterior probability from MaCH > 0.8, call rate > 0.95, and minor-allele frequency > 0.01. The three steps used for genotype imputation were as follows: 1) apply standard GWAS quality-control filters to our MHC SNPs, 2) use MaCH software to impute genotypes beyond MHC SNPs that were determined in step 1, and 3) check the imputation posterior probabilities from MaCH for the imputed SNPs that were determined in step 2.

#### Cross-validation

Before beginning the *HLA* prediction analysis, we divided the data into multiple partitions for cross-validation (CV). All 437 individuals were *HLA* typed at a 4-digit level resolution at 6 *HLA* loci and were used for training and testing the prediction model of *HLA* genes. We used a 10-fold CV in the study, the data set was divided into a training data set (9/10 of the 437 data) and a testing data set (1/10 of the 437 data). The testing accuracy was assessed according to the comparison between the original *HLA* alleles and predictive *HLA* alleles for each sample. If the pair of *HLA* alleles between the original *HLA* alleles and predictive *HLA* alleles was the same, the predictive value was assigned 1. If a half pair of *HLA* alleles was the same, then the predictive value was assigned 0.5, else it was assigned 0. For each CV subset, the testing accuracy was calculated for the testing set and defined as $\frac{T_{v}}{N_{v}}$ , where *T*_*v*_ is the number of correctly predicted samples (the sum of the predictive values from each predicted sample) in the testing set and *N*_*v*_ is the total number of samples in the testing set. The average testing accuracy is the mean of the 10 CV subsets and indicates how well the constructed *HLA* model predicts the *HLA* alleles. The *HLA* prediction can be performed without CV, but CV execution can avoid over-fitting the prediction model and can save both time and costs associated with attaining an independent set of samples for validation [36]. We built prediction model of *HLA* genes, therefore, using 10-fold CV.

#### Confidence threshold (CT)

For each sample within the testing data set, probability values were assigned to each possible pair of *HLA* alleles given a particular haplotype. These values were based on the provided unphased genotypes and phased pairs of *HLA* alleles. After assigning probabilities, we selected the pair with the maximum probability, if that probability exceeded a pre-specified CT. When CT is set to be 0, it means that the call rate is 100% (i.e., all samples will be predicted). If CT is set to be 0.5 (or any value greater than 0), only samples for which the maximum predictive probability exceeds CT will be used. Here we set CT to be 0, 0.5, or 0.9 to evaluate the effects of CT on constructing prediction model of *HLA* genes. If a higher CT is applied, the prediction model of *HLA* gene results in greater prediction accuracy.

## Results

### *HLA* allele frequency distributions and flanking-region sizes

We calculated allele frequency distributions for the six classical *HLA* genes using 214 samples that were genotyped using three different genomic platforms (Affy 5.0, Affy 6.0, and Illumina 550 K chips). Analyzed samples were from Han Chinese subjects living in Taiwan provided by the Han Chinese Cell and Genome Bank [16]. We also analyzed 180 Caucasian samples from the HapMap Project, although *HLA-DPB1* data were not available for these samples. The Caucasian samples from HapMap were CEPH samples and only founders were included. The most polymorphic *HLA* locus was *HLA-B*. Across the *HLA-B* region, we observed 44 alleles in our Han Chinese population and 32 alleles in the HapMap Caucasians. To address the issue of a sparse contingency table, the allele frequency for *HLA-A, -B, –C, -DQB1*, and *-DRB1* genes between Caucasians and Han Chinese was compared using program clump (http://www.smd.qmul.ac.uk/statgen/dcurtis/software.html) [37]. All distribution differed significantly between the Caucasians and Han Chinese at the adjusted 5% level using Bonferroni’s correction. We confirmed the previous finding, therefore, that *HLA* allele frequency distributions differ extensively across populations. As such, predictive *HLA* models that are built using *HLA* alleles from one population may generate poor predictions concerning a different ethnic population. Ethnic-specific prediction model of *HLA* genes are generally preferred [7].

**Figure 1:**
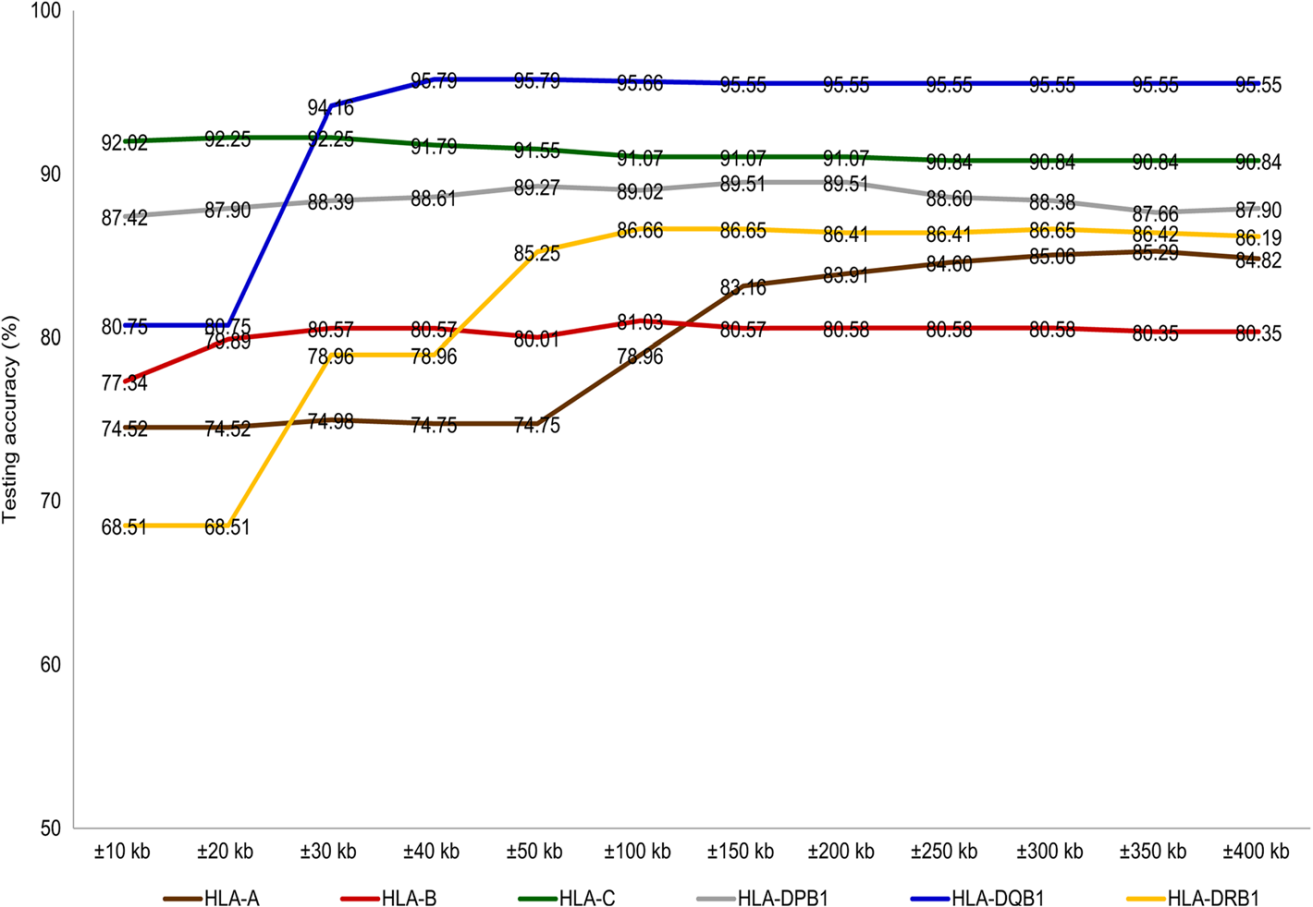
**Testing accuracies associated with different flanking-region sizes.** For each of the six *HLA* genes (colored lines), testing accuracies are shown for increasing flanking-region sizes. Data from the Affy 6.0 chip are shown without imputed SNPs.

Using the *HLA*-predictive methodology described above, we evaluated testing accuracies given various flanking-region sizes following Li et al.’s [13]. We assessed flanking regions that were extended from ±10 kb to ±400 kb (the testing accuracies remained almost the same after ±300 kb across 6 classical *HLA* genes). Within the Han Chinese population, the most appropriate flanking region for each *HLA* gene for prediction was determined using the parsimonious rule. For most of the prediction models (*HLA-A*, *-B*, *–C*, *-DQB1*, *-DRB1*, and *-DPB1*), trends in the data indicated that more-accurate results were obtained when larger flanking regions were used (Figure 1). The number of predictive SNPs selected also increased with the size of the flanking region. The most dramatic effects on testing accuracy were observed for *HLA-DRB1* and *HLA-DQB1*. When a ±10 kb flanking region was used, *HLA-DRB1* yielded the lowest testing accuracy (68.51%). This accuracy increased to >85% when the flanking region was increased to ±50 kb. For *HLA-DQB1*, testing accuracy ranged from 80.75% (±10 kb flanking region) to 95.79% (±40 kb region). Once the size of the flanking region increased to ±30 kb, the testing accuracy of *HLA-DQB1* increased to >90%. Testing accuracies for *HLA-C* (92.02%) and *HLA-DPB1* (87.42%) were satisfactory at ±10 kb. Notably, testing accuracies for *HLA-A* (74.52%) and *HLA-B* (77.34%) were particularly low with ±10 kb flanking regions. Using the size ± 150 kb, however, raised the accuracy to >80% for both genes. Flanking-region results across the different genotyping platforms (Affy 5.0, Illumina 550 K, and a union of the three platforms) are provided in Additional file 3.

We also compared flanking-region sizes derived from our Han Chinese population with those from the Caucasian population described previously by Li et al.’s [13]. *HLA-A* gene required the largest flanking regions (±200 kb for the Han Chinese and ±350 kb for the Caucasian samples, respectively). For *HLA-C*, a ±20 kb region leads to an accuracy of 90.87% which is the best model by our definition for the Han Chinese, however, it was ±180 kb for the Caucasian population (Li et al. [13]). The size difference in the optimal flanking region was even more dramatic for *HLA-DQB1*, which was ±40 kb for the Han Chinese and ±300 kb for the Caucasians. The comparisons of flanking region were based on Affy 5.0 chip (Table 1) which was used by Li et al.’s [13] (data not shown). D’ values was also listed in Table 1. The flanking region sizes from Caucasian described previously by Li et al.’s [9] were different from ours (Affy 5.0) probably due to population stratification. From our chip data, the range of the highest D’ for the *HLA* markers was between 0.47 and 0.83 without imputation. It was 0.51 to 0.85 with imputation. *HLA-C* had the highest D’ regardless of imputation and platform while *HLA-DPB1* had the lowest D’. *HLA-A* had the longest flanking region regardless imputation or not while HLA-DQB1 had the shortest flanking region. The length of the *HLA* gene and the LD intensity is negative. However, the LD intensity might not be related to the flanking region size for the optimized predictive models for each platform (Table 1).

**Table 1:** Flanking regions associated with optimized prediction models for each platform

				**Flanking region**^**2 **^**(SNPs**^**3**^**/total SNPs**^**4 **^**(D’)**^**5**^**)**							
				**Without imputation**				**With imputation**			
**Gene**	**Start**^**1**^	**Stop**^**1**^	**Length (bp)**	**Affy5.0**	**Affy6.0**	**Illumina550K**	**Union**	**Affy5.0**	**Affy6.0**	**Illumina550K**	**Union**
*HLA_A*	30,018,310	30,021,632	3,322	200 K (14/122 (0.76))	350 K (16/299 (0.69))	150 K (14/121 (0.79))	200 K (16/329 (0.74))	150 K (19/352 (0.81))	150 K (17/364 (0.80))	200 K (17/515 (0.73))	150 K (16/366 (0.80))
*HLA_B*	31,429,630	31,432,914	3,284	150 K (20/100 (0.61))	100 K (20/123 (0.66))	100 K (18/131 (0.59))	100 K (21/237 (0.64))	100 K (24/282 (0.67))	40 K (21/110 (0.71))	30 K (22/102 (0.71))	40 K (22/110 (0.71))
*HLA_C*	31,344,509	31,347,834	3,325	150 K (17/104 (0.64))	20 K (14/25 (0.83))	10 K (13/22 (0.82))	30 K (17/95 (0.79))	30 K (19/137 (0.83))	20 K (18/88 (0.85))	20 K (16/88 (0.85))	20 K (18/88 (0.85))
*HLA_DPB1*	33,151,738	33,162,954	11,216	100 K (13/88 (0.49))	150 K (15/154 (0.47))	100 K (15/120 (0.52))	150 K (18/280 (0.47))	20 K (34/113 (0.79))	100 K (33/352 (0.54))	150 K (35/452 (0.51))	100 K (35/355 (0.54))
*HLA_DQB1*	32,735,635	32,742,419	6,784	40 K (12/15 (0.61))	40 K (11/30 (0.71))	30 K (12/13 (0.72))	30 K (13/29 (0.70))	30 K (16/52 (0.69))	30 K (16/52 (0.69))	30 K (15/52 (0.69))	30 K (15/52 (0.69))
*HLA_DRB1*	32,654,527	32,665,559	11,032	200 K (16/108 (0.60))	100 K (16/27 (0.73))	150 K (17/108 (0.61))	150 K (21/204 (0.61))	150 K (25/325 (0.61))	100 K (22/91 (0.76))	200 K (25/523 (0.58))	150 K (24/326 (0.62))

^1^Based on NCBI build 36.3.

^2^Flanking region for the most accurate prediction model.

^3^Number of SNPs within the region that were selected for the model (based on predictive power).

^4^Total number of SNPs in the MHC and *HLA* regions.

^5^Hedridge’s multialleic D.

### Predictive accuracies without imputation

The overlapping data between each pair of platforms was quite few (Additional file 1). Affy 6.0 had the most SNPs within the MHC regions, whereas Affy 5.0 had the fewest (Additional file 1). Additional file 4 shows kappa coefficients between paired platforms, with respect to observed genotypes. Comparing the two Affymetrix arrays, the kappa coefficient was as high as 0.9926 for the genotypes present on both arrays. This high level of concordance indicated high-quality genotyping, which was further supported by the determination of comparable genotypes between platforms.

**Figure 2:**
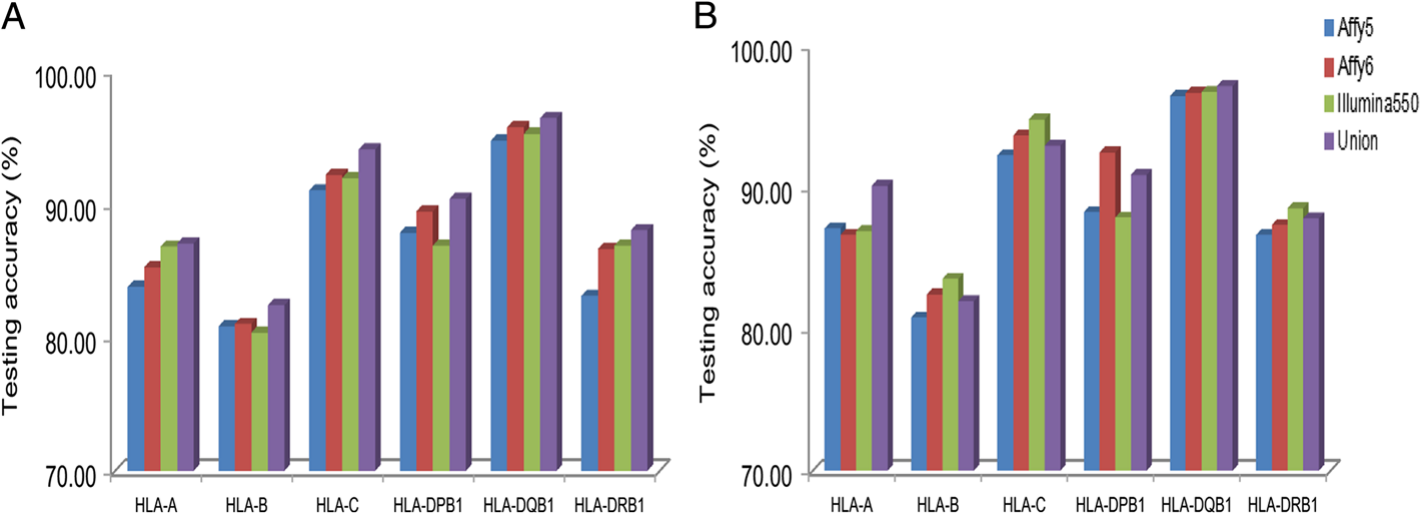
**Testing accuracies for optimized models generated from each genotyping platform.** Testing accuracies and call rates are shown for the six *HLA* genes (for CT = 0). Values from each of the three genotyping arrays as well as from the union of the three arrays (colored bars) are shown both without **(A)** and with **(B)** imputed SNPs.

In general, Union generated more accurate *HLA*-allele predictions than did each of the individual platforms. For CT = 0, the average testing accuracy was 89.78% with Union, but 86.92%, 88.42%, and 88.06% for Affy 5.0, Affy 6.0, and Illumina 550 K, respectively (Figure 2A). These findings were consistent with Zhang et al. [7], who suggested that higher SNP densities increased the accuracy of *HLA*-allele predictions.

In comparisons between the three genotyping platforms, Affy 6.0 generated the most-accurate *HLA*-allele predictions. For example, Affy 6.0 was 3.52% more accurate than Affy 5.0 at the *HLA-DRB1* locus; and Affy 6.0 was 2.58% more accurate than Illumina 550 K at the *HLA-DPB1* locus. It was possible that Affy 6.0 did have the highest density of genotypes within the MHC region. For CT = 0, the overall highest testing accuracy was obtained with Affy 6.0 and *HLA-DQB1* (95.79%), whereas the lowest accuracy was from Illumina 550 K and *HLA-B* (80.37%; Figure 2A). By applying a CT of 0.9 to the maximum probability of all possible pairs of *HLA* alleles, the highest accuracy was increased to 98.62% at the *HLA-C* locus (with a call rate of 77.47% from Illumina 550 K), whereas the lowest accuracy was elevated to 87.67% at *HLA-B* (with a call rate of 64.94% from Affy 5.0; Additional file 5). The range of accuracies generated by Illumina 550 K-based prediction models was more dramatic across *HLA* loci than was seen with the other genotyping platforms. With CT = 0, the accuracy of the Illumina 550 K prediction model was only 80.37% at the *HLA-B* gene but was 95.29% at *HLA-DQB1*. The Affy 5.0 predictions were 0.45% and 0.96% more accurate than Illumina 550 K for the *HLA-B* and *HLA-DPB1* genes, respectively. Illumina 550 K predictions were 1.56% and 0.27% more accurate than Affy 6.0 for the *HLA-A* and *HLA-DRB1* genes, respectively (Figure 2). These results agreed with Zhang et al.’s [7], who showed that slight improvements associated with Affy 5.0 and Illumina 550 K may be attributed to unique SNPs on these platforms. Overall, accuracies of these prediction models were generally comparable across genotyping platforms. Details concerning testing-accuracy results and the call rate for predictions across different platforms and different CTs are provided in Additional file 5.

The investigation into the sufficient flanking regions (i.e., the shortest stretch of flanking genomic sequence that yielded the most accurate *HLA*-allele prediction) was performed. The shortest sufficient flanking region was identified at the *HLA-C* locus using Illumina 550 K (±10 kb). The length of *HLA-C* was 3,325 bp and this sufficient flanking region covered 22 SNPs, of which 13 were incorporated into the *HLA-C*-prediction model (testing accuracy of 92.01% at CT = 0). The longest sufficient flanking region was ±350 kb at *HLA-A* when Affy 6.0 data were used (Table 1). Within this region, there were 299 SNPs, of which 16 were incorporated into the *HLA-A*-prediction model (testing accuracy of 85.29% at CT = 0).

For each *HLA* gene, we further assessed between-platform overlap of the genotypes which were incorporated into the *HLA* prediction model (Additional file 6). The largest percentage of overlapping genotypes for data without imputed SNPs was 21.36% at *HLA-DRB1* when Affy 6.0 and Union were compared. These findings suggest that different platforms might use unique SNPs to select platform-specific genotypes. These genotypes were then used to build the different prediction model of *HLA* genes. We listed the SNPs that best predict the *HLA* alleles using the genotype information currently available from different platforms (Additional file 7) and listed the alleles of the SNPs that predict *HLA* allele types (Additional file 8).

### Predictive accuracies with imputation

We also performed comparisons between each pair of genotyping platforms to assess the concordance of SNPs with imputation. The Kappa coefficients were at least 0.9587 for these measurements, which were based on the genotypes originally observed and then imputed on Affy 5.0 and Illumina 550 K (Additional file 4). These results implied high imputation quality and comparable genotypes across the platforms.

In general, Union (average testing accuracy of 90.17% at CT = 0) generated more-accurate *HLA*-allele predictions than the three individual platforms (for CT = 0, average testing accuracies were 89.90%, 88.61%, and 89.75% for Affy 6.0, Affy 5.0, and Illumina 550 K, respectively; Figure 2B). As such, higher SNP densities may increase the accuracy of genotype imputation and, therefore, the accuracy of the final prediction. These results are consistent with Zhang et al.’s [7]. However, Illumina 550 K and Affy 6.0 sometimes yielded better accuracy than the union arrays in our results. One of the possible reasons might be due to “non-concordance” among these platforms. The kappa coefficients ranged from 95.87% to 97.62% (Additional file 4). It’s likely that those with low concordance genotypes might decrease imputation accuracies.

In the comparisons between the three genotyping platforms with imputation, Affy 6.0 predictions were generally more accurate for *HLA* alleles (up to 4.23% more accurate than Affy 5.0, and 4.61% more accurate than Illumina 550 K at the *HLA-DPB1* locus; Figure 2B). Among these models (CT = 0), the *HLA-DQB1* locus had the highest testing accuracy (96.75%, from Illumina 550 K), whereas the *HLA-B* locus had the lowest (80.81%, from Affy 5.0). By applying a CT value of 0.9 to the maximum probability of all possible pairs of *HLA* alleles, the highest accuracy was increased to 99.09% at the *HLA-C* locus (with a call rate of 82.40%, from Illumina 550 K), and the lowest accuracy was increased to 91.58% at the *HLA-A* locus (with a call rate of 41.65%, from Affy 5.0). Besides *HLA-B* from Affy 5.0, accuracy improvements based on this CT adjustment were most dramatic for the *HLA-DRB1* gene, which rose from 86.67% to 95.90% when CT was changed from 0 to 0.9. Affy 5.0 (CT = 0), however, generated a prediction that was 0.45% more accurate than Affy 6.0 at the *HLA-A*. Besides *HLA-DPB1* (with a more testing accuracy of 4.61%), Illumina 550 K generated predictions that were 0.27%, 1.10%, 1.11%, 0.05% and 1.19% more accurate than Affy 6.0 at the *HLA-A*, *-B*, *-C*, *-DQB1* and *-DRB1* loci, respectively (Figure 2). These findings are consistent with Zhang et al.’s [7], which showed that prediction improvements from the Affy 5.0 and Illumina 550 K array data may be attributed to SNPs that are exclusively represented on these platforms. Details concerning testing accuracies and prediction call rates across different platforms and different CTs are listed in Additional file 5.

For each *HLA* locus, we also assessed the sufficient flanking region with imputation. One of the shortest flanking regions was identified by Affy 5.0 at the *HLA-DPB1* locus (±20 kb) (Table 1). This region covered 113 SNPs, of which 34 were selected for the *HLA-DPB1*-prediction model (testing accuracy of 88.28% at CT = 0). The other shortest flanking regions were identified by Affy 6.0, Illumina 550 K and Union at the *HLA-C* locus (±20 kb). The longest sufficient flanking region was ±200 kb at *HLA-A* from Illumina 550 K; Table 1). Within this region there were 515 SNPs, of which 17 were used in the *HLA-A*-prediction model (testing accuracy of 86.93% at CT = 0).

For each *HLA* gene, the amount of overlap among genotypes used by the different prediction models was at most 60.08% (Additional file 6). Imputation, therefore, seemed to reduce discrepancies among the different platforms.

### Differences in predictive accuracies with and without imputation

We performed comparisons between each pair of genotyping platform to assess overall concordance between SNPs with and without imputation. The lowest kappa coefficient was 0.9922, which was derived from Affy 5.0 genotypes (Additional file 4). These results demonstrated high imputation quality, even though nearly 10,000 SNPs within this 6-Mb region were imputed from the Affy 5.0 chip.

Comparisons of testing accuracies among prediction models with and without imputation across different platforms revealed that prediction models built with imputed SNPs were more accurate than those built without imputed SNPs (average accuracies of 89.61% and 88.30%, respectively, for CT = 0; Figure 2). Prediction improvements associated with imputation of the *HLA-DRB1* alleles on Affy 5.0 were more dramatic than those seen with other *HLA* genes across the three platforms. Imputation at this locus elevated prediction accuracy from 83.14% to 86.67%. These results essentially agree with Zhang et al.’s [7], who suggested that more SNPs generally increased *HLA* allele-prediction accuracy.

For CT = 0, the Union platform with imputation had the highest testing accuracy (97.18%) for *HLA-DQB1* alleles, whereas Illumina 550 K without imputation had the lowest testing accuracy (80.37%) for *HLA-B* alleles. By applying a CT of 0.9 to the maximum probability of all possible pairs of *HLA* alleles, the highest accuracy was elevated to 99.09% at the *HLA-C* locus with Illumina 550 K (with imputation and a call rate of 82.40%). This CT adjustment increased the lowest accuracy to 87.67% at the *HLA-B* locus with Affy 5.0 (without imputation and with a call rate of 64.94%; Additional file 5).

Comparing testing-prediction accuracies with and without imputation among the different platforms, indicated genotype variation generally decreased when imputed SNPs were used to build the prediction model of *HLA* genes. For each *HLA* gene among the different platforms, the percentage overlap among genotypes that were selected to build the models increased 25.02% on average with imputation (Additional file 6). These results may have stemmed from using a consistent set of HapMap SNPs, which could have minimized discrepancies among different genotyping platforms.

## Discussion

Because classic technologies for the direct typing of *HLA* alleles are economically infeasible, we instead applied a method developed by Li et al. [13], which identifies specific *HLA* alleles based on their corresponding unphased genotypes. We used this method to build prediction model of *HLA* genes for *HLA* class I (*HLA-A*, *HLA-B*, and *HLA-C*) and class II (*HLA-DRB1*, *HLA-DQB1*, and *HLA-DPB1*) genes. We compared allele frequency distributions of *HLA* genes between the Han Chinese from Taiwan and the Caucasian population (from the HapMap Project) Allele frequencies are different between our Han Chinese and the Caucasian samples p < 0.0001 with chi-square and Fisher’s exact tests. That is because some of the alleles are the same but just at different frequencies. The most polymorphic *HLA* locus was *HLA-B*. Within the *HLA-B* region, we identified more alleles in the Han Chinese population (44 alleles) than in the Caucasian HapMap population (32 alleles). This allele frequency difference supports the use of the method of Li et al. [13], as well as the use of a Taiwan-based data set to build ethnic-specific prediction model of *HLA* genes for a Han Chinese population. We have built a number of models for predicting class I and class II *HLA* genes at a four-digit resolution and have examined critical parameters associated with these models (e.g., the sufficient flanking region, platform accuracy, and the effect of imputation). We found that our models accurately predict these alleles within a Han Chinese population. Results from this study have direct implications for detailed analyses of *HLA*-related disease associations.

*HLA* genotypes were observed from our previous study [15]. Briefly, six classic *HLA* loci (*HLA-A*, *-B*, *-C*, *-DQB1*, *-DRB1*, and *-DPB1*) to a 4-digit resolution were analyzed in the present study. We did not include *DQA1* or *DRB3*, *4*, and *5* in this study, partly because of the unavailability of genotyping kits and partly because that their LD with corresponding *DQB1* or *DRB1* alleles would be too tight to be delineated. In some of our previous studies, we reported that HLA-B*15:02 was highly associated with Carbamazepine-induced Stevens-Johnson syndrome and toxic epidermal necrolysis [38-41].

*HLA-B* had the highest overlapping density between our Han Chinese and the Caucasian population (Li et al. [13]), the number of predictive SNPs was 69 for the former and 86 for the latter. Among the 155 predictive SNPs, the number of the overlapping SNPs was 11. *HLA-DRB1* had the lowest overlapping density (4 overlapping SNPs), in which, our Han Chinese required 51 predictive SNPs and the Caucasian population required 41 predictive SNPs.

In an attempt to differentiate between the effects of the Li et al.’s algorithm and the Han Chinese panel, we examined the *HLA* predictive accuracies between the two algorithms proposed by Li et al. (2011) and Leslie et al. (2008) and found that the paper reported by Li et al. (2011) has shown that the *HLA* predictive accuracies for the two algorithms were in general comparable using the British 1958 birth cohort data. We also applied the Li et al.’s method to HapMap CEU/CEPH data, we compared the accuracies derived from Li et al.’s method with those by Leslie et al.’s We built *HLA-B* and *HLA-DQB1* models (the worst and the best performance models, respectively) using leave-one-out CV adapted by Leslie et al. With a CT of 0.9, accuracy of *HLA-B* was 100% from Li et al. and 95% from Leslie et al. For *HLA-DQB1*, accuracy was 99% from Li et al. and 99% from Leslie et al. Therefore, we concluded that the prediction models were generally comparable for the two methods.

In this study, the SNP genotype imputation is used as material preparation for the *HLA* allele prediction. The imputation of the SNP genotypes refers to increasing the SNP density by adding SNPs that originally are not incorporated in the array chips but in the HapMap project data (Chinese Han Beijing (CHB) + Japanese Tokyo (JPT) in our case). The HapMap project data mainly includes Chinese Han Beijing (CHB) belonging to northern Chinese Han subgroup. Imputation was carried out to increase the diversity of alleles covered in our training data and also help to boost the accuracy for predicting *HLA* genotypes. The missing rate for our *HLA* data was very low (0.23%), therefore, no imputation was performed for the *HLA* genotype data. Here we determined whether a denser collection of SNPs would generate more-accurate *HLA* allele predictions. The MaCH software, which we used to impute our chip data sets and thus build greater SNP density, uses data from the HapMap Project and/or the 1000 Genomes Project (http://www.1000genomes.org) as references*.* We found that prediction model of *HLA* genes that were built with imputation typically provided greater prediction accuracy**,** which underscores the positive effect of using a higher density of SNPs. Therefore, it is possible to devise a new customized SNP array that includes all of the selected SNPs consisting those of genotyped or imputed SNPs for the *HLA*-prediction model to increase the prediction accuracy. Additionally, we used our genotype data from Affy 6.0 (the densest chip data) to build *HLA-B* and *HLA-DQB1* models (the worst and the best performance models, respectively) using a 2-fold CV. The imputation was carried out separately for training and testing data set. We then compared the results with those obtained by imputation simultaneously for the two data sets. The testing accuracy of *HLA-B* for separated imputation was 90% and it was 89% for simultaneous imputation. For *HLA-DQB1*, the testing accuracies for separated imputation and for simultaneous imputation were both 96%.

By increasing CT to 0.5 or 0.9, levels of prediction accuracy approached 100% in our ethnic-specific prediction model of *HLA* genes for Han Chinese populations (Additional file 5). We analyzed the effect, therefore, of different reference data sets beyond our Affy 6.0 genotype data. When using the 1000 Genomes Project as the reference at CT = 0, we found that testing accuracy concerning the *HLA-C* gene decreased by 3.02% (down to 90.17%). Similar trends were observed at the other five *HLA* loci. Rare SNP variants within data from the 1000 Genomes Project may have increased the number of mismatched genotypes under imputation.

To generate more-accurate prediction models, we varied sample sizes for both the training and testing data sets. We used our genotype data from Affy 6.0 to build prediction model of *HLA* genes using 2-, 4-, and 10-fold CV. With CT = 0 (i.e., a call-rate of 100%) we obtained a consistent sample size for the different CVs. To eliminate the effect of sample size, therefore, we compared effects of CV at CT = 0. Additional file 9 shows the estimates for the testing accuracy that was obtained from validation analyses of prediction models built using 2-, 4-, and 10-fold CV (for CT = 0). The *HLA-DQB1* locus with 10-fold CV had the best testing accuracy (95.55%), whereas the *HLA-B* locus with 2-fold CV had the lowest testing accuracy (76.64%). At the 2-fold CV, testing accuracies ranged from 76.64% (*HLA-B*) to 94.39% (*HLA-DQB1*). By increasing the CV to 10-fold, testing-accuracy levels approached 95.55% (*HLA-DQB1*). For *HLA-A*, this improvement was most dramatic, as prediction models trained on the 2-fold CV had an accuracy of 80.84%, whereas training on the 10-fold CV resulted in an 85.29% accuracy. Similar trends were observed for the other five *HLA* loci. These trends may reflect the large size of the sample, which would include a sufficient number of *HLA* alleles in the training data set, thereby increasing predictive accuracy. Although the level of CV influenced the testing accuracy, these changes were minimal. We conclude, therefore, that our ethnic-specific prediction model of HLA genes is not affected by different CVs.

For *HLA-B* alleles, testing accuracies were generally not as high as others. One of the possible reasons was that there were large number of *HLA-B* allele with four digit and most of them with very low frequency. Hence, our training data could not sufficiently capture the variability. We also compared testing accuracies among predictive models with and without imputation across different platforms, the results revealed that predictive models built with imputed SNPs were more accurate than those without (average accuracies of 89.61% and 88.30%, respectively, for CT = 0; Figure 2). Therefore, proxy SNPs might improve testing accuracies. To generate more-useful prediction model of *HLA* genes, therefore, one should take into account that the distribution of these rare *HLA* alleles varies between the training and testing data sets. As such, applying the current *HLA*-predictive method to a large population that contains relatively rare *HLA* alleles in the training data set might improve the performance of ethnic-specific prediction model of *HLA* genes.

The common *HLA* typing experiment is not restricted to a single *HLA* allele but to a group of known *HLA* alleles having the same pattern within the common allele. Therefore, it is hard to determine rare alleles. However, we feel the prediction model is a useful tool for pre-screening disease association with common *HLA* alleles using chip data from GWAS. In the case of any significant association by the model predicted common alleles, it is understood that further *HLA* testing would be necessary. The *HLA* allele assignment was according to the most common alleles (allele frequency > 0.01) found in Taiwanese populations and southern Chinese populations as determined in the population studies of 13^th^ international histocompatibility workshop.

As mentioned above, we focused on prediction model of *HLA* genes that were generated using 214 Han Chinese samples from Taiwan, each of which were genotyped using the Affy 5.0, Affy 6.0, and Illumina 550 K chips. To assess the effect of the sample size for predicting, we analyzed prediction models generated using 437 Han Chinese samples (which included the original 214 samples) that were genotyped using the Illumina 550 K chip. Prediction model of *HLA* genes built from 437 samples (average testing accuracy was 90.36%) performed better than that from the 214-sample (average testing accuracy was 86.84%) across all *HLA* loci. As such, larger sample sizes seem to increase *HLA*-predictive accuracy. The sample size effects was confirmed by the previous finding from Jia et al. [11]. In our previous study [17], the profiles of haplotype blocks for the Caucasian and Taiwanese populations were apparently different in LD structure. Our *HLA* typing experiment assignment was according to the most common alleles (allele frequency > 0.01), but the Caucasian *HLA* data (Li et al. [13]) did not filter rare alleles. Inaccurate predictions might have resulted from rare *HLA* alleles, which were defined as those appeared less than three times in the training data set [13]. These rare *HLA* alleles might affect the first procedure of *HLA*-predictive methodology [13]. Hence, sample size, LD and configuration of allelic repertoire might be all possible reasons for the difference in the size of optimal flanking region for our Han Chinese and the Caucasian data.

We offer a web-based service on the *HLA* predictive models using Li’s algorithm to help other researchers to predict the *HLA* allele types and the corresponding probabilities of their subjects if the researchers provide the SNPs information files on the *HLA* region to us. The link is at the following (http://www.csjfann.ibms.sinica.edu.tw/eag/programlist/program_list.html).

## Conclusions

After a decade of research, many *HLA* genes are known to have specific immunological functions. Experimental methodologies that link SNPs to *HLA* alleles offer considerable savings (both in time and expense) over direct *HLA*-typing technologies and make large-scale investigations into *HLA* variation feasible. Although *HLA* allelic distributions differ among human populations, the majority of existing prediction model of *HLA* genes is based on Caucasian samples. By genotyping a large number of Han Chinese samples, we have uncovered many *HLA* alleles that are unique to this population and have built ethnic-specific prediction model of *HLA* genes. Our training data set covers many uncommon and ethnic-specific alleles within *HLA* loci, substantially raising predictive accuracies concerning the testing of data sets.

Specific methodological parameters investigated in this study (e.g., sample size, SNP density, and imputation) each were factors in generating prediction model of *HLA* genes that were specific for a Han Chinese population. We achieved good predictive accuracy for the *HLA-A*, *-B*, *-C*, *-DRB1*, *-DQB1*, and *-DPB1* genes within our Han Chinese samples. Using SNP data from the Affymetrix Genome-Wide Human SNP Array 5.0, the Affymetrix Genome-Wide Human SNP Array 6.0, the Illumina HumanHap550 BeadChip, or a union of these three technological platforms, we generated efficient prediction model of *HLA* genes for determining *HLA* alleles within a Han Chinese population. Our novel predictive tools may help identify genetic risk factors for immune-related diseases. Furthermore, these findings will enable researchers to investigate *HLA* allelic variations among a broader range of human populations.

## Competing interests

The authors declare that they have no competing interests.

## Authors’ contributions

ARH and SWC contribute equally. ARH, SWC, CLH, CCC and CSJF contributed to the statistical analysis. ARH and CSJF drafted the manuscript. PLC, CCC and SWC contributed to help to draft the manuscript with ARH. PLC, CCC, WSY, JYW, YTC and TCC contributed to collecting the data. All authors read and approved the final manuscript.

## Supplementary Material

Additional file 1 Overlapping SNPs between the HapMap Project and three genotyping platforms within the extended MHC region. Click here for file

Additional file 2 **List of the *HLA *** **alleles and allele frequencies in our Han Chinese data (n = 214).** Click here for file

Additional file 3 **Testing accuracies associated with different flanking-region sizes.** For each of the six *HLA* genes (colored lines), testing accuracies for various flanking-region sizes are shown. Data using the Affy 5.0 (A), Illumina 550 K (B), and Union (C) chip data sets are shown. [Union refers to a union of data from the three platforms (Affy 5.0, Affy 6.0, and Illumina 550 K)]. Click here for file

Additional file 4 Kappa coefficients of observed or imputed SNP genotypes within the MHC region. Click here for file

Additional file 5 **Testing accuracies and call rates from optimized models for each platform at different CT settings.** For each of the six *HLA* genes, testing accuracies and call rates are shown for the four genotyping arrays (colored bars). Data from CT = 0 (blue), CT = 0.5 (red), and CT = 0.9 (gray) are shown. Panels represent data without imputed SNPs (A) and with imputed SNPs (B). Click here for file

Additional file 6 **The percentage of overlapping selected genotypes between the different genotyping platforms.** For each of the six *HLA* genes (colored bars), the percentage of overlapping SNPs from different pairs of arrays is shown. Data without imputed SNPs (A) and with imputed SNPs (B) are shown. Click here for file

Additional file 7 **List of the selected SNPs in the final *HLA *** **prediction models.** Click here for file

Additional file 8 **List of the SNP alleles that predict *HLA *** **allele types.** Click here for file

Additional file 9 **Testing accuracies with different CV settings.** For each of the six *HLA* genes, testing accuracies associated with 10-fold CV (blue), 4-fold CV (red), and 2-fold CV (green) are shown (for CT = 0). Click here for file
