# Supplementary material for: Predicting HLA genotypes using unphased and flanking single-nucleotide polymorphisms in Han Chinese population
Source: BMC Genomics. 2014 Jan 29;15:81. doi: 10.1186/1471-2164-15-81 (PMC3909910; doi:10.1186/1471-2164-15-81)
Supplement: Additional file 1 — Overlapping SNPs between the HapMap Project and three genotyping platforms within the extended MHC region. [file 1471-2164-15-81-S1.pdf]

Additional file 5. Overlapping SNPs between the HapMap Project and three genotyping platforms within the extended MHC region[1].

Overlap	HapMap[2]	Affy 5.0	Affy 6.0	Illumina 550K
**HapMap**	1[3]	0.792319	0.822969	0.887571
	(6,749)[4]	(1,114)	(1,813)	(1,721)
**Affy 5.0**	0.165061	1	0.595098	0.139247
		(1,406)	(1,311)	(270)
**Affy 6.0**	0.268632	0.932432	1	0.232078
			(2,203)	(450)
**Illumina 550K**	0.255001	0.192034	0.204267	1
				(1,939)

[1]The range of the extended MHC region is chr6 28,799,220–34,204,868 [31].
[2]HapMap SNPs from the Chinese Han Beijing population.
[3]The proportion of the overlapping SNPs between the two genotyping platforms.
[4]The number of SNPs in each genotyping platform.
