# Supplementary material for: Predicting HLA genotypes using unphased and flanking single-nucleotide polymorphisms in Han Chinese population
Source: BMC Genomics. 2014 Jan 29;15:81. doi: 10.1186/1471-2164-15-81 (PMC3909910; doi:10.1186/1471-2164-15-81)
Supplement: Additional file 2 — List of the HLA alleles and allele frequencies in our Han Chinese data (n = 214). [file 1471-2164-15-81-S2.pdf]

Additional file 8. List of the *HLA* alleles and allele frequencies in our Han Chinese data (n=214)

#	*HLA-A*		*HLA-B*		*HLA-C*		*HLA-DPB1*		*HLA-DQB1*		*HLA-DRB1*	
	allele	allele frequency (%)	allele	allele frequency (%)	allele	allele frequency (%)	allele	allele frequency (%)	allele	allele frequency (%)	allele	allele frequency (%)
1	01:01	0.23	07:02	0.47	01:02	20.09	01:01	0.23	02:01	10.33	01:01	0.7
2	02:01	9.15	07:05	0.23	01:03	0.23	01:02	0.47	02:02	2.11	03:01	10.56
3	02:03	6.81	08:01	0.23	02:02	0.23	02:01	17.76	03:01	21.36	04:01	0.7
4	02:06	3.52	13:01	3.5	03:02	12.62	02:02	7.71	03:02	7.75	04:03	3.52
5	02:07	10.09	13:02	1.64	03:03	2.57	03:01:05:02	3.97	03:03	16.67	04:04	0.7
6	03:01	0.7	15:01	2.57	03:04	7.71	04:01	10.05	04:01	6.81	04:05	7.04
7	11:01:02	32.16	15:02	5.84	03:04:03	0.7	04:02	1.4	04:02	1.64	04:06	3.05
8	24:02	15.73	15:03	0.47	04:01	5.37	05:01	40.89	05:01	3.05	04:07	0.23
9	24:03	0.23	15:08	0.23	04:03	2.57	09:01	1.17	05:02	9.15	04:10	0.47
10	24:07	0.23	15:11	0.47	06:02	2.34	09:02	2.1	05:03	4.93	07:01	2.58
11	24:10	0.47	15:18	1.17	07:01	0.23	13:01	5.37	06:01	11.03	08:02	0.7
12	26:01	2.11	15:25	0.7	07:02	21.96	14:01	2.8	06:02	3.05	08:03	9.15
13	26:02	0.23	15:27	1.64	07:04	1.4	17:01	1.4	06:09	1.88	08:09	0.47
14	29:01	0.23	15:32	0.23	08:01	8.64	19:01	1.64	06:10	0.23	09:01	15.96
15	30:01	1.17	27:04	2.34	12:02	4.21	21:01	2.57			10:01	0.94
16	31:01	1.64	27:04:06	0.23	12:03	1.17	31:01	0.23			11:01	7.04
17	32:01	0.23	27:05	0.23	14:02	3.5	48:01	0.23			11:06	0.23
18	33:03	14.32	27:06	0.23	15:02	4.21					12:01	3.05
19	34:01	0.23	35:01	2.1	15:05	0.23					12:02	9.62
20	69:01	0.23	35:02	0.23							13:01	0.23
21	74:01	0.23	35:05	0.23							13:02	1.64
22			37:01	0.47							13:12	1.17
23			38:02	3.5							14:01	3.29
24			39:01	2.34							14:03	0.47
25			40:01	20.09							14:04	0.47
26			40:02	1.64							14:05	2.35
27			40:03	0.23							14:10	0.23
28			40:06	1.17							14:18	0.47
29			44:03	0.7							15:01	5.63
30			46:01	12.62							15:02	2.35
31			48:01	1.4							16:02	4.93
32			51:01	4.44								

33			51:02	1.64								
34			52:01	1.17								
35			54:01	3.97								
36			55:02	3.74								
37			55:04	0.23								
38			56:01	0.93								
39			56:03	0.23								
40			56:04	0.47								
41			57:01	0.23								
42			58:01	12.85								
43			59:01	0.23								
44			67:01	0.7								
