# Supplementary figures and images for: Predicting HLA genotypes using unphased and flanking single-nucleotide polymorphisms in Han Chinese population
Source: BMC Genomics. 2014 Jan 29;15:81. doi: 10.1186/1471-2164-15-81 (PMC3909910; doi:10.1186/1471-2164-15-81)

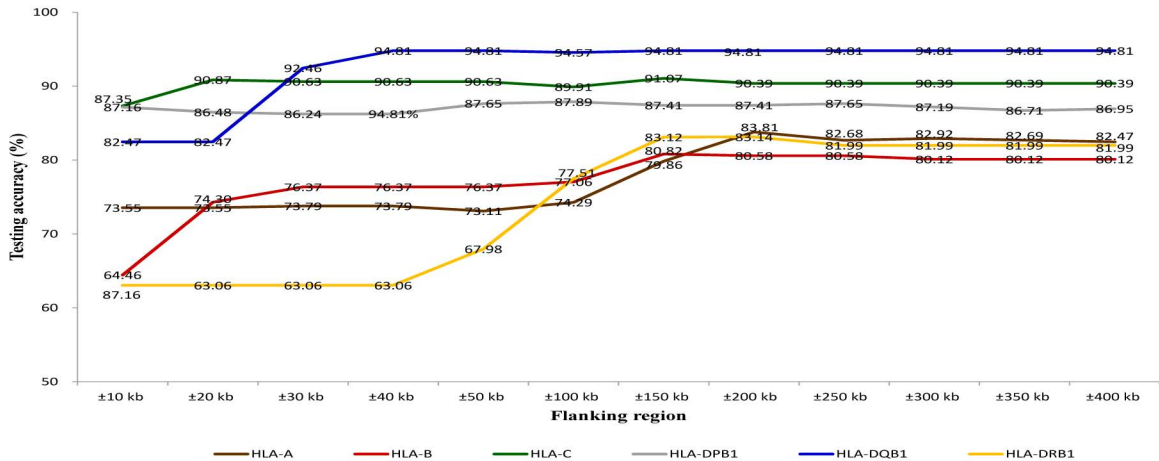

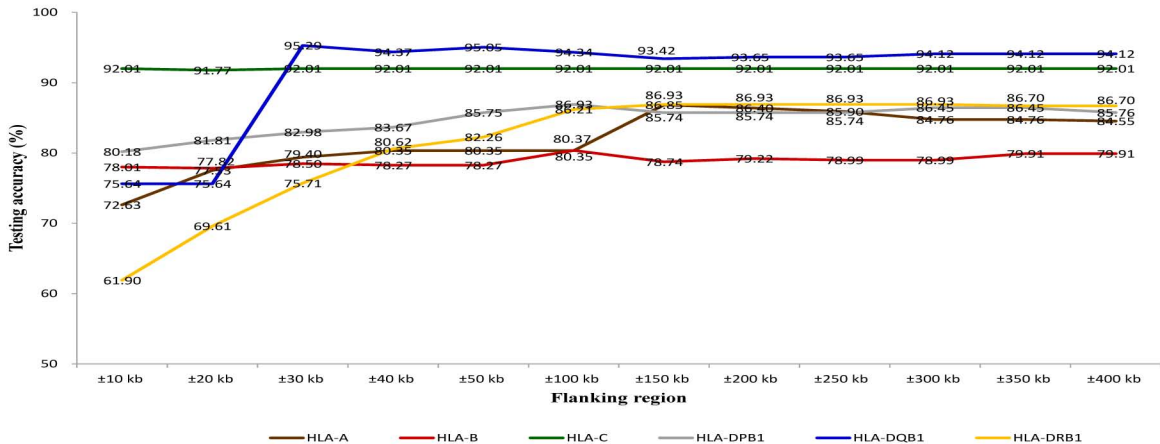

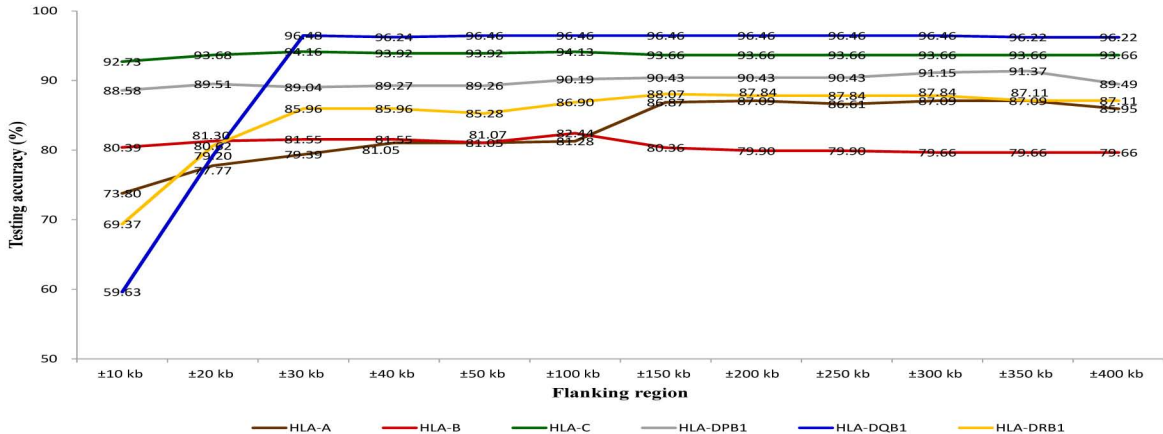

Supplement: Additional file 3 — Testing accuracies associated with different flanking-region sizes. For each of the six HLA genes (colored lines), testing accuracies for various flanking-region sizes are shown. Data using the Affy 5.0 (A), Illumina 550 K (B), and Union (C) chip data sets are shown. [Union refers to a union of data from the three platforms (Affy 5.0, Affy 6.0, and Illumina 550 K)]. [file 1471-2164-15-81-S3.pdf]

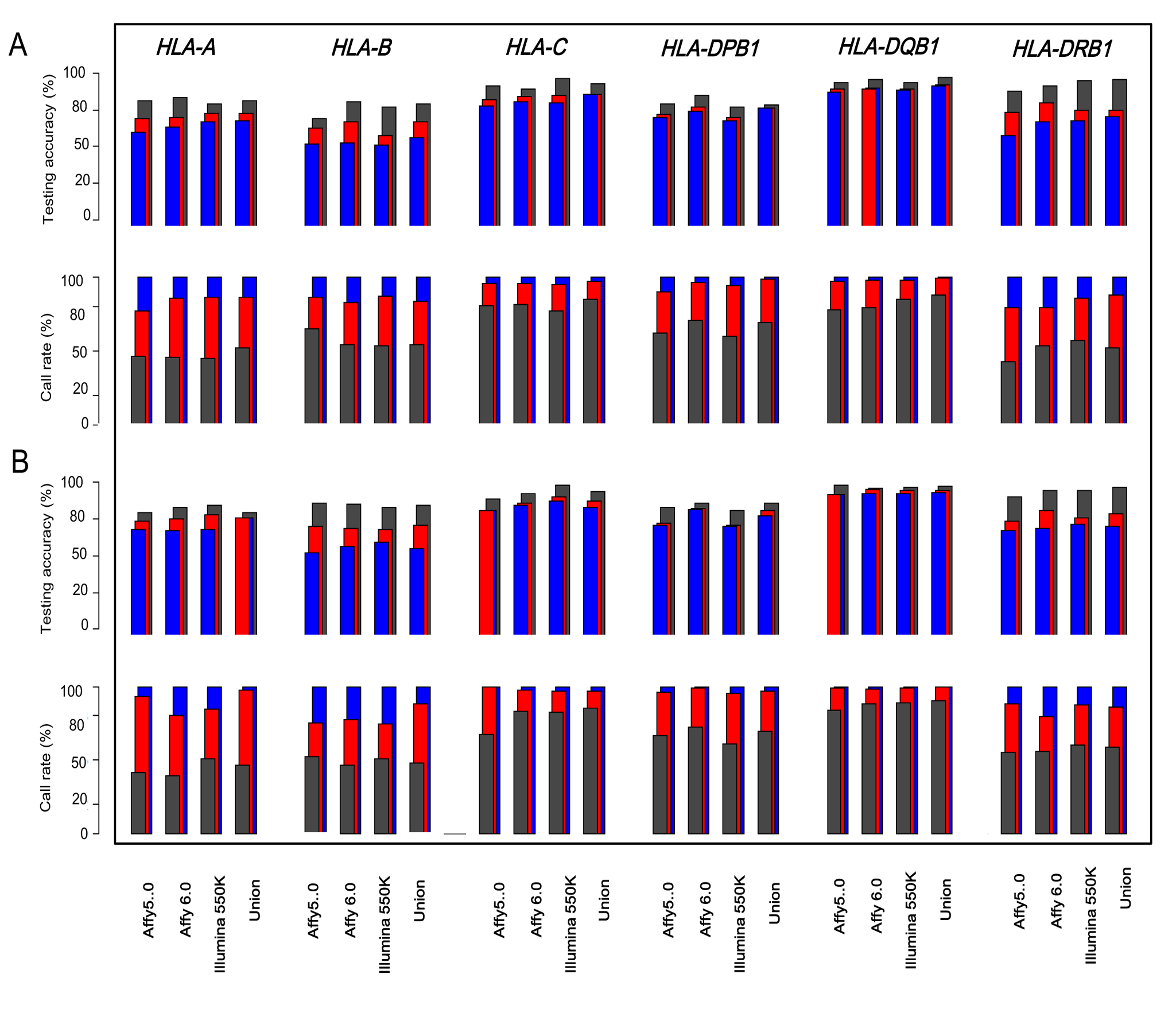

Supplement: Additional file 5 — Testing accuracies and call rates from optimized models for each platform at different CT settings. For each of the six HLA genes, testing accuracies and call rates are shown for the four genotyping arrays (colored bars). Data from CT = 0 (blue), CT = 0.5 (red), and CT = 0.9 (gray) are shown. Panels represent data without imputed SNPs (A) and with imputed SNPs (B). [file 1471-2164-15-81-S5.tiff]

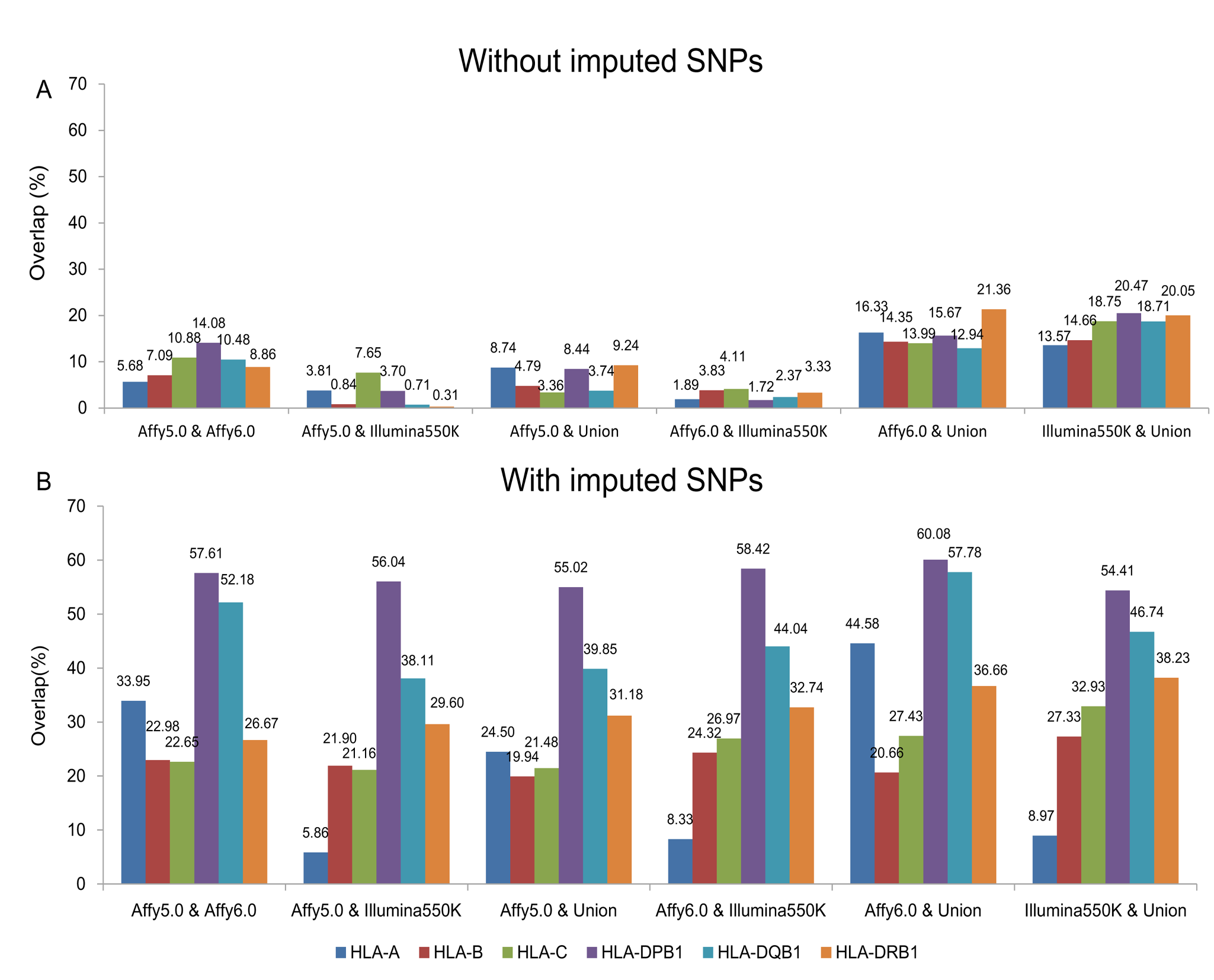

Supplement: Additional file 6 — The percentage of overlapping selected genotypes between the different genotyping platforms. For each of the six HLA genes (colored bars), the percentage of overlapping SNPs from different pairs of arrays is shown. Data without imputed SNPs (A) and with imputed SNPs (B) are shown. [file 1471-2164-15-81-S6.tiff]

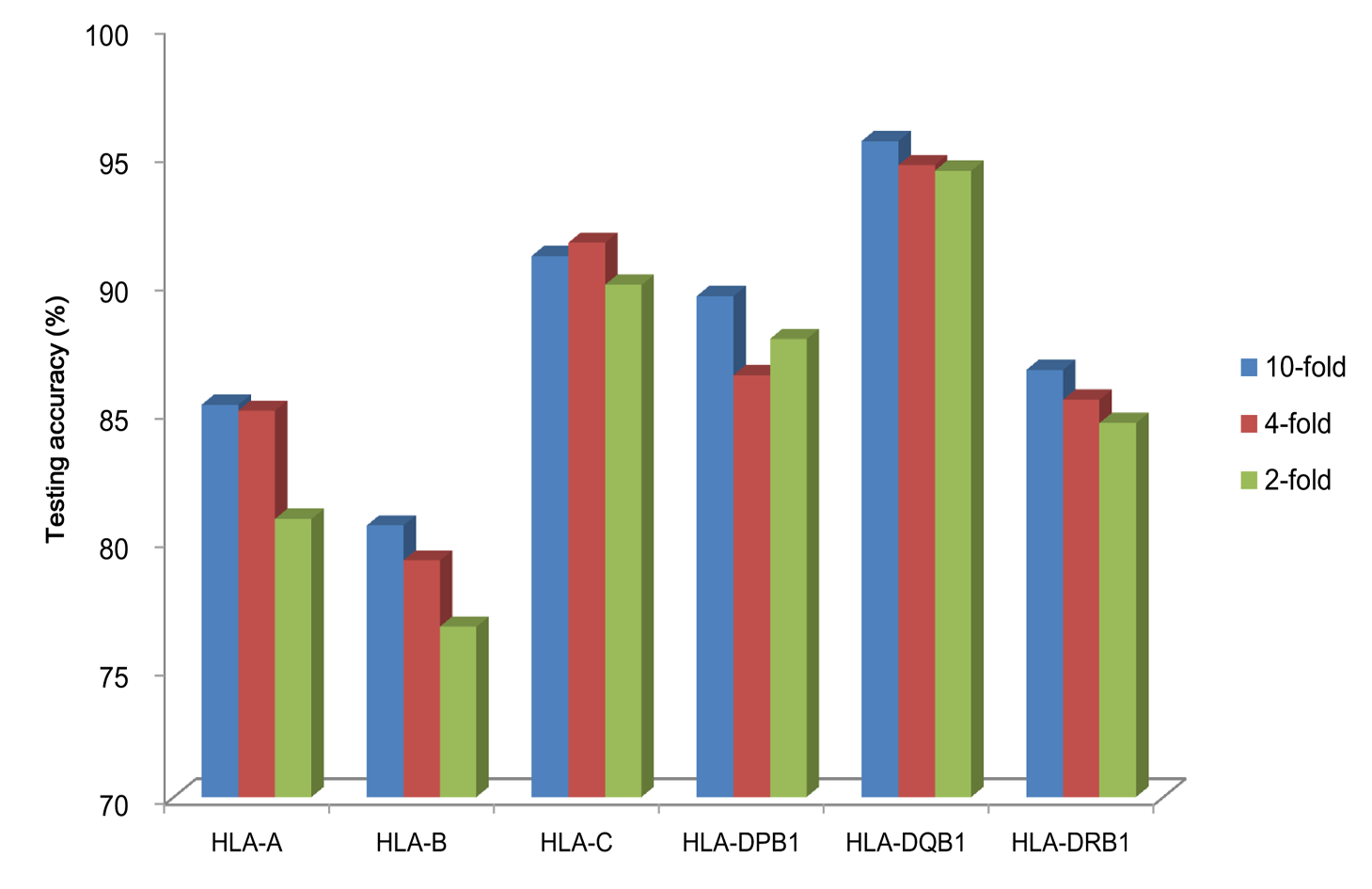

Supplement: Additional file 9 — Testing accuracies with different CV settings. For each of the six HLA genes, testing accuracies associated with 10-fold CV (blue), 4-fold CV (red), and 2-fold CV (green) are shown (for CT = 0). [file 1471-2164-15-81-S9.tiff]
