# Supplementary material for: Predicting HLA genotypes using unphased and flanking single-nucleotide polymorphisms in Han Chinese population
Source: BMC Genomics. 2014 Jan 29;15:81. doi: 10.1186/1471-2164-15-81 (PMC3909910; doi:10.1186/1471-2164-15-81)
Supplement: Additional file 4 — Kappa coefficients of observed or imputed SNP genotypes within the MHC region. [file 1471-2164-15-81-S4.pdf]

Additional file 6. Kappa coefficients of observed or imputed SNP genotypes within the MHC region.

**Scenarios**	**Platform**	**N**[1]	**Kappa coefficient**
Observed genotypes	Affy 5.0 and Affy 6.0	1021	0.9926465
	Affy 5.0 and Illumina 550K	229	0.994711
	Affy 6.0 and Illumina 550K	411	0.996092
Observed and imputed genotypes	Affy 5.0 and Affy 6.0	5238	0.9672119
	Affy 5.0 and Illumina 550K	5227	0.9587444
	Affy 6.0 and Illumina 550K	5316	0.9761685
Observed vs. imputed genotypes[2]	Affy 5.0	877	0.992273
	Affy 6.0	1502	0.9933604
	Illumina 550K	1562	0.9965305

[1]Number of genotypes

[2]Genotypic comparisons between observed and imputed genotypes.
