# Supplementary material for: Predicting HLA genotypes using unphased and flanking single-nucleotide polymorphisms in Han Chinese population
Source: BMC Genomics. 2014 Jan 29;15:81. doi: 10.1186/1471-2164-15-81 (PMC3909910; doi:10.1186/1471-2164-15-81)
Supplement: Additional file 7 — List of the selected SNPs in the final HLA prediction models. [file 1471-2164-15-81-S7.pdf]

Additional file 7. List of the selected SNPs in the final *HLA* prediction models

	Without imputation							
	Affy5.0		Affy6.0		Illumina550K		Union	
	rs	position (build 36.3)	rs	position (build 36.3)	rs	position (build 36.3)	rs	position (build 36.3)
HLA-A	rs1633085	29823976	rs4122198	29853572	rs3094165	29941520	rs2523409	29883641
	rs2254071	29914041	rs16895757	29870157	rs9258883	29945167	rs1611133	29917361
	rs407238	29914880	rs1632973	29885150	rs3132714	29948627	rs3115628	29926000
	rs9258881	29945121	rs9357086	29886008	rs1611493	29994143	rs2517859	29930550
	rs2975046	30023128	rs11759549	29925902	rs2524005	30007656	rs1611732	29938987
	rs2735096	30023730	rs3115628	29926000	rs2860580	30014670	rs2523998	30012844
	rs417162	30024484	rs3094165	29941520	rs12665039	30032758	rs2860580	30014670
	rs9260954	30067914	rs2734925	29989326	rs6457109	30041240	rs12202296	30016448
	rs6917477	30133963	rs2517755	30004638	rs3869062	30042870	rs2248153	30022594
	rs6457144	30171347	rs2256919	30048729	rs3893464	30043229	rs2975046	30023128
	rs9261394	30172541	rs11756025	30061421	rs5009448	30048467	rs6457109	30041240
	rs2523990	30185208	rs7382061	30155944	rs2571375	30053249	rs5009448	30048467
			rs6457144	30171347	rs7758512	30078568	rs9260932	30065781
			rs2517646	30230554	rs9261394	30172541	rs6457144	30171347
			rs7744914	30456226				
HLA-B	rs3130944	31304650	rs11966319	31337657	rs9264868	31379580	rs3094691	31382672
	rs3130532	31316432	rs2853948	31353552	rs9264942	31382359	rs7453967	31422222
	rs3130534	31317024	rs6906846	31353715	rs3094691	31382672	rs4394274	31426143
	rs3134762	31318845	rs9378228	31354350	rs2156875	31425326	rs4394275	31426156
	rs16899207	31374366	rs2524051	31363479	rs2523619	31426123	rs2596509	31426392
	rs2524089	31374501	rs9366778	31377152	rs2442719	31428517	rs2596501	31429190
	rs9366778	31377152	rs16867947	31388802	rs2596501	31429190	rs1058026	31429664
	rs2524166	31396506	rs4394274	31426143	rs2523589	31435313	rs2523591	31434939
	rs9295984	31425676	rs4394275	31426156	rs2523554	31439808	rs2523589	31435313
	rs4394275	31426156	rs2523591	31434939	rs2844573	31443433	rs2523554	31439808
	rs9378249	31435680	rs9501572	31441799	rs9266395	31443545	rs2523545	31441478
	rs2523534	31444328	rs7761068	31441918	rs9266440	31445794	rs9501572	31441799
	rs9266406	31444397	rs2523535	31444229	rs9295986	31446507	rs2844575	31442924
	rs2844558	31448412	rs9266406	31444397	rs2442749	31460019	rs9266395	31443545
	rs5022119	31451841	rs5006724	31445829	rs2596560	31463297	rs9266406	31444397

	rs3099848	31459421	rs13198903	31452136	rs3128982	31525170	rs5006725	31445851
	rs4081552	31461668	rs9266669	31456056	rs2284178	31540104	rs9295986	31446507
	rs2848716	31495946	rs9266689	31456559	rs7758090	31546773	rs6933050	31451611
	rs2596454	31544291	rs3099849	31459394			rs4959068	31451823
	rs2248462	31554775	rs2442749	31460019			rs5022119	31451841
			rs1051796	31487752			rs13198903	31452136
			rs2596464	31520940			rs9266689	31456559
			rs3099836	31526049			rs2251396	31472686
			rs3131622	31528479			rs1051796	31487752
							rs3094584	31491827
							rs9765960	31520979
							rs3128982	31525170
HLA-C	rs2073724	31237686	rs3130712	31317489	rs2516049	32678378	rs9263957	31292154
	rs3130713	31313596	rs28480108	31318511	rs2858870	32680229	rs9263969	31294513
	rs3130531	31314595	rs3134762	31318845	rs660895	32685358	rs3134762	31318845
	rs3095250	31316319	rs11966319	31337657	rs532098	32686030	rs11966319	31337657
	rs3130532	31316432	rs9264523	31341737	rs3129763	32698903	rs2248880	31341489
	rs3130534	31317024	rs3132488	31350674	rs1063355	32735692	rs9264532	31342360
	rs2844615	31350938	rs3134745	31350741	rs9275141	32759095	rs2524099	31344030
	rs6906846	31353715	rs3130693	31350838	rs9275184	32762692	rs2074488	31348410
	rs2524067	31353800	rs3132486	31351149	rs7774434	32765556	rs2395471	31348671
	rs7382297	31355046	rs2853948	31353552	rs7775228	32766057	rs5010528	31349011
	rs2394963	31359441	rs6906846	31353715	rs9275224	32767856	rs13207315	31349106
	rs2524095	31374096	rs9378228	31354350			rs3132488	31350674
	rs16899203	31374314	rs6457372	31355100			rs3130693	31350838
	rs9366778	31377152	rs2394963	31359441			rs9391714	31353059
	rs9295970	31377501	rs2524057	31359874			rs4386816	31355114
	rs2523534	31444328	rs12191877	31360904			rs2524057	31359874
			rs9366776	31364609			rs16899205	31374340
							rs9295970	31377501
HLA-DPB1	rs3128955	33129170	rs9296068	33096673	rs206769	33069082	rs2116264	33092766
	rs3130588	33130040	rs9277183	33131348	rs6920606	33105652	rs423639	33095752
	rs9277194	33131872	rs3135402	33132632	rs375912	33124706	rs3097669	33131770

	rs9348904	33148813	rs9348904	33148813	rs1431399	33149012	rs987870	33150858
	rs9296073	33150529	rs2856830	33149712	rs987870	33150858	rs1431402	33154893
	rs2856816	33153478	rs9296073	33150529	rs3135021	33153536	rs1431403	33155009
	rs3135021	33153536	rs2071350	33151504	rs9277535	33162839	rs9277378	33158257
	rs1431403	33155009	rs1431402	33154893	rs9277554	33163516	rs9277535	33162839
	rs3128963	33163758	rs1431403	33155009	rs10484569	33166930	rs9277550	33163465
	rs3117229	33164047	rs9277550	33163465	rs2281390	33167647	rs9277554	33163516
	rs7763822	33168406	rs3128963	33163758	rs3128917	33167974	rs9277565	33164875
	rs2295120	33168747	rs3117229	33164047	rs2281388	33168096	rs2281390	33167647
	rs3117242	33177871	rs9277567	33164991	rs3130215	33182941	rs2281388	33168096
	rs6937034	33187744	rs3128918	33169076	rs2269346	33266876	rs3130215	33182941
	rs1003979	33222149	rs6937034	33187744			rs6937034	33187744
							rs6937061	33187790
							rs2395357	33208984
HLA-DQB1	rs9269186	32556394	rs2647073	32681992	rs2516049	32678378	rs17533090	32698700
	rs9270986	32682038	rs502055	32686981	rs2858870	32680229	rs9272219	32710247
	rs615672	32682149	rs3129768	32703061	rs660895	32685358	rs17211510	32710408
	rs3129768	32703061	rs9272535	32714734	rs532098	32686030	rs41269947	32716055
	rs9272219	32710247	rs9272723	32717405	rs3129763	32698903	rs34485459	32731255
	rs9272346	32712350	rs34485459	32731255	rs1063355	32735692	rs1063355	32735692
	rs6908943	32743274	rs3129716	32765414	rs9275141	32759095	rs9275141	32759095
	rs9275134	32758590	rs7775228	32766057	rs9275184	32762692	rs3129716	32765414

	rs9469220	32766288	rs9469219	32766189	rs7774434	32765556	rs7774434	32765556
	rs6457617	32771829	rs5000634	32771542	rs7775228	32766057	rs9405119	32765659
	rs2647046	32776314	rs6457617	32771829	rs9275224	32767856	rs9469219	32766189
	rs2858308	32777978	rs9275418	32778222			rs9469220	32766288
	rs9275418	32778222					rs9275224	32767856
HLA-DRB1	rs9268831	32535726	rs9268877	32539125	rs9405098	32487714	rs2395175	32513004
	rs9268861	32537872	rs4410767	32556107	rs3129871	32514320	rs9405035	32515046
	rs7747521	32539083	rs7749092	32557028	rs13209234	32523953	rs9268831	32535726
	rs9268877	32539125	rs17210980	32665279	rs9268832	32535767	rs6903608	32536263
	rs9269186	32556394	rs2647073	32681992	rs6903608	32536263	rs9268877	32539125
	rs2027852	32652330	rs615672	32682149	rs602875	32681607	rs9269186	32556394
	rs615672	32682149	rs674313	32686060	rs660895	32685358	rs7749092	32557028
	rs3129768	32703061	rs502771	32686948	rs9271366	32694832	rs2027852	32652330
	rs9272219	32710247	rs3997872	32688595	rs3129763	32698903	rs17210980	32665279
	rs9272346	32712350	rs9271367	32694886	rs17211510	32710408	rs2516049	32678378
	rs9275134	32758590	rs9271720	32701485	rs2187668	32713862	rs615672	32682149
	rs7775228	32766057	rs2187668	32713862	rs9275141	32759095	rs660895	32685358
	rs9469220	32766288	rs34485459	32731255	rs9275184	32762692	rs674313	32686060
	rs6457617	32771829	rs3129716	32765414	rs9275383	32776824	rs502771	32686948
	rs2647046	32776314	rs9405119	32765659	rs2856717	32778286	rs3997872	32688595
	rs2858308	32777978			rs2858305	32778442	rs9271366	32694832
					rs13192471	32779081	rs2187668	32713862
					rs3104405	32790286	rs34485459	32731255
							rs9275141	32759095
							rs7755224	32760295
							rs3129716	32765414
							rs3104404	32790152
