# Supplementary material for: Predicting HLA genotypes using unphased and flanking single-nucleotide polymorphisms in Han Chinese population
Source: BMC Genomics. 2014 Jan 29;15:81. doi: 10.1186/1471-2164-15-81 (PMC3909910; doi:10.1186/1471-2164-15-81)
Supplement: Additional file 8 — List of the SNP alleles that predict HLA allele types. [file 1471-2164-15-81-S8.pdf]

Additional file 9. List of the SNP alleles that predict *HLA* allele types

Without impute, *HLA-A*, Affy 5.0

T	C	G	A	A	A	T	C	G	T	A	G	201
T	C	G	A	A	A	T	C	G	T	A	A	201
C	C	G	A	A	A	T	C	G	T	A	G	201
T	T	G	A	A	A	T	C	G	T	A	G	201
T	C	G	A	A	A	T	C	G	A	G	A	201
T	C	G	A	A	A	T	C	G	T	A	G	203
C	C	G	A	A	A	T	C	G	A	G	A	203
C	T	G	G	G	A	T	C	G	A	G	G	203
T	T	G	A	A	A	T	C	G	A	G	A	203
T	T	G	A	A	A	T	C	G	T	A	G	203
T	C	G	A	A	A	T	C	G	T	A	A	203
T	C	G	A	A	A	T	C	G	T	A	A	206
T	C	G	A	A	A	T	C	G	A	G	A	207
T	C	G	A	A	A	T	C	G	T	A	G	207
T	C	G	A	A	A	T	C	G	A	G	G	207
C	C	G	A	A	G	C	C	G	T	A	G	207
C	C	C	A	A	G	T	G	C	A	G	A	207
T	C	G	A	A	A	T	C	G	T	G	A	207
T	T	G	A	A	A	T	C	G	T	A	G	207
T	T	G	A	A	A	T	C	G	A	G	A	207
T	C	C	G	A	G	C	C	G	T	A	A	301

C	T	G	G	G	A	T	C	G	A	G	A	2402
C	T	G	G	G	A	T	C	G	T	A	G	2402
C	T	G	G	G	A	T	C	G	A	G	G	2402
T	T	G	G	G	A	T	C	G	A	G	A	2402
C	T	G	G	G	A	T	G	C	A	G	A	2402
C	T	G	G	G	A	T	C	G	T	G	A	2403
C	T	G	G	G	A	T	C	G	T	A	G	2407
C	T	G	G	G	A	T	C	G	A	G	A	2410
C	C	C	G	A	G	T	G	C	A	G	A	2601
C	C	C	A	A	G	T	G	C	A	G	G	2601
C	C	C	A	A	G	T	G	C	A	G	A	2601
C	T	G	G	A	G	C	C	G	T	A	G	2601
C	C	C	G	A	G	T	G	C	A	G	G	2601
C	C	C	G	A	A	T	G	C	A	G	A	2602
C	T	G	G	A	G	C	C	G	A	G	G	2901
C	C	C	A	A	G	C	C	C	A	G	A	3001
C	C	C	A	A	A	C	C	C	A	G	A	3001
C	C	C	G	A	G	T	C	G	A	G	G	3101
C	T	G	A	G	A	T	C	G	A	G	A	3201
C	T	G	G	A	G	T	C	C	A	G	A	3303
C	T	G	G	A	A	T	C	C	A	G	A	3303
C	T	G	G	A	G	T	C	C	T	G	A	3303
T	C	C	A	A	G	T	C	C	A	G	A	3303

T	T	G	A	A	G	T	C	G	A	G	A	3401
C	T	G	A	G	A	T	C	G	A	G	A	7401

Without impute, *HLA-A*, Affy 6.0

T	C	C	C	G	T	G	C	T	A	C	C	T	T	G	201
T	C	C	C	G	T	G	C	T	A	C	C	A	T	G	201
T	T	C	G	G	T	G	C	T	A	C	C	T	T	G	201
T	C	C	G	G	T	G	C	T	A	C	T	T	C	G	201
C	C	C	C	G	T	G	C	T	A	C	C	T	T	G	201
T	C	C	C	G	T	G	C	T	A	C	C	T	T	G	203
T	C	C	C	G	T	G	C	T	A	C	T	A	T	G	203
C	C	C	G	G	T	A	C	C	A	C	T	A	C	G	203
T	C	C	G	G	T	G	C	T	A	C	C	T	T	G	206
T	C	C	G	G	T	G	C	T	A	C	T	T	T	G	206
T	C	C	C	G	T	G	C	T	A	C	C	T	T	G	206
T	C	C	G	G	T	G	C	T	A	C	C	T	C	G	206
T	C	C	C	G	T	G	C	T	A	C	C	A	T	G	207
T	C	C	C	G	T	G	C	T	A	C	C	T	T	G	207
C	C	C	G	C	C	G	G	C	C	T	T	A	T	A	207
T	C	C	G	G	T	G	G	C	C	T	T	A	C	G	207
T	C	C	G	G	C	A	G	C	C	C	C	T	C	G	301
C	C	C	G	G	T	A	C	C	A	C	T	A	T	G	2402
C	C	C	G	G	T	A	C	T	A	C	T	A	T	G	2402

C	C	C	G	G	T	A	C	C	A	C	C	T	T	G	2402
C	C	C	G	G	T	A	C	T	A	C	C	T	T	G	2402
C	C	C	G	G	T	A	G	C	A	C	C	T	T	G	2402
T	T	C	G	G	T	A	C	C	A	C	T	A	T	G	2402
C	C	C	G	G	T	A	C	T	A	C	T	A	C	G	2402
T	T	C	G	G	T	A	C	C	A	C	C	T	C	G	2402
C	C	C	G	G	T	A	C	C	A	C	C	T	C	G	2402
C	C	C	G	G	T	A	C	T	A	C	C	A	T	G	2402
C	C	C	G	G	T	A	G	C	A	C	T	A	T	G	2403
C	C	C	G	G	T	A	C	C	A	C	T	A	T	G	2407
C	C	C	G	G	T	A	C	C	A	C	T	A	T	G	2410
T	C	T	G	G	C	G	C	C	C	C	T	A	C	G	2601
T	C	T	G	G	C	G	C	C	C	C	T	A	T	G	2601
T	T	C	G	G	T	G	C	C	C	C	C	T	T	G	2601
T	C	T	G	G	C	G	C	C	C	C	C	A	T	G	2601
T	C	T	G	G	T	G	C	C	C	C	T	A	C	G	2602
T	T	C	G	G	T	G	C	C	C	C	T	A	T	G	2901
T	C	T	G	G	C	A	C	C	A	T	T	A	C	G	3001
T	C	T	G	G	T	G	G	C	C	T	T	A	C	G	3101
C	C	C	G	G	C	G	G	C	C	C	T	A	T	G	3201
T	C	C	G	C	C	G	G	C	C	T	T	A	T	A	3303
C	C	C	G	G	C	G	G	C	C	T	T	A	C	G	3303
T	C	C	G	C	C	G	C	C	C	T	T	A	C	A	3303

T	C	T	G	G	T	G	G	T	A	T	T	A	T	G	3401
T	C	C	G	G	C	G	G	C	A	C	T	A	T	G	7401

Without impute, *HLA-A*, Illumina 550K

G	T	C	G	G	G	T	T	A	G	C	A	T	A	201
G	T	C	G	G	G	T	T	A	A	C	A	T	G	201
G	T	C	G	G	G	T	C	A	G	C	A	T	A	201
G	T	C	G	G	G	T	T	A	G	C	A	T	A	203
G	T	C	G	G	G	T	T	A	G	T	A	T	A	203
G	T	C	G	G	G	T	T	A	G	C	A	T	G	203
A	T	T	G	G	G	T	T	A	A	C	A	T	G	203
G	T	C	G	G	G	T	C	A	G	C	A	T	A	206
G	T	C	G	G	G	T	T	A	G	C	A	T	G	207
G	T	C	G	G	G	T	T	A	G	C	A	T	A	207
G	T	C	T	G	G	T	T	A	A	T	A	G	G	207
G	T	C	T	A	G	C	T	A	A	C	A	G	G	207
A	C	T	G	G	A	T	T	A	G	T	A	T	A	301
A	C	T	G	G	A	T	T	A	G	T	G	T	A	301
A	T	T	G	G	G	T	T	A	A	C	A	T	G	2402
A	T	T	T	G	G	T	T	A	A	C	A	T	G	2402
A	T	T	G	G	G	T	T	A	A	C	A	T	A	2402
A	T	T	G	G	G	T	T	A	G	C	A	T	A	2402
A	T	T	T	G	G	T	T	A	A	C	A	T	A	2402

A	T	T	G	G	G	T	T	A	G	C	A	T	G	2402
A	T	T	G	G	G	T	T	A	G	T	A	G	G	2402
A	T	T	T	G	G	T	T	A	A	C	A	T	G	2403
A	T	T	G	G	G	T	T	A	A	C	A	T	G	2407
A	T	T	T	G	G	T	T	A	A	C	A	T	G	2410
A	T	T	G	G	G	T	T	A	A	C	A	T	G	2410
G	T	C	T	G	G	T	T	A	G	T	A	G	G	2601
G	C	T	G	G	A	T	T	G	A	T	A	T	A	2601
G	T	C	T	G	G	T	T	A	G	T	A	G	G	2602
G	C	T	G	G	A	T	T	G	A	T	A	T	G	2901
A	T	C	G	G	A	T	C	A	G	C	A	G	G	3001
G	C	T	T	A	G	C	T	A	A	C	G	G	G	3101
G	T	C	T	G	G	T	T	A	A	T	A	T	G	3201
G	C	T	T	A	G	C	T	A	A	C	A	G	G	3303
G	T	C	T	A	G	C	T	A	A	C	A	G	G	3303
G	T	C	G	G	A	C	T	A	A	C	A	G	G	3401
G	T	C	T	G	G	T	T	A	A	C	A	T	G	7401

Without impute, *HLA-A*, Union

G	C	T	G	C	T	G	C	A	A	T	C	T	T	201
A	C	T	G	C	T	G	C	A	A	T	C	T	T	201
G	C	T	G	C	T	G	C	A	A	C	C	T	T	201
G	C	T	G	C	T	G	C	A	A	T	C	T	T	203

G	C	T	G	C	T	G	C	A	A	T	T	T	T	203
G	C	T	G	C	T	G	C	A	A	T	C	T	A	203
A	C	T	G	T	T	G	T	A	G	T	C	T	A	203
G	C	T	G	C	T	G	C	A	A	C	C	T	T	206
G	C	T	G	C	T	G	C	A	A	T	C	T	A	207
A	C	T	G	C	T	G	C	A	A	T	C	T	A	207
G	C	T	G	C	T	G	C	A	A	T	C	T	T	207
A	C	T	G	C	T	A	T	A	A	T	T	T	T	207
G	T	T	G	C	G	G	T	C	G	T	T	C	A	207
A	C	C	G	C	T	A	T	A	A	T	T	T	T	301
A	C	T	G	T	T	G	T	A	G	T	C	T	A	2402
A	C	T	G	T	T	G	T	A	G	T	C	T	T	2402
A	C	T	G	T	T	G	T	A	G	T	C	T	A	2403
A	C	T	G	T	T	G	T	A	G	T	C	T	T	2407
G	C	T	G	T	T	G	T	A	G	T	C	T	A	2410
A	T	C	G	C	G	G	T	C	A	T	T	C	A	2601
A	T	C	G	C	G	G	T	C	A	T	T	T	A	2601
A	C	T	G	T	T	A	T	A	A	T	T	T	A	2601
A	C	T	G	T	T	A	T	A	A	T	T	T	T	2601
A	T	T	G	C	G	G	T	C	A	T	T	C	A	2602
A	C	T	G	T	T	A	T	A	A	T	T	T	A	2901
A	C	T	G	T	T	A	T	A	A	T	T	T	T	2901
A	T	C	G	T	T	A	T	A	A	C	C	T	A	3001

A	T	T	G	C	G	G	T	C	A	T	C	T	A	3101
A	C	C	G	T	G	G	T	C	G	T	T	T	A	3201
A	C	C	G	C	G	G	T	C	A	T	C	T	A	3303
A	C	T	A	T	T	A	C	C	A	T	C	T	A	3401
A	C	C	G	T	G	G	T	C	G	T	C	T	A	7401

Without impute, *HLA-B*, Affy 5.0

C	A	G	T	A	G	G	T	G	G	T	G	G	C	T	A	G	G	A	A	702
C	A	G	T	A	G	G	C	G	A	T	G	G	T	A	T	G	G	G	G	705
C	G	A	T	G	T	A	C	G	A	T	A	G	C	T	T	A	G	G	G	705
C	A	G	T	A	G	G	C	G	G	T	A	G	C	T	T	A	G	G	G	801
C	A	G	T	A	G	G	C	G	G	T	G	G	T	A	T	G	G	G	G	801
C	G	A	T	A	G	G	C	G	G	T	G	G	T	A	A	G	G	G	G	1301
C	A	G	T	A	G	G	T	G	A	T	G	G	T	A	A	G	G	G	G	1301
G	G	A	C	A	T	A	T	A	G	T	G	G	T	A	A	G	G	G	G	1301
C	G	A	T	A	G	G	T	G	G	T	G	G	T	A	A	G	G	G	G	1301
G	G	A	C	A	G	G	T	G	G	T	A	G	C	T	A	G	G	G	A	1302
G	G	A	C	A	G	G	C	G	G	T	A	G	C	T	A	G	G	G	A	1302
G	G	A	C	A	G	G	T	G	G	T	G	G	C	T	A	G	G	G	G	1302
C	G	G	T	G	T	A	C	G	A	G	G	A	T	A	A	G	C	G	G	1501
C	G	G	T	A	G	G	T	G	G	G	G	A	T	A	A	G	C	G	G	1501
G	G	G	T	G	T	A	C	G	A	G	G	A	T	A	A	G	C	G	G	1501
G	G	A	C	A	T	A	T	A	G	G	G	A	T	A	A	G	C	G	G	1501

G	G	A	C	A	T	A	T	A	G	G	G	A	T	A	T	G	G	G	G	1501
G	G	G	T	G	T	A	T	G	G	G	G	A	T	A	A	G	C	G	G	1501
C	G	G	T	A	T	G	T	G	G	T	G	A	T	A	A	G	C	G	G	1502
C	G	G	T	A	T	A	T	G	G	T	G	A	T	A	A	G	C	G	A	1502
G	G	A	C	A	T	A	T	G	G	T	G	A	T	A	A	G	C	G	G	1502
C	G	G	T	A	T	G	T	G	G	T	G	A	T	A	A	G	C	G	A	1502
C	A	G	T	A	G	G	T	G	A	T	G	G	T	A	T	G	G	A	G	1503
C	A	G	T	A	G	G	C	G	A	T	G	G	T	A	T	G	G	G	G	1503
C	G	A	T	G	T	A	C	G	A	T	A	G	C	T	T	A	G	G	G	1503
G	G	A	C	A	T	A	T	A	G	G	G	A	T	A	A	G	C	G	A	1508
C	G	A	T	G	T	A	C	G	A	G	G	A	T	A	A	G	C	G	G	1511
C	G	G	T	A	G	G	C	G	A	G	G	A	T	A	A	G	C	G	G	1511
C	G	G	T	A	T	A	T	G	G	T	G	A	T	A	A	G	C	G	G	1518
C	G	G	T	A	G	G	T	G	G	T	G	A	T	A	A	G	C	G	G	1518
C	G	G	T	A	T	G	T	G	G	T	G	A	T	A	A	G	C	G	G	1525
C	G	A	T	A	T	G	T	G	G	T	G	A	T	A	A	G	C	G	G	1525
C	A	G	T	A	G	G	T	G	G	T	G	A	T	A	A	G	C	G	G	1525
C	G	G	T	G	T	A	C	G	A	G	G	A	T	A	A	G	C	G	G	1527
C	G	G	T	G	T	A	C	G	A	T	G	A	T	A	A	G	C	G	G	1527
C	G	A	T	A	G	G	T	G	G	G	G	A	T	A	A	G	C	G	G	1532
G	G	G	T	G	T	A	T	G	G	T	A	G	C	T	T	G	C	G	A	2704
G	G	G	T	G	T	A	T	G	G	T	A	G	C	T	T	G	C	G	G	2704
G	G	G	T	G	T	A	T	G	G	T	G	G	C	T	T	G	C	G	G	2704

C	G	A	T	A	T	A	T	G	G	T	A	G	T	T	T	G	C	G	G	2704
C	G	A	T	A	T	A	T	A	G	T	A	G	T	A	T	A	G	G	G	2704
C	G	G	T	A	T	G	C	A	G	T	A	G	C	T	T	A	G	G	A	2705
C	G	A	T	G	T	A	C	G	A	T	A	G	C	T	T	G	G	G	G	2706
C	G	G	T	G	T	A	C	G	A	T	A	G	C	T	T	A	G	G	G	3501
C	G	A	T	G	T	A	C	G	A	T	A	G	C	T	T	A	G	G	G	3501
C	G	G	T	A	T	G	T	G	G	T	A	G	C	T	T	A	G	G	A	3501
C	G	G	T	G	T	A	C	G	A	T	G	G	C	T	T	A	G	G	A	3501
C	A	G	T	G	T	A	C	G	A	T	A	G	C	T	T	A	G	G	G	3501
G	G	A	C	A	G	G	C	G	G	T	A	G	C	T	T	A	G	G	A	3501
C	G	A	T	G	T	A	C	G	A	T	G	A	T	A	T	A	G	G	G	3501
C	G	A	T	A	G	G	T	G	G	T	G	G	T	T	T	A	G	G	A	3502
C	G	A	T	G	T	A	T	G	A	T	A	G	C	T	T	A	G	G	G	3505
G	G	A	C	A	G	G	C	G	G	T	A	G	C	T	T	G	G	G	G	3701
G	G	A	C	A	G	G	C	G	G	T	A	G	C	T	T	G	G	A	G	3701
C	A	G	T	A	G	G	T	G	G	T	A	G	C	T	T	A	G	A	G	3802
C	A	G	T	A	G	G	T	G	G	T	A	G	C	T	T	A	G	G	G	3802
C	A	G	T	A	G	G	C	G	G	T	A	G	C	T	T	A	G	A	G	3802
C	A	G	T	A	G	G	T	G	G	T	G	G	C	T	T	A	G	A	G	3802
C	G	A	T	A	G	G	C	G	G	T	A	G	C	T	T	A	G	G	G	3802
C	G	A	T	A	T	A	T	G	G	T	A	G	T	T	T	G	C	G	G	3802
C	G	A	T	A	T	A	T	A	G	T	A	G	T	A	T	A	G	G	G	3802
C	A	G	T	A	G	G	T	G	G	T	G	A	T	A	A	G	G	G	G	3901

C	A	G	T	A	G	G	C	G	G	T	G	A	T	A	A	G	G	G	G	3901
C	A	G	T	A	G	G	T	G	G	T	A	G	C	T	T	A	G	G	G	3901
C	A	G	T	A	G	G	C	G	G	T	G	G	C	T	T	A	G	G	G	3901
C	G	G	T	A	G	G	T	G	G	T	A	G	C	T	T	A	C	G	G	3901
C	A	G	T	A	G	G	T	G	G	T	A	G	C	T	T	A	G	G	A	3901
C	A	G	T	A	G	G	T	G	A	T	G	G	T	A	T	G	G	G	G	4001
C	G	A	T	G	T	A	C	G	A	T	G	G	T	A	T	G	G	G	G	4001
C	A	G	T	A	G	G	C	G	A	T	G	G	T	A	T	G	G	G	G	4001
C	G	A	T	A	T	G	T	G	G	T	G	G	T	A	T	G	G	G	G	4001
C	G	G	T	A	T	A	T	G	G	T	G	G	T	A	T	G	G	G	G	4001
C	G	G	T	G	T	A	C	G	A	T	G	G	T	A	T	G	G	G	G	4001
C	G	G	T	A	G	G	C	G	A	T	G	G	T	A	T	G	G	G	G	4001
C	G	G	T	A	T	G	T	G	G	T	G	A	T	A	A	G	C	G	G	4001
G	A	G	T	A	G	G	T	G	A	T	G	G	T	A	T	G	G	G	G	4001
C	G	A	T	G	T	A	T	G	A	T	G	G	T	A	T	G	G	G	G	4001
C	G	A	T	G	T	A	C	G	A	T	G	G	T	A	T	G	G	G	A	4001
C	G	G	T	G	T	A	C	G	G	T	G	G	T	T	T	A	G	G	G	4001
C	G	G	T	G	T	A	C	G	A	T	G	G	T	T	T	A	G	G	G	4001
C	G	A	T	A	T	A	T	G	G	T	A	G	T	T	T	G	C	G	G	4002
C	G	A	T	A	T	A	T	G	G	T	A	G	C	T	T	G	C	G	A	4002
G	G	G	T	G	T	A	T	G	G	T	A	G	T	T	T	G	C	G	G	4002
C	G	A	T	G	T	A	C	G	A	T	A	G	T	T	T	A	G	A	G	4002
C	A	G	T	A	G	G	T	G	G	T	A	G	T	T	T	G	C	G	A	4002

C	A	G	T	A	G	G	T	G	G	T	G	G	T	T	T	G	C	G	G	4002
C	A	G	T	A	G	G	T	G	G	T	G	G	T	T	T	G	C	G	A	4002
C	G	A	T	A	T	A	T	G	G	T	G	G	T	T	T	G	C	G	G	4002
C	G	A	T	A	T	A	T	G	G	T	G	G	T	T	T	G	C	G	A	4002
C	G	A	T	A	T	A	T	G	G	T	A	G	T	T	T	G	C	G	G	4003
C	G	G	T	A	T	G	T	G	G	T	A	G	C	T	T	G	C	G	G	4006
C	G	G	T	A	T	G	T	G	G	T	A	G	T	T	T	G	C	G	G	4006
C	A	G	T	A	T	G	T	G	G	T	A	G	T	T	T	G	C	G	A	4006
C	G	G	T	A	T	A	C	G	A	T	G	G	C	A	T	G	G	G	G	4403
C	G	G	T	A	T	A	C	G	A	T	G	G	C	A	T	G	G	G	A	4403
C	G	A	T	G	T	A	C	G	A	T	G	G	C	A	T	G	G	G	A	4403
G	G	A	C	A	T	A	T	A	G	G	G	A	T	A	A	G	C	G	G	4601
G	G	A	T	A	T	A	T	A	G	G	G	A	T	A	A	G	C	G	G	4601
G	G	A	C	A	T	A	T	A	G	T	G	A	T	A	A	G	C	G	G	4601
C	G	G	T	A	T	A	T	G	G	T	G	A	T	A	A	G	C	G	G	4601
C	G	G	T	A	T	A	T	A	G	T	G	A	T	A	A	G	C	G	G	4601
C	G	G	T	A	G	G	C	G	A	G	G	A	T	A	A	G	C	G	G	4601
C	G	G	T	G	T	A	C	G	G	T	G	G	T	T	T	A	G	G	G	4601
C	G	G	T	G	T	A	C	G	A	T	G	G	T	T	T	A	G	G	G	4601
C	G	G	T	A	T	G	T	G	G	T	G	G	C	T	A	G	G	G	G	4801
C	G	G	T	A	T	G	T	G	G	T	G	G	C	T	A	G	C	G	G	4801
G	G	A	C	A	T	A	T	G	G	T	G	G	C	T	A	G	G	G	G	4801
G	G	G	T	G	T	A	T	G	G	T	G	A	T	A	T	G	G	G	G	5101

C	G	G	T	A	T	A	T	G	G	T	A	G	T	A	T	A	G	G	A	5101
G	G	G	T	G	T	A	T	G	G	T	A	G	C	T	T	A	G	G	G	5101
C	G	G	T	A	G	G	T	G	G	T	G	A	T	A	T	G	G	G	G	5101
G	G	A	C	A	T	A	T	A	G	T	G	A	T	A	T	G	G	G	G	5101
G	G	G	T	G	T	A	T	G	G	T	G	A	T	A	T	G	G	A	G	5101
C	G	A	T	A	G	G	T	G	G	T	A	G	T	A	T	A	G	G	A	5101
C	G	G	T	A	T	A	T	G	G	T	G	A	T	A	T	G	G	G	G	5102
C	G	G	T	A	T	A	T	G	G	T	G	A	T	A	T	G	G	G	A	5102
C	G	G	T	A	G	G	T	G	G	T	G	A	T	A	T	G	G	G	G	5102
C	G	A	T	G	T	A	C	G	G	T	G	A	T	A	A	G	G	G	G	5201
C	G	A	T	G	T	A	C	G	G	T	G	A	T	A	T	G	G	G	G	5201
C	G	A	T	A	T	A	T	A	G	T	A	G	T	A	T	A	G	G	A	5401
C	A	G	T	A	G	G	T	G	A	T	G	G	T	A	T	A	G	G	A	5401
C	G	A	T	A	T	A	T	A	G	T	A	G	T	A	T	G	G	G	A	5401
G	G	A	C	A	T	A	T	A	G	T	A	G	T	A	T	A	G	G	A	5502
C	G	G	T	A	T	A	T	G	G	T	A	G	T	A	T	A	G	G	A	5502
C	G	G	T	A	T	A	T	G	G	T	G	A	T	A	T	G	G	G	A	5502
G	G	A	C	A	T	A	T	A	G	T	G	G	T	A	T	A	G	G	A	5502
C	G	A	T	A	T	A	T	A	G	T	G	G	T	A	T	A	G	G	A	5502
C	G	A	T	A	T	G	T	G	G	T	A	G	T	A	T	A	G	G	G	5502
C	G	A	T	G	T	A	C	G	A	T	A	G	T	A	T	A	G	G	A	5502
C	G	G	T	A	T	A	T	A	G	T	A	G	T	A	T	A	G	G	G	5502
G	G	A	C	A	G	G	C	G	G	T	A	G	T	A	T	A	G	G	A	5502

C	G	G	T	A	T	A	T	G	G	T	G	G	T	A	T	A	G	G	A	5502
C	G	A	T	A	T	A	T	A	G	T	G	A	T	A	A	G	C	G	G	5504
C	A	G	T	A	T	A	T	A	G	T	A	G	T	A	T	A	G	G	G	5601
G	G	A	C	A	T	A	T	A	G	T	A	G	T	A	T	A	C	G	G	5601
C	G	G	T	A	T	G	T	G	G	T	A	G	C	T	A	G	G	A	G	5601
C	A	G	T	G	T	A	C	G	A	T	A	G	T	A	T	A	G	G	G	5601
G	G	A	C	A	T	A	T	A	G	T	G	G	T	A	T	A	G	G	A	5603
C	A	G	T	A	T	A	T	A	G	T	A	G	T	A	T	A	G	G	G	5604
G	G	A	C	A	T	A	T	A	G	T	A	G	T	A	T	A	G	G	G	5604
G	G	A	C	A	G	G	C	G	G	T	A	G	C	T	T	A	G	G	A	5701
C	G	A	T	G	T	A	C	G	A	T	G	A	T	A	T	A	G	G	G	5701
C	G	G	T	G	T	A	C	G	G	T	A	G	C	T	T	A	G	G	G	5801
C	G	G	T	G	T	A	C	G	G	T	G	G	C	T	T	A	G	G	G	5801
C	G	G	T	G	T	A	T	G	G	T	A	G	C	T	T	A	G	G	G	5801
C	G	G	T	G	T	A	C	G	G	T	A	G	T	T	T	A	G	G	G	5801
C	G	G	T	G	T	A	T	G	G	T	A	G	C	T	T	A	G	G	A	5801
G	G	A	C	A	T	A	T	A	G	T	A	G	T	A	T	A	G	G	A	5901
C	A	G	T	A	G	G	T	G	G	T	A	G	C	T	T	A	G	G	G	6701
C	A	G	T	A	G	G	C	G	G	T	A	G	C	T	T	A	G	G	G	6701
C	A	G	T	A	G	G	C	G	G	T	G	G	T	A	T	G	G	G	G	6701

Without impute, *HLA-B*, Affy 6.0

C	G	A	G	A	G	G	C	G	G	A	A	A	G	T	C	G	A	G	T	C	T	A	T	702
C	G	A	G	A	G	G	A	A	G	A	A	G	G	C	C	G	G	A	C	C	T	A	T	705
T	G	G	G	A	A	G	A	A	A	G	G	A	G	T	C	G	G	A	T	T	T	A	G	705
C	G	A	G	A	G	G	C	G	G	G	G	A	G	T	C	G	G	A	C	T	T	A	G	801
C	G	A	G	A	G	G	C	G	G	A	A	G	G	C	C	A	G	A	C	C	C	C	G	801
T	G	G	G	A	G	G	C	G	G	A	A	G	G	C	C	G	G	A	T	T	T	A	T	1301
C	G	A	G	A	G	A	A	A	G	A	A	G	G	C	C	G	G	A	T	T	T	A	T	1301
C	G	G	T	A	A	A	C	G	G	A	A	G	G	C	C	G	G	A	T	T	T	A	T	1301
C	G	G	G	A	G	G	C	G	G	A	A	A	G	T	C	G	A	G	T	C	C	A	T	1302
C	G	G	G	A	G	A	C	G	G	A	A	A	G	T	C	G	A	G	T	C	C	A	T	1302
C	G	A	G	A	A	G	A	A	G	A	G	A	A	C	C	G	A	A	T	C	C	A	T	1501
C	G	G	G	A	A	A	C	G	G	A	G	A	A	C	C	G	A	A	T	C	C	A	T	1501
C	G	A	G	A	G	G	C	G	G	A	G	A	A	C	C	G	A	A	T	C	C	A	T	1501
C	G	G	G	A	A	A	C	G	G	A	G	A	A	C	C	G	G	A	T	C	T	A	T	1501
C	G	G	G	A	G	A	C	G	G	G	G	A	G	C	C	A	G	A	T	T	C	A	T	1501
C	C	G	G	G	G	G	A	G	G	A	G	A	A	C	C	G	A	A	T	C	C	A	T	1502
C	G	G	G	A	A	A	A	G	G	A	G	A	A	C	C	G	A	A	T	C	C	A	T	1502
C	G	G	G	A	A	G	A	G	G	A	G	A	A	C	C	G	A	A	T	C	C	A	T	1502
C	C	G	G	G	G	A	A	G	G	A	G	A	A	C	C	G	A	A	T	C	C	A	T	1503
C	G	A	G	A	G	G	A	A	G	A	A	G	G	C	C	G	G	A	C	C	T	A	T	1503
T	G	G	G	A	A	G	A	A	A	G	G	A	G	T	C	G	G	A	T	T	T	A	G	1503
C	G	G	G	A	A	A	C	G	G	A	G	A	A	C	C	G	A	A	T	C	C	A	T	1508
T	G	G	G	A	A	G	A	A	G	A	G	A	A	C	C	G	A	A	T	C	C	A	T	1511

C	G	G	G	A	A	A	A	G	G	A	G	A	A	C	C	G	A	A	T	C	C	A	T	1518
C	C	G	G	G	G	A	A	G	G	A	G	A	A	C	C	G	A	A	T	C	C	A	T	1518
C	G	A	G	A	G	G	A	G	G	A	G	A	A	C	C	G	A	A	T	C	C	A	T	1525
C	G	A	G	A	G	A	C	G	G	A	G	A	A	C	C	G	A	A	T	C	C	A	T	1525
C	G	A	G	A	A	G	A	A	G	A	G	A	A	C	C	G	A	A	T	C	C	A	T	1527
C	G	G	G	A	G	G	C	G	G	A	G	A	A	C	C	G	A	A	T	C	C	A	T	1532
C	G	G	G	A	A	A	C	G	A	G	G	A	G	T	T	G	G	A	T	C	C	A	T	2704
T	G	G	G	A	A	G	C	G	A	G	G	A	G	T	T	G	G	A	T	C	C	A	T	2704
C	G	A	G	A	G	G	C	G	A	G	G	A	G	T	T	G	G	A	T	T	T	C	G	2705
T	G	G	G	A	A	G	A	A	A	G	G	A	G	T	T	G	G	A	T	C	T	A	T	2706
C	G	A	G	A	A	G	A	A	A	G	G	A	G	T	C	G	G	A	T	T	T	A	G	3501
C	G	G	G	A	A	G	A	A	A	G	G	A	G	T	C	G	G	A	T	T	T	A	G	3501
T	G	G	G	A	A	G	A	A	A	G	G	A	G	T	C	G	G	A	T	T	T	A	G	3501
C	G	G	G	A	G	A	C	G	A	G	G	A	G	T	C	G	G	A	T	T	T	A	G	3502
T	G	G	G	A	A	G	C	G	A	G	G	A	G	T	C	G	G	A	T	T	T	A	G	3505
C	G	G	G	A	G	G	C	G	A	G	G	A	G	T	T	G	G	A	C	C	C	C	G	3701
C	G	A	G	A	G	G	C	G	G	G	G	A	G	T	C	G	G	A	C	T	T	A	G	3802
C	G	A	G	A	G	A	C	G	G	G	G	A	G	T	C	G	G	A	C	T	T	A	G	3802
T	G	G	G	A	A	A	C	G	G	G	G	A	G	C	C	A	G	A	T	T	C	A	T	3802
C	G	A	G	A	G	A	C	G	G	G	G	A	G	T	C	G	G	A	C	C	T	A	T	3802
C	G	A	G	A	G	G	C	G	G	A	G	A	A	C	C	G	A	A	T	C	T	A	T	3901
C	G	A	G	A	G	G	C	G	G	G	G	A	G	T	C	G	G	A	C	T	T	A	G	3901
C	G	A	G	A	G	G	C	G	G	G	G	A	G	T	C	G	G	A	C	C	C	A	T	3901

C	G	A	G	A	G	A	C	G	G	A	G	A	A	C	C	G	A	A	T	C	T	A	T	3901
C	G	A	G	A	G	G	A	A	G	A	A	G	G	C	C	G	G	A	C	C	T	A	T	4001
C	G	A	G	A	G	A	A	A	G	A	A	G	G	C	C	G	G	A	C	C	T	A	T	4001
T	G	G	G	A	A	G	A	A	G	A	A	G	G	C	C	G	G	A	C	C	T	A	T	4001
C	G	A	G	A	G	G	A	G	G	A	A	G	G	C	C	G	G	A	C	C	C	A	T	4001
C	G	G	G	A	A	G	C	G	G	A	A	G	G	C	C	G	G	A	C	C	T	A	T	4001
C	G	A	G	A	A	G	A	A	G	A	A	G	G	C	C	G	G	A	C	C	T	A	T	4001
C	G	G	G	A	A	G	A	A	A	G	G	A	G	T	C	G	G	A	T	T	T	A	G	4001
C	G	A	G	A	G	G	A	A	G	A	A	G	G	C	C	G	G	A	C	C	C	A	T	4001
C	C	G	G	G	G	A	A	G	G	A	A	G	G	C	C	G	G	A	C	C	T	A	T	4001
T	G	G	G	A	A	G	C	G	A	G	G	A	G	T	T	G	G	A	T	C	C	A	T	4002
C	G	A	G	A	G	G	C	G	A	G	G	A	G	T	T	G	G	A	T	C	C	A	T	4002
C	G	G	G	A	A	A	C	G	A	G	G	A	G	T	T	G	G	A	T	C	C	A	T	4002
T	G	G	G	A	A	G	A	A	A	G	G	A	G	T	T	A	G	A	T	T	C	A	T	4002
T	G	G	G	A	A	G	C	G	A	G	G	A	G	T	T	G	G	A	T	C	C	A	T	4003
C	C	G	G	G	G	A	A	G	A	G	G	A	G	T	T	G	G	A	T	C	C	A	T	4006
C	G	G	G	A	A	G	A	A	A	A	A	G	G	C	C	G	A	A	T	C	C	A	T	4403
C	G	A	G	A	A	G	A	A	A	A	A	G	G	C	C	G	A	A	T	C	C	A	T	4403
C	G	G	T	A	A	A	C	G	G	A	G	A	A	C	C	G	A	A	T	C	C	A	T	4601
C	G	G	G	A	A	A	C	G	G	A	G	A	A	C	C	G	A	A	T	C	C	A	T	4601
C	G	A	G	A	A	G	A	A	G	A	G	A	A	C	C	G	A	A	T	C	C	A	T	4601
C	G	G	G	A	A	G	C	G	A	G	G	A	G	T	C	G	G	A	T	T	T	A	G	4601
C	C	G	G	G	G	G	A	G	G	A	G	A	A	C	C	G	A	A	T	C	C	A	T	4601

T	G	G	G	A	A	A	C	G	G	A	G	A	A	C	C	G	A	A	T	C	C	A	T	4601
C	C	G	G	G	G	A	A	G	G	A	G	A	G	C	C	G	G	A	C	C	C	A	T	4801
C	C	G	G	G	G	A	A	G	G	A	G	A	G	C	C	G	G	A	C	C	T	A	T	4801
C	G	G	G	A	A	G	C	G	G	A	G	A	G	C	C	G	A	A	T	T	C	A	T	4801
C	G	G	G	A	A	A	A	G	A	A	G	A	A	C	C	G	G	A	T	C	T	A	T	5101
C	G	G	G	A	A	G	C	G	A	G	G	A	G	C	C	A	G	A	T	C	C	A	T	5101
C	G	G	G	A	A	A	A	G	A	A	G	A	A	C	C	G	G	A	T	C	C	A	T	5101
C	G	G	G	A	A	A	A	G	A	G	G	A	G	T	C	G	G	A	C	T	T	A	G	5101
C	G	G	G	A	A	A	C	G	A	A	G	A	A	C	C	G	G	A	T	C	T	A	T	5101
C	G	A	G	A	G	A	C	G	A	A	G	A	A	C	C	G	G	A	T	C	T	A	T	5101
C	G	G	G	A	A	G	C	G	A	A	G	A	A	C	C	G	G	A	T	C	T	A	T	5102
C	G	A	G	A	G	A	C	G	A	A	G	A	A	C	C	G	G	A	T	C	T	A	G	5102
C	G	A	G	A	A	G	C	G	A	A	G	A	A	C	C	G	A	A	T	C	T	A	T	5201
C	G	A	G	A	A	G	C	G	A	A	G	A	A	C	C	G	G	A	T	C	T	C	G	5201
T	G	G	G	A	A	A	C	G	G	G	G	A	G	C	C	A	G	A	T	T	C	A	T	5401
T	G	G	G	A	A	A	C	G	G	G	G	A	G	C	C	A	G	A	T	C	T	A	T	5401
C	G	A	G	A	G	G	A	A	G	G	G	A	G	C	C	A	G	A	T	T	C	A	T	5401
C	G	G	G	A	A	A	C	G	G	G	G	A	G	C	C	A	G	A	T	T	C	A	T	5502
C	G	G	G	A	A	G	C	G	G	G	G	A	G	C	C	A	G	A	T	T	C	A	T	5502
C	G	G	G	A	G	G	C	G	G	A	G	A	A	C	C	G	G	A	T	T	T	A	T	5502
C	G	G	G	A	G	G	C	G	G	G	G	A	G	C	C	A	G	A	C	T	T	A	G	5502
T	G	G	G	A	A	G	A	A	G	G	G	A	G	C	C	A	G	A	T	T	C	A	T	5502
C	C	G	G	G	G	A	A	G	G	A	G	A	A	C	C	G	A	A	T	C	C	A	T	5502

T	G	G	G	A	A	A	C	G	G	G	G	A	G	C	C	A	G	A	T	T	C	A	T	5502
T	G	G	G	A	A	A	C	G	G	A	G	A	A	C	C	G	A	A	T	C	C	A	T	5504
C	G	G	G	A	A	A	C	G	G	G	G	A	G	C	C	A	G	A	T	C	C	A	T	5601
C	G	A	G	A	A	G	A	A	G	G	G	A	G	C	C	A	G	A	T	T	C	A	T	5601
C	G	G	G	A	A	A	C	G	G	A	A	A	G	T	C	G	A	G	T	C	T	A	T	5601
C	G	G	G	A	A	A	C	G	G	G	G	A	G	C	C	A	G	A	T	T	C	A	T	5603
C	G	G	G	A	A	A	C	G	G	G	G	A	G	C	C	A	G	A	T	T	C	A	T	5604
C	G	A	G	A	A	A	C	G	G	G	G	A	G	C	C	A	G	A	T	T	C	A	T	5604
C	G	G	G	A	G	G	C	G	G	A	G	A	A	C	C	G	G	A	T	T	T	A	T	5701
C	G	G	G	A	A	G	C	G	A	G	G	A	G	T	C	G	G	A	T	T	T	A	G	5801
C	G	A	G	A	A	G	A	A	A	G	G	A	G	T	C	G	G	A	T	T	T	A	G	5801
C	G	G	G	A	A	A	C	G	G	G	G	A	G	C	C	A	G	A	T	T	C	A	T	5901
C	G	A	G	A	G	A	C	G	G	G	G	A	G	T	C	G	G	A	C	T	T	A	G	6701
C	G	A	G	A	G	G	C	G	G	G	G	A	G	T	C	G	G	A	C	T	T	A	G	6701
C	G	A	G	A	G	G	C	G	G	A	A	G	G	C	C	A	G	A	C	C	C	C	G	6701

Without impute, *HLA-B*, Illumina 550K

A	T	G	T	T	C	T	G	C	A	C	C	T	T	T	A	T	C	702
A	T	G	C	T	C	T	T	C	C	C	T	T	C	T	A	C	C	705
A	T	G	C	T	C	T	T	T	A	C	C	C	T	T	A	T	C	705
A	T	G	C	T	T	T	G	C	C	C	T	T	C	C	G	T	C	801
A	C	G	T	C	C	T	G	T	C	C	T	T	T	T	A	C	C	1301
A	C	G	T	C	C	T	G	T	C	C	T	T	T	C	A	C	C	1301

A	T	A	T	T	C	T	G	T	C	C	T	T	T	T	A	C	C	1301
A	T	G	C	T	C	T	G	T	C	C	T	T	T	C	A	C	C	1301
A	C	A	T	C	C	T	G	T	C	C	T	T	T	T	A	C	C	1301
A	C	G	T	C	T	T	G	C	A	C	C	T	T	T	A	T	C	1302
A	C	G	T	C	T	T	G	C	A	C	T	T	T	T	A	T	C	1302
A	T	A	C	T	C	T	T	T	A	T	C	C	T	C	A	T	C	1501
A	T	A	T	T	C	T	T	T	A	T	C	C	T	C	A	T	C	1501
A	T	A	T	T	C	T	T	T	A	T	C	T	T	T	A	C	C	1501
A	C	G	C	T	T	T	T	T	A	T	C	C	T	C	A	T	C	1501
A	T	G	T	C	T	T	T	T	A	T	C	C	T	C	A	T	C	1501
G	C	G	C	T	C	C	G	T	A	T	C	C	T	C	A	T	C	1501
A	C	G	T	C	C	T	T	T	A	C	T	T	T	T	A	T	C	1501
G	C	A	C	T	T	T	T	T	A	T	C	C	T	C	A	T	C	1502
A	T	A	C	T	T	T	T	T	A	T	C	C	T	C	A	C	C	1502
G	C	G	C	T	T	T	T	T	A	T	C	C	T	C	A	T	C	1502
A	C	G	C	T	C	C	T	T	A	T	C	C	T	C	A	C	C	1502
A	T	A	C	T	T	T	T	T	A	T	C	C	T	C	A	T	C	1502
A	T	G	C	T	T	C	T	T	A	C	C	C	T	C	A	C	C	1503
A	T	G	C	T	C	T	T	C	C	C	T	T	C	T	A	C	C	1503
A	T	G	C	T	C	T	T	T	A	C	C	C	T	T	A	T	C	1503
A	T	A	T	T	C	T	T	T	A	T	C	C	T	C	A	T	C	1508
A	T	G	C	T	C	T	T	T	A	T	C	C	T	C	A	T	C	1511
A	T	A	C	T	T	C	T	T	A	T	C	C	T	C	A	C	T	1518

G	C	G	C	T	T	C	T	T	A	C	C	C	T	C	A	C	C	1518
G	T	A	C	T	T	C	T	T	A	T	C	C	T	C	A	C	C	1525
A	T	G	T	C	T	C	T	T	A	T	C	C	T	C	A	T	C	1525
A	T	A	C	T	C	T	T	T	A	T	C	C	T	C	A	T	C	1527
A	T	G	T	C	T	T	T	C	C	C	T	T	C	T	A	T	C	1532
A	C	G	C	T	T	C	G	T	A	C	C	T	T	C	A	T	C	2704
A	T	A	T	T	T	C	G	T	A	C	C	T	T	T	A	C	C	2704
A	C	G	C	T	C	C	G	T	A	C	C	T	T	C	A	T	C	2704
G	C	G	C	T	T	C	G	T	A	C	C	T	T	C	A	T	C	2704
A	C	A	T	T	T	T	G	T	A	C	C	T	T	T	G	C	C	2705
A	T	G	C	T	C	T	G	T	A	C	C	T	T	T	A	C	C	2706
A	T	G	C	T	C	T	T	T	A	C	C	C	T	T	A	T	C	3501
A	T	G	C	T	C	C	T	T	A	C	C	C	T	T	A	T	C	3501
G	C	A	C	T	C	C	T	T	A	C	C	C	T	T	A	T	C	3501
A	T	A	C	T	C	C	T	T	A	C	C	C	T	T	A	T	C	3501
A	C	G	T	C	T	C	T	T	A	C	C	C	T	T	A	C	C	3502
A	T	G	C	T	C	C	T	T	A	C	C	C	T	T	A	T	C	3505
A	C	G	T	C	T	C	G	T	A	C	C	C	C	T	G	C	T	3701
A	T	G	T	C	T	C	G	T	A	C	C	C	C	T	A	C	C	3802
A	T	A	T	T	C	C	G	T	A	C	T	T	T	T	A	C	C	3802
A	T	G	T	C	T	C	G	T	A	C	C	C	C	T	A	T	C	3802
A	T	G	T	C	T	C	G	T	A	T	C	C	T	C	A	C	C	3901
A	T	G	T	C	T	C	G	T	A	C	C	C	C	T	A	T	C	3901

A	T	G	T	C	T	C	G	T	A	C	C	C	C	T	A	C	C	3901
A	T	G	T	C	T	C	G	T	C	T	C	C	T	C	A	C	C	3901
A	T	G	C	T	C	T	T	C	C	C	T	T	C	T	A	C	C	4001
G	T	A	C	T	T	T	T	C	C	C	T	T	C	T	A	T	C	4001
A	T	A	T	T	T	T	T	C	C	C	T	T	C	T	A	C	C	4001
A	T	G	C	T	C	T	T	C	C	C	T	T	C	T	A	T	C	4001
A	T	A	C	T	C	T	T	C	C	C	T	T	C	T	A	C	C	4001
G	C	A	C	T	T	T	T	T	A	T	C	C	T	C	A	T	C	4001
G	C	G	C	T	T	T	T	C	C	C	T	T	C	T	A	C	C	4001
A	T	G	C	T	C	T	T	T	A	C	C	C	T	T	A	T	C	4001
A	T	A	T	T	T	C	G	T	A	C	C	C	T	T	A	C	C	4002
A	T	A	T	T	T	C	G	T	A	C	C	T	T	T	A	C	C	4002
A	T	G	C	T	C	C	G	T	A	C	C	C	T	T	A	C	C	4002
A	T	G	T	C	T	C	G	T	A	C	C	T	T	T	A	C	C	4002
G	C	G	C	T	T	C	G	T	A	C	C	T	T	T	A	C	C	4002
A	T	G	T	C	T	T	G	T	A	C	C	C	T	T	A	C	C	4002
A	T	A	T	T	T	C	G	T	A	C	C	C	T	T	A	C	C	4003
G	C	G	C	T	C	C	G	T	A	C	C	T	T	T	A	C	C	4006
G	C	G	C	T	C	C	G	T	A	C	C	C	T	T	A	C	C	4006
A	C	A	C	T	C	C	T	T	A	C	T	T	T	T	A	T	C	4403
A	T	A	C	T	C	C	T	T	A	C	T	T	T	T	A	C	C	4403
A	T	A	T	T	C	T	T	T	A	T	C	C	T	C	A	T	C	4601
A	T	A	C	T	C	T	T	T	A	T	C	C	T	C	A	T	C	4601

A	C	G	C	T	C	C	T	T	A	C	C	C	T	T	A	T	C	4601
A	T	A	T	T	C	C	T	T	A	C	C	C	T	T	A	T	C	4601
A	T	A	T	T	C	T	T	T	A	T	C	C	T	T	A	T	C	4601
A	T	A	T	T	C	T	T	T	A	C	C	C	T	C	A	T	C	4601
G	C	G	C	T	C	C	G	T	A	C	T	T	C	T	A	C	C	4801
G	C	G	C	T	C	C	G	T	A	C	T	T	C	T	A	T	C	4801
A	T	A	T	T	T	C	G	T	A	C	T	T	T	C	A	T	C	4801
G	C	G	C	T	C	C	G	T	A	C	T	T	C	C	A	T	C	4801
A	C	G	C	T	C	C	T	T	A	T	C	T	T	T	A	C	C	5101
G	C	G	C	T	C	C	T	T	A	T	C	T	T	T	A	T	C	5101
A	C	G	C	T	C	C	T	T	A	C	C	C	C	T	A	T	C	5101
A	T	A	T	T	T	C	T	T	A	C	T	T	T	T	A	C	C	5101
A	T	G	T	C	T	C	T	T	A	T	C	T	T	T	A	C	C	5101
A	T	A	T	T	C	C	T	T	A	T	C	T	T	T	A	C	C	5101
A	C	G	C	T	C	C	T	T	A	C	C	T	T	T	A	C	C	5101
A	C	G	T	C	C	C	T	T	A	C	T	T	T	T	A	C	C	5101
A	T	A	T	T	T	C	T	T	A	T	C	T	T	T	A	C	C	5102
A	T	G	T	C	T	C	T	T	A	T	C	T	T	T	A	C	C	5102
A	C	A	T	C	T	C	T	T	A	T	C	C	T	C	A	C	C	5201
A	C	A	T	C	T	C	T	T	A	T	C	T	T	T	G	C	C	5201
A	T	A	T	T	C	C	G	T	A	C	T	T	T	T	A	C	C	5401
A	T	A	T	T	C	C	G	T	A	C	C	T	T	T	A	C	C	5401
A	T	A	T	T	C	C	T	T	A	C	T	T	T	T	A	C	C	5401

A	T	G	C	T	C	C	G	T	A	C	T	T	T	T	A	C	C	5401
A	T	A	T	T	C	C	G	T	A	C	T	T	T	T	A	T	C	5401
A	T	A	T	T	C	C	G	T	A	C	T	T	T	T	A	C	C	5502
A	T	A	T	T	C	T	G	T	A	C	T	T	T	T	A	C	C	5502
A	T	A	T	T	T	C	G	T	A	C	T	T	T	T	A	C	C	5502
A	T	A	T	T	C	T	G	T	A	C	T	T	T	T	A	T	C	5502
A	T	A	T	T	T	C	T	T	A	T	C	T	T	T	A	C	C	5502
A	T	G	C	T	C	T	G	T	A	C	T	T	T	T	A	C	C	5502
A	C	G	T	C	C	C	G	T	A	C	T	T	C	T	A	C	C	5502
G	C	G	C	T	C	C	G	T	A	T	C	C	T	C	A	T	C	5502
A	C	G	T	C	C	T	T	T	A	C	T	T	T	T	A	T	C	5502
A	T	A	T	T	C	C	G	T	A	T	C	C	T	C	A	T	C	5504
A	T	A	T	T	C	C	G	T	A	C	T	T	T	T	A	T	C	5601
A	T	G	C	T	C	T	G	T	A	C	T	T	T	T	A	C	C	5601
G	C	G	C	T	T	C	G	C	A	C	C	T	T	C	A	C	C	5601
A	T	A	T	T	C	T	G	T	A	C	T	T	T	T	A	C	C	5603
A	T	A	T	T	C	C	G	T	A	C	T	T	T	T	A	C	C	5604
A	C	G	T	C	T	T	T	T	A	C	C	T	T	T	A	C	C	5701
A	C	G	C	T	C	C	T	T	A	C	C	C	T	T	A	T	C	5801
A	T	A	T	T	C	C	G	T	A	C	T	T	T	T	A	C	C	5901
A	T	G	T	C	T	C	G	T	A	C	C	C	C	T	A	T	C	6701

Without impute, *HLA-B*, Union

G	G	C	G	A	T	A	G	G	C	G	A	C	C	G	A	C	T	T	T	C	A	G	C	G	G	A	702
G	T	A	A	A	T	A	A	T	T	A	G	C	C	G	A	T	T	T	T	C	G	G	T	G	G	A	705
G	T	C	G	A	T	A	G	G	C	A	A	C	C	G	G	C	T	T	A	C	G	A	C	G	G	G	801
G	T	C	G	G	T	A	G	G	T	A	A	C	C	G	G	C	T	T	A	C	G	G	T	G	G	A	1301
A	T	C	G	A	T	A	G	G	T	A	A	C	C	G	G	C	T	T	A	C	G	G	T	G	G	A	1301
G	T	A	A	A	T	A	G	G	T	A	A	C	C	G	G	C	T	T	A	C	G	G	T	G	G	A	1301
A	T	C	G	G	T	A	G	G	T	A	A	C	C	G	G	C	T	T	A	C	G	G	T	G	G	A	1301
G	T	C	G	G	T	A	G	G	C	G	A	C	C	G	A	C	T	T	T	C	A	G	C	G	G	A	1302
A	T	A	A	A	T	A	G	T	T	A	A	T	T	A	G	T	C	T	A	C	A	A	C	G	G	A	1501
A	T	C	G	A	T	A	G	T	T	A	A	T	T	A	G	T	C	T	A	C	A	A	C	G	G	A	1501
G	T	C	G	G	T	A	G	T	T	A	A	T	T	A	G	T	C	T	A	C	A	A	C	G	G	A	1501
A	T	C	G	A	T	A	G	T	T	A	A	T	T	A	G	C	C	T	A	C	G	G	C	A	G	A	1501
G	T	A	G	A	T	A	G	T	T	A	A	T	T	A	G	T	C	T	A	C	A	A	C	G	A	A	1501
G	T	C	G	A	T	A	G	T	T	A	A	T	T	A	G	T	C	T	A	C	A	A	C	G	G	A	1501
A	T	A	G	A	T	A	G	T	T	A	A	T	T	A	G	T	C	T	A	C	A	A	C	G	G	A	1502
A	T	A	G	A	C	A	G	T	T	A	A	T	T	A	G	T	C	T	A	C	A	A	C	G	G	A	1502
G	T	A	G	A	T	A	G	T	T	A	A	T	T	A	G	T	C	T	A	C	A	A	C	G	G	A	1502
A	T	A	G	A	T	A	G	T	T	A	A	T	T	A	G	T	C	T	A	C	A	A	C	G	A	A	1502
G	T	A	A	A	T	A	G	T	C	A	A	T	C	G	G	C	T	T	A	C	G	G	C	G	G	A	1503
G	T	A	G	A	C	A	G	T	T	A	A	T	C	A	G	T	C	T	A	C	A	G	C	G	G	A	1503
A	T	C	G	A	T	A	G	T	T	A	A	T	T	A	G	T	C	T	A	C	A	A	C	G	G	A	1508
G	T	A	A	A	T	A	G	T	T	A	A	T	T	A	G	T	C	T	A	C	A	A	C	G	G	A	1511

A	T	A	G	A	C	A	G	T	T	A	A	T	T	A	G	T	C	T	A	C	A	G	C	G	G	A	1518
G	T	A	G	A	C	A	G	T	T	A	A	T	C	A	G	T	C	T	A	C	A	G	C	G	G	A	1518
A	T	A	G	A	C	A	G	T	T	A	A	T	T	A	G	T	C	T	A	C	A	A	C	G	G	A	1525
A	T	A	G	A	T	A	G	T	T	A	A	C	T	A	G	T	C	T	A	C	A	A	C	G	G	A	1525
G	T	C	G	G	C	C	G	T	T	A	A	T	T	A	G	T	C	T	A	C	A	A	C	G	G	A	1525
A	T	A	A	A	T	A	G	T	T	A	A	T	T	A	G	T	C	T	A	C	A	A	C	G	G	A	1527
G	T	C	G	G	T	A	G	T	T	A	A	T	T	A	G	T	C	T	A	C	A	G	C	G	G	A	1532
G	T	C	G	A	C	A	A	G	T	A	G	C	C	G	A	C	T	T	T	T	G	A	C	G	G	A	2704
A	G	C	G	A	C	A	A	G	T	A	G	C	C	G	G	C	T	T	T	T	G	G	C	G	G	A	2704
A	T	C	G	A	T	A	A	G	T	A	G	C	C	G	A	C	T	T	T	T	G	G	T	G	G	G	2705
G	T	A	A	A	T	A	A	G	T	A	G	C	C	G	A	C	T	T	T	T	G	G	C	G	G	A	2706
G	T	A	A	A	T	A	A	T	T	A	G	C	C	G	A	T	T	T	T	C	G	G	T	G	G	A	3501
G	T	A	A	A	C	C	A	T	T	A	G	C	C	G	A	T	T	T	T	C	G	G	T	G	G	A	3501
A	T	A	A	A	C	C	A	T	T	A	G	C	C	G	A	T	T	T	T	C	G	G	T	G	G	A	3501
A	T	A	G	A	C	C	A	T	T	A	G	C	C	G	A	T	T	T	T	C	G	G	T	G	G	A	3501
A	T	A	G	A	T	A	A	T	T	A	G	C	C	G	A	T	T	T	T	C	G	G	T	G	G	A	3501
G	T	A	G	A	C	C	A	T	T	A	G	C	C	G	A	T	T	T	T	C	G	G	T	G	G	A	3502
G	T	C	G	G	C	A	A	G	T	A	G	C	C	G	A	T	T	T	T	T	G	G	C	G	G	A	3502
G	T	A	A	A	C	C	A	T	T	A	G	C	C	G	A	T	T	T	T	C	G	G	T	G	G	A	3505
G	T	C	G	G	C	A	A	G	T	A	G	C	C	G	A	T	T	T	T	T	G	G	C	G	G	G	3701
G	T	C	G	G	C	A	G	G	T	A	G	C	C	G	A	T	T	A	T	C	G	G	T	G	G	A	3802
A	T	C	G	A	C	C	G	G	T	A	G	C	C	G	G	C	T	T	A	C	G	G	T	G	A	A	3802
G	T	C	G	G	C	A	G	G	T	A	G	C	C	G	A	T	T	A	T	C	G	G	C	G	G	A	3802

G	T	C	G	G	C	A	G	G	T	A	A	T	T	A	G	T	C	T	A	C	A	G	C	G	G	A	3901
G	T	C	G	G	C	A	G	G	T	A	G	C	C	G	A	T	T	A	T	C	G	G	T	G	G	A	3901
G	T	C	G	G	C	A	G	G	T	A	G	C	C	G	A	T	T	A	T	C	G	G	C	G	G	A	3901
G	T	A	A	A	T	A	G	T	C	A	A	T	C	G	G	C	T	T	A	C	G	G	C	G	G	A	4001
A	T	A	G	A	T	A	G	T	C	A	A	T	C	G	G	C	T	T	A	C	G	G	C	G	G	A	4001
A	G	C	G	A	T	A	G	T	C	A	A	T	C	G	G	C	T	T	A	C	G	G	C	G	G	A	4001
A	T	A	A	A	T	A	G	T	C	A	A	T	C	G	G	C	T	T	A	C	G	G	C	G	G	A	4001
G	T	A	A	A	T	A	A	T	T	A	G	C	C	G	A	T	T	T	T	C	G	G	T	G	G	A	4001
A	G	C	G	A	C	A	A	G	T	A	G	C	C	G	G	T	T	T	T	T	G	G	C	G	G	A	4002
G	T	C	G	A	C	A	A	G	T	A	G	C	C	G	G	C	T	T	T	T	G	G	C	G	G	A	4002
A	G	C	G	A	C	A	A	G	T	A	G	C	C	G	A	T	T	T	T	T	G	G	C	G	G	A	4002
G	T	C	G	G	C	A	A	G	T	A	G	C	C	G	G	T	T	T	T	T	G	G	C	G	G	A	4002
G	T	A	A	A	C	A	A	G	T	A	G	C	C	G	G	T	T	T	T	T	G	G	T	G	A	A	4002
G	T	C	G	G	T	A	A	G	T	A	G	C	C	G	G	T	T	T	T	T	G	G	C	G	G	A	4002
A	G	C	G	A	C	A	A	G	T	A	G	C	C	G	G	T	T	T	T	T	G	G	C	G	G	A	4003
G	T	A	G	A	C	A	A	G	T	A	G	C	C	G	G	C	T	T	T	T	G	G	C	G	G	A	4006
G	T	A	G	A	C	A	A	G	T	A	G	C	C	G	G	T	T	T	T	T	G	G	C	G	G	A	4006
G	T	C	G	G	C	A	A	G	T	A	G	C	C	G	A	T	T	T	T	T	G	G	C	G	G	A	4006
G	T	A	G	A	C	C	A	T	T	A	G	C	C	G	A	T	T	T	T	C	G	G	T	G	G	A	4006
A	T	A	A	A	C	A	A	T	T	G	A	T	C	G	G	C	T	T	A	C	A	G	C	A	G	A	4403
A	T	C	G	A	T	A	G	T	T	A	A	T	T	A	G	T	C	T	A	C	A	A	C	G	G	A	4601
A	T	A	A	A	T	A	G	T	T	A	A	T	T	A	G	T	C	T	A	C	A	A	C	G	G	A	4601
G	T	C	G	A	C	A	A	T	T	A	G	C	C	G	A	T	T	T	T	C	G	G	T	G	G	A	4601

A	T	C	G	A	T	A	G	T	T	A	A	T	C	A	G	T	C	T	A	C	A	A	C	G	G	A	4601
G	T	A	G	A	C	A	G	G	T	A	A	C	C	G	G	C	C	T	A	C	A	G	C	G	G	A	4601
A	G	C	G	A	C	A	G	T	C	G	A	T	C	A	A	T	T	T	T	C	A	G	C	G	G	A	4601
G	T	A	G	A	C	A	G	G	T	A	A	C	C	G	G	C	T	T	T	C	G	G	C	G	G	A	4801
G	T	A	G	A	C	A	G	G	T	A	A	C	C	G	G	C	T	T	T	C	G	A	C	G	G	A	4801
A	G	C	G	A	C	C	A	T	T	A	A	T	T	A	G	C	C	T	A	C	G	G	T	G	G	A	4801
G	T	A	G	A	C	A	G	G	T	A	A	C	C	G	G	C	T	T	T	C	A	A	C	G	G	A	4801
G	T	A	G	A	C	C	A	T	T	A	A	T	T	A	G	C	C	T	A	C	G	G	C	A	G	A	5101
A	G	C	G	A	C	C	A	T	T	A	G	C	C	G	G	C	T	T	A	C	G	G	C	G	A	A	5101
G	T	A	G	A	C	C	A	T	T	A	G	C	C	G	A	T	T	A	T	C	G	G	T	G	G	A	5101
G	T	C	G	G	C	C	G	T	T	A	G	C	C	G	G	C	T	T	A	C	G	G	T	G	A	A	5101
A	T	C	G	A	C	C	A	T	T	A	A	T	T	A	G	C	C	T	A	C	G	G	C	A	G	A	5101
G	T	A	G	A	C	C	A	T	T	A	A	T	C	A	G	C	C	T	A	C	G	G	C	A	G	A	5101
G	T	C	G	G	C	C	A	T	T	A	A	T	T	A	G	C	C	T	A	C	G	G	C	A	G	A	5101
A	G	C	G	A	C	C	A	T	T	A	A	T	T	A	G	C	C	T	A	C	G	G	T	G	G	A	5101
G	T	A	G	A	C	A	G	G	T	A	A	C	C	G	G	C	T	T	T	C	A	A	C	G	G	A	5101
A	G	C	G	A	C	C	A	T	T	A	A	T	T	A	G	C	C	T	A	C	G	G	C	A	G	A	5102
G	T	C	G	G	C	C	A	T	T	A	A	T	T	A	G	C	C	T	A	C	G	G	C	G	G	A	5102
A	T	C	G	G	C	C	A	T	T	A	A	T	T	A	G	T	C	T	A	C	A	G	C	A	G	A	5201
A	T	C	G	G	C	C	A	T	T	A	A	T	T	A	G	C	C	T	A	C	G	G	C	A	G	G	5201
A	T	C	G	A	C	C	G	G	T	A	G	C	C	G	G	C	T	T	A	C	G	G	T	G	A	A	5401
G	T	A	A	A	C	C	G	G	T	A	G	C	C	G	G	C	T	T	A	C	G	G	T	G	A	A	5401
A	T	C	G	A	C	C	G	T	T	A	G	C	C	G	G	C	T	T	A	C	G	G	C	A	G	A	5401

A	T	C	G	A	C	C	G	G	T	A	G	C	C	G	G	C	T	T	A	C	G	G	T	G	A	A	5502
A	T	C	G	A	T	C	G	G	T	A	G	C	C	G	G	C	T	T	A	C	G	G	T	G	A	A	5502
A	G	C	G	A	C	C	G	G	T	A	G	C	C	G	G	C	T	T	A	C	G	G	T	G	A	A	5502
G	T	C	G	G	C	C	G	G	T	A	G	C	C	G	G	C	T	T	A	C	G	G	T	G	G	A	5502
A	G	C	G	A	C	C	A	T	T	A	A	T	T	A	G	C	C	T	A	C	G	G	C	A	G	A	5502
G	T	A	A	A	T	A	G	G	T	A	G	C	C	G	G	C	T	T	A	C	G	G	T	G	A	A	5502
A	T	C	G	A	C	C	G	G	T	A	A	T	T	A	G	T	C	T	A	C	A	A	C	G	G	A	5504
A	T	C	G	A	C	C	G	G	T	A	G	C	C	G	G	C	T	T	A	C	G	G	C	G	G	A	5601
G	T	A	A	A	T	C	G	G	T	A	G	C	C	G	G	C	T	T	A	C	G	G	T	G	A	A	5601
G	T	A	G	A	C	A	G	G	T	A	A	C	C	G	G	C	C	T	A	C	A	G	C	G	G	A	5601
A	G	C	G	A	C	A	G	T	C	G	A	T	C	A	A	T	T	T	T	C	A	G	C	G	G	A	5601
A	T	C	G	A	T	A	G	G	T	A	G	C	C	G	G	C	T	T	A	C	G	G	T	G	A	A	5603
A	T	C	G	A	C	C	G	G	T	A	G	C	C	G	G	C	T	T	A	C	G	G	T	G	A	A	5604
A	T	C	G	A	C	A	G	G	T	A	G	C	C	G	G	C	T	T	A	C	G	G	T	G	A	A	5604
G	T	C	G	G	T	A	G	T	T	A	A	T	C	A	G	C	C	T	A	C	G	G	T	G	G	A	5701
G	T	C	G	A	C	A	A	T	T	A	G	C	C	G	A	T	T	T	T	C	G	G	T	G	G	A	5801
G	T	C	G	A	C	A	A	T	T	A	G	C	C	G	G	T	T	T	T	C	G	G	T	G	G	A	5801
A	T	C	G	A	C	C	G	G	T	A	G	C	C	G	G	C	T	T	A	C	G	G	T	G	A	A	5901
G	T	C	G	G	C	A	G	G	T	A	G	C	C	G	A	T	T	A	T	C	G	G	T	G	G	A	6701

Without impute, *HLA-C*, Affy 5.0

C	T	G	T	G	A	C	G	A	G	C	C	T	A	A	G	102
C	G	G	T	G	A	C	G	A	G	C	C	T	A	A	A	102

C	T	G	T	G	A	C	G	A	G	C	C	T	A	A	A	102
C	G	A	T	G	A	C	G	A	G	C	C	T	A	A	A	102
C	G	G	C	G	G	C	G	A	G	C	C	C	A	A	G	102
C	G	G	C	G	G	T	G	A	G	C	C	T	G	A	A	102
C	G	G	C	G	G	C	G	A	G	T	C	T	A	A	G	102
C	T	G	T	G	A	C	G	A	G	C	C	T	A	A	G	103
C	G	G	C	G	G	C	A	A	G	C	C	T	G	A	A	202
C	G	A	C	G	G	C	G	A	G	T	C	T	A	A	A	302
C	G	A	C	G	G	C	G	A	G	T	C	T	A	A	G	302
C	G	A	C	G	G	C	A	A	T	C	A	T	G	A	A	302
C	G	A	C	G	G	C	G	A	G	C	C	T	A	A	G	302
C	G	G	T	G	A	C	G	A	G	T	C	T	A	A	G	303
C	G	G	C	G	G	C	G	A	G	T	C	T	A	A	A	303
C	G	G	T	G	A	C	G	A	G	T	C	T	A	A	G	304
C	G	G	T	G	A	C	G	A	G	C	A	T	G	T	G	304
C	G	G	T	G	A	C	G	A	G	C	C	C	A	A	A	304
C	G	G	T	G	A	C	G	A	G	C	C	C	A	A	G	304
T	G	G	C	G	G	C	A	A	G	T	C	T	A	A	G	401
T	G	G	C	G	G	C	A	A	G	T	C	T	A	A	A	401
C	T	G	T	G	G	C	A	A	G	T	C	T	A	A	G	401
C	G	G	T	G	A	C	A	A	G	T	C	T	A	A	G	401
C	G	A	T	G	A	C	A	A	G	C	C	T	G	A	G	403
T	G	A	T	G	A	C	A	A	G	C	C	T	G	A	G	403

C	G	A	T	G	G	C	A	A	G	C	C	T	G	A	G	403
C	G	A	T	G	A	C	G	A	G	T	C	T	A	A	A	403
C	T	G	T	G	A	C	G	A	G	C	A	T	G	A	A	602
C	T	G	T	G	A	C	G	A	G	C	A	T	G	A	G	602
C	G	G	C	G	G	C	G	G	G	C	C	T	A	A	G	701
C	G	A	C	A	G	C	A	A	T	C	A	T	G	A	G	702
C	G	A	C	A	G	C	A	A	T	C	A	T	G	A	A	702
C	G	A	C	G	G	C	A	A	T	C	A	T	G	A	G	702
C	G	A	C	G	G	C	G	A	G	T	C	T	A	A	G	702
C	G	G	T	G	A	C	G	A	G	C	C	T	A	A	A	702
C	G	G	C	G	G	C	G	G	G	C	C	T	A	A	G	702
C	G	A	C	A	G	C	A	A	T	C	C	T	A	A	A	702
C	G	A	C	A	G	C	G	A	G	C	C	T	A	A	A	702
C	G	A	C	A	G	C	A	A	T	T	C	T	A	A	A	702
C	G	A	C	G	G	C	A	A	T	C	A	T	G	A	A	702
C	G	G	C	G	G	C	G	A	G	C	C	C	A	A	G	704
C	G	G	C	G	G	T	G	A	G	C	C	T	G	A	G	801
C	G	G	C	G	G	T	G	A	G	C	C	T	G	A	A	801
C	G	G	T	G	A	C	G	A	G	T	C	T	A	A	G	801
C	G	A	C	A	G	T	G	A	G	C	C	T	G	A	A	801
C	T	G	T	G	G	C	G	G	G	C	C	T	A	A	A	1202
C	T	G	T	G	G	C	G	G	G	C	C	T	A	A	G	1202
C	G	G	T	G	A	C	A	A	G	T	C	T	A	A	G	1202

C	G	G	C	G	A	C	A	A	G	T	C	T	A	A	G	1202
C	G	G	T	G	A	C	G	A	G	C	C	C	A	A	A	1202
C	G	G	T	G	A	C	A	A	G	T	C	T	A	A	A	1202
C	G	A	T	G	A	C	G	A	G	C	A	T	G	A	A	1203
C	G	A	T	G	A	C	G	A	G	C	A	T	G	A	G	1203
C	T	G	T	G	A	C	G	A	G	C	A	T	G	A	A	1203
C	T	G	T	G	G	C	G	A	G	T	C	T	A	A	G	1402
C	T	G	T	G	G	C	G	A	G	T	C	T	A	A	A	1402
C	G	A	C	G	G	C	G	A	G	C	C	C	A	A	G	1502
C	G	A	C	G	G	C	G	A	G	C	C	C	A	A	A	1502
C	T	G	T	G	A	C	G	A	G	C	C	C	A	A	G	1502
C	G	A	C	G	G	C	G	A	G	C	C	T	A	A	A	1502
C	G	A	C	A	G	C	A	A	T	C	A	T	G	A	A	1505

Without impute, *HLA-C*, Affy 6.0

T	G	C	C	A	T	C	C	A	G	G	T	G	C	A	C	A	102
T	G	C	C	A	T	C	C	A	G	G	G	G	C	A	C	A	102
T	G	T	T	A	T	C	C	A	G	G	G	G	C	A	C	A	102
T	G	C	C	A	T	C	C	A	G	G	T	G	T	A	C	A	102
T	G	T	C	A	T	T	T	A	C	G	G	A	C	G	C	G	102
C	A	T	C	A	T	C	T	A	G	G	G	G	C	G	C	G	102
T	G	T	C	A	T	C	T	A	G	G	G	A	T	A	C	G	102
T	G	C	C	A	T	C	C	A	G	G	T	G	C	A	C	A	103

C	A	T	C	A	T	C	C	G	G	A	G	G	C	A	C	G	202
C	A	T	C	A	T	C	T	A	G	G	G	A	T	A	C	G	302
C	A	T	C	A	T	C	T	A	G	G	G	G	T	A	C	G	302
C	A	T	C	A	T	C	T	G	G	G	G	A	T	A	C	G	302
C	A	T	C	A	T	C	T	G	G	G	G	A	C	A	C	G	302
T	G	T	T	A	T	C	T	A	G	G	G	A	T	A	C	G	303
T	G	T	C	A	T	C	T	A	G	G	G	A	T	A	C	G	303
T	G	T	T	A	T	C	T	A	G	G	G	A	C	A	C	A	303
T	G	T	T	A	T	C	T	A	G	G	G	A	T	A	C	G	304
T	G	T	T	A	T	C	T	A	G	G	G	A	C	A	C	G	304
T	G	T	T	A	T	C	T	A	G	G	G	A	C	G	C	G	304
T	G	T	T	A	T	T	T	A	G	G	G	A	C	A	C	G	304
C	A	T	C	A	T	C	C	G	G	A	G	A	T	A	C	G	401
T	G	T	C	A	T	C	C	G	G	A	G	A	T	A	C	G	401
C	A	T	C	A	T	C	C	G	G	A	G	G	T	A	C	G	401
T	G	T	C	A	T	C	C	G	G	A	G	G	C	A	C	G	403
T	G	T	C	A	G	C	C	G	G	A	G	G	C	A	C	G	403
T	G	C	C	A	T	C	C	G	G	G	G	G	C	A	T	G	602
C	A	T	C	T	T	T	T	A	G	G	G	A	C	A	C	A	701
C	A	T	C	T	G	C	C	G	G	A	G	G	C	A	C	G	702
C	A	T	C	A	T	C	T	A	G	G	G	A	T	A	C	G	702
T	G	T	T	A	T	C	T	A	G	G	G	A	C	G	C	G	702
C	A	T	C	T	T	T	T	A	G	G	G	A	C	A	C	A	702

C	A	T	C	T	T	C	C	A	G	G	G	G	C	A	C	A	702
C	A	T	C	A	T	C	T	A	G	G	G	A	C	G	C	G	704
C	A	T	C	T	T	C	T	A	G	G	G	A	C	G	C	G	704
T	G	T	C	A	T	T	T	A	C	G	G	A	C	G	C	G	801
C	A	T	C	T	G	C	C	G	G	A	G	G	C	A	C	G	801
T	G	T	C	A	T	C	C	G	G	G	G	A	C	A	C	A	1202
C	G	T	C	A	T	C	C	G	G	A	G	A	T	A	C	G	1202
T	G	T	T	A	T	C	T	A	G	G	G	A	C	A	C	A	1202
T	G	T	C	A	T	C	C	G	G	G	G	G	C	A	T	G	1203
T	G	C	C	A	T	C	C	G	G	G	G	G	C	A	T	G	1203
T	G	T	C	A	T	C	C	A	G	G	G	G	T	A	C	G	1402
C	A	T	C	A	T	C	C	A	G	G	G	G	T	A	C	G	1402
T	G	T	C	A	T	C	C	A	G	G	G	G	T	A	C	A	1402
T	G	T	C	A	T	C	T	A	G	G	G	G	T	A	C	G	1402
C	A	T	C	A	T	C	T	A	G	G	G	G	C	G	C	G	1502
C	A	T	C	A	T	C	T	A	G	G	G	G	C	A	C	A	1502
T	G	C	C	A	T	C	C	A	G	G	G	G	C	G	C	G	1502
T	G	T	T	A	T	C	T	A	G	G	G	A	T	A	C	G	1505

Without impute, *HLA-C*, Illumina550K

T	C	G	C	A	C	G	A	A	A	A	G	C	102
T	C	G	C	A	A	G	A	A	A	A	G	C	102
T	C	G	C	A	C	G	A	A	G	A	G	C	102

T	C	G	C	G	C	G	A	A	A	A	G	C	102
C	C	G	C	G	A	T	G	A	A	G	G	T	102
T	C	G	C	A	C	G	A	A	A	G	G	C	102
C	T	G	T	G	A	G	G	A	A	G	G	C	102
T	C	G	C	A	C	G	A	A	A	A	G	C	103
C	C	G	T	A	A	G	A	A	G	G	A	C	202
C	C	G	C	G	A	T	G	A	A	G	G	T	302
T	C	G	C	G	A	T	G	A	G	G	G	T	302
C	C	G	C	G	A	T	G	A	G	G	G	T	302
T	C	G	C	G	A	T	G	A	A	G	G	T	303
T	C	G	C	A	A	T	G	A	A	G	G	T	303
T	C	G	T	G	A	T	G	A	A	G	G	T	303
T	C	G	C	G	A	T	G	A	A	G	G	C	303
T	C	G	C	A	A	T	G	A	A	G	G	C	304
T	C	G	C	G	A	T	G	A	A	G	G	T	304
T	C	G	C	A	A	T	G	A	A	G	G	T	304
C	T	G	C	A	A	G	A	G	G	G	A	T	401
C	T	G	C	G	A	G	A	G	G	G	A	T	401
T	C	G	C	A	A	G	A	G	G	G	A	T	401
T	C	G	C	G	A	G	A	G	G	G	A	C	403
T	C	G	C	G	A	G	A	A	G	G	G	C	602
C	C	A	C	A	C	G	G	A	A	G	G	C	701
C	C	A	C	G	A	G	G	A	G	G	A	C	702

C	C	A	C	A	A	G	G	A	G	G	A	C	702
C	T	A	C	G	A	G	G	A	G	G	A	C	702
C	C	A	C	G	A	G	G	A	G	G	A	T	702
T	C	G	C	A	A	T	G	A	A	G	G	C	702
C	C	A	C	A	C	G	G	A	A	G	G	C	702
C	C	G	T	G	A	T	G	A	A	G	G	T	702
C	C	A	C	G	A	G	G	A	A	A	G	C	702
C	C	G	T	G	A	G	G	A	A	G	G	C	704
C	T	A	C	G	A	G	G	A	A	G	G	C	704
T	C	G	T	A	A	G	G	A	A	G	G	C	801
C	C	A	C	G	A	G	G	A	G	G	A	C	801
C	C	G	T	A	A	G	G	A	A	G	G	C	801
T	C	G	T	A	A	G	G	A	G	G	G	C	801
T	T	G	T	A	A	G	G	A	A	G	G	C	801
T	C	G	T	A	A	G	A	A	G	G	G	C	1202
C	C	G	T	A	A	G	A	A	G	G	A	T	1202
T	C	G	C	G	A	T	G	A	A	G	G	C	1202
T	C	G	C	A	A	G	A	A	G	G	G	C	1203
T	C	G	C	A	A	G	A	A	A	G	G	T	1402
C	T	G	C	A	C	G	A	A	A	G	G	C	1502
T	C	G	C	A	C	G	A	A	A	G	G	C	1502
T	C	G	C	G	A	T	G	A	A	G	G	T	1505

Without impute, *HLA-C*, Union

G	C	C	C	T	C	A	G	A	A	T	T	C	A	T	A	A	A	102
G	C	T	T	T	T	A	G	A	A	T	T	C	A	T	A	A	A	102
G	C	C	C	T	C	G	G	A	A	T	T	C	A	T	A	A	A	102
G	C	C	C	T	C	A	G	A	A	T	T	C	A	T	A	G	A	102
G	C	T	T	T	T	A	G	A	A	T	T	C	A	T	A	G	A	102
G	C	T	C	T	C	G	G	G	A	T	T	T	G	T	G	A	A	102
G	C	T	C	T	T	A	G	A	A	T	T	T	G	T	G	A	A	102
G	C	T	C	T	T	G	T	G	A	T	T	T	G	T	A	G	A	102
G	C	C	C	T	C	A	G	A	A	T	T	C	A	T	A	A	A	103
G	C	T	C	T	C	A	G	A	A	T	T	C	G	T	A	A	A	202
A	T	T	C	T	T	G	T	G	A	T	T	T	G	T	A	G	A	302
G	C	T	T	T	T	G	T	G	A	T	T	T	G	T	A	G	A	303
G	C	T	C	T	T	G	T	G	A	T	T	T	G	T	A	G	A	303
G	C	T	T	T	T	G	T	G	A	T	T	T	G	T	A	A	A	303
G	C	T	T	T	T	A	T	G	A	T	T	T	G	T	A	A	A	303
G	C	T	T	T	T	A	T	G	A	T	T	T	G	T	A	A	T	304
G	C	T	T	T	T	G	T	G	A	T	T	T	G	T	A	G	A	304
G	C	T	T	T	T	A	T	G	A	T	T	T	G	T	A	G	A	304
G	C	T	T	T	T	A	T	G	A	T	T	T	G	T	G	A	A	304
A	T	T	C	T	T	A	G	A	G	T	T	C	G	C	A	G	A	401
A	T	T	C	T	T	G	G	A	G	T	T	C	G	C	A	G	A	401
G	C	T	C	T	T	A	G	A	G	T	T	C	G	C	A	G	A	401

A	T	T	C	T	T	A	G	A	G	T	T	C	G	T	A	G	A	401
G	C	T	C	T	T	G	G	A	G	T	T	C	G	T	A	A	A	403
G	C	T	C	T	T	G	G	A	G	T	G	C	G	T	A	A	A	403
G	C	C	C	C	T	G	G	A	A	C	T	C	G	T	A	A	A	602
G	C	T	C	T	C	A	G	G	A	T	T	T	G	T	A	A	A	701
G	C	T	C	T	C	G	G	G	A	T	G	C	G	T	A	A	A	702
G	C	T	C	T	C	A	G	G	A	T	G	C	G	T	A	A	A	702
G	C	T	T	T	T	A	T	G	A	T	T	T	G	T	G	A	A	702
G	C	T	C	T	C	A	G	G	A	T	T	T	G	T	A	A	A	702
G	C	T	C	T	C	G	G	G	A	T	T	C	A	T	A	A	A	702
A	T	T	C	T	T	G	T	G	A	T	T	T	G	T	A	G	A	702
G	C	T	C	T	C	G	G	G	A	T	T	T	G	C	G	A	A	704
G	C	T	C	T	T	A	G	G	A	T	T	T	G	T	G	A	A	801
G	C	T	C	T	C	G	G	G	A	T	G	C	G	T	A	A	A	801
G	C	T	C	T	T	A	G	A	A	C	T	C	G	T	A	G	A	1202
G	C	T	C	T	T	A	G	A	A	C	T	C	G	C	A	G	A	1202
G	C	T	T	T	T	G	T	G	A	T	T	T	G	T	A	A	A	1202
G	T	T	C	C	T	A	G	A	A	C	T	C	G	T	A	A	A	1203
G	C	C	C	T	C	A	G	A	A	C	T	C	G	T	A	A	A	1203
G	C	T	C	T	T	A	G	A	A	T	T	C	G	T	A	G	A	1402
G	C	T	C	T	T	A	G	A	A	T	T	C	G	T	A	A	A	1402
G	C	T	C	T	C	A	G	A	A	T	T	T	G	T	G	A	A	1502
G	C	T	C	T	C	A	G	A	A	T	T	T	G	T	A	A	A	1502

G	C	C	C	T	C	A	G	A	A	T	T	T	G	T	G	A	A	1502
G	C	T	T	T	T	G	T	G	A	T	T	T	G	T	A	G	A	1505

Without impute, *HLA-DPB1*, Affy5.0

A	C	T	G	C	T	G	C	T	G	C	G	G	A	C	101
T	T	T	G	T	C	G	C	T	A	C	A	G	G	C	102
T	T	C	G	T	C	G	C	C	G	C	G	A	A	C	102
T	T	C	A	C	T	A	T	C	G	C	G	A	A	C	201
T	T	C	G	T	C	G	T	C	G	C	G	A	A	C	201
T	T	C	A	C	T	A	T	C	G	C	G	A	G	C	201
T	T	T	A	C	T	A	T	C	G	C	G	A	A	C	201
A	C	C	A	C	T	A	T	C	G	C	G	A	A	C	201
T	T	T	G	T	C	G	T	C	G	C	G	A	A	C	201
T	T	C	A	C	T	G	T	C	G	C	G	A	A	C	201
T	T	T	G	T	T	A	T	C	G	C	G	A	A	C	201
T	T	T	A	C	T	G	T	C	G	C	G	A	A	C	201
T	T	T	G	T	C	A	T	C	G	C	G	A	A	C	201
T	C	C	A	C	T	A	T	C	G	C	G	A	A	C	201
T	T	T	G	T	T	G	T	C	G	C	G	A	A	C	201
A	C	T	G	T	T	A	T	C	G	C	G	A	A	C	201
A	C	C	G	T	C	G	C	C	G	C	G	A	A	C	202
A	C	T	G	T	C	G	C	C	G	C	G	A	A	C	202
T	T	C	G	T	C	G	C	C	G	C	G	A	A	C	202

T	T	T	G	C	T	A	C	C	G	C	G	A	A	C	202
T	T	C	G	T	T	A	C	C	G	C	G	A	A	C	202
T	T	T	G	T	T	A	T	C	G	C	G	A	A	C	202
T	T	T	G	T	C	G	C	C	G	C	G	A	A	C	202
T	T	C	A	C	T	A	T	C	G	C	G	A	A	C	202
A	C	C	G	T	C	G	C	C	G	C	G	A	A	T	202
T	T	C	A	C	T	G	T	C	G	C	G	A	A	C	401
A	C	T	A	C	T	G	T	C	G	C	G	A	A	T	401
T	T	T	A	C	T	G	T	C	G	C	G	A	A	C	401
T	T	C	A	C	T	G	T	C	G	C	G	A	A	T	401
A	T	T	G	C	T	A	T	C	G	C	G	A	A	C	401
A	C	T	A	C	T	A	T	C	G	C	G	A	A	C	402
T	T	T	G	T	T	G	T	C	G	C	G	A	A	C	402
T	T	T	G	T	C	G	C	T	A	C	A	G	G	C	501
T	T	C	G	T	C	G	C	T	A	C	A	G	G	C	501
T	T	T	G	T	C	G	C	T	A	T	G	A	A	C	501
T	T	C	G	T	T	G	C	T	A	C	A	G	G	C	501
T	C	C	G	T	C	G	C	T	A	C	A	G	G	C	501
T	T	T	G	C	T	A	C	T	A	C	A	G	G	C	501
T	T	T	G	T	C	G	C	T	A	C	G	G	A	C	501
T	T	C	A	C	T	G	C	T	A	C	A	G	G	C	501
T	T	T	G	C	T	G	C	T	A	C	A	G	G	C	501
T	T	T	A	C	T	A	C	T	A	C	A	G	G	C	501

A	C	T	G	T	T	G	C	T	A	C	A	G	G	C	501
A	C	T	G	T	C	A	C	T	A	C	A	G	G	C	501
T	T	T	G	C	T	A	T	T	A	C	A	G	A	T	501
T	T	T	G	T	T	A	C	T	A	C	A	G	G	C	501
T	T	C	G	T	T	A	C	T	A	C	A	G	G	C	501
T	T	C	G	T	C	G	C	T	A	C	G	A	A	C	501
A	C	C	G	T	C	G	C	T	A	C	G	G	A	C	501
T	T	C	G	C	T	G	C	T	A	C	G	G	A	T	901
T	T	T	G	T	T	G	C	T	A	T	G	A	A	C	902
A	C	T	G	C	C	G	C	T	A	T	G	A	A	C	902
A	T	T	G	C	C	G	C	T	A	T	G	A	A	C	902
A	C	C	G	T	C	G	C	T	A	T	G	A	A	C	902
A	T	T	G	T	T	G	C	T	A	T	G	A	A	C	902
T	T	C	G	T	C	G	T	C	G	C	G	A	A	C	902
T	T	T	G	T	C	A	C	T	A	T	G	A	A	C	1301
T	T	C	G	C	C	A	C	T	A	T	G	A	A	C	1301
A	C	T	G	C	C	G	C	T	A	T	G	A	A	C	1301
T	T	C	G	C	C	G	C	T	A	T	G	A	A	T	1301
T	T	C	G	T	T	G	C	T	A	T	G	A	A	C	1301
T	T	C	G	T	C	G	C	T	A	T	G	A	A	C	1301
T	T	T	G	T	C	G	C	T	A	T	G	A	A	C	1301
A	C	T	G	C	C	A	C	T	A	T	G	A	A	C	1301
A	T	C	G	T	T	G	C	T	A	T	G	A	A	C	1301

A	C	T	G	T	T	G	C	T	A	T	G	A	A	C	1301
T	T	T	G	C	T	G	C	T	A	C	G	G	A	C	1401
T	T	T	G	C	T	G	C	T	A	C	G	G	A	T	1401
T	T	C	G	C	T	G	C	T	A	C	G	G	A	C	1401
T	T	C	A	C	T	G	C	T	A	C	G	G	A	C	1401
T	T	T	G	C	T	G	T	C	G	C	G	A	A	T	1701
T	T	T	G	C	T	A	T	T	A	C	A	G	A	T	1901
T	T	T	G	T	C	G	C	T	A	T	G	A	A	C	1901
A	C	T	G	C	T	A	T	T	A	C	A	G	A	T	1901
A	C	T	A	C	T	G	C	T	A	C	G	G	A	C	2101
A	C	C	A	C	T	G	C	T	A	C	G	G	A	C	2101
T	T	T	G	C	T	G	C	T	A	T	G	A	A	C	2101
T	T	T	G	T	T	A	T	C	G	C	G	A	G	C	3101
T	T	T	A	C	T	A	T	C	G	C	G	A	A	C	4801

Without impute, *HLA-DPB1*, Affy6.0

T	C	A	G	T	C	C	T	C	C	T	G	A	T	A	101
T	T	A	G	T	T	C	T	C	C	T	A	C	T	G	102
T	T	A	G	T	T	C	T	C	T	C	G	A	T	A	102
G	T	C	A	C	C	C	T	T	T	C	G	A	T	A	201
T	T	C	A	C	C	C	T	T	T	C	G	A	T	A	201
G	T	A	G	T	T	C	T	T	T	C	G	A	T	A	201
T	T	A	G	T	T	C	T	T	T	C	G	A	T	A	201

G	T	C	A	C	C	C	T	T	T	C	G	A	T	G	201
T	T	A	A	C	C	C	T	T	T	C	G	A	T	A	201
G	T	A	A	T	C	C	T	T	T	C	G	A	T	A	201
G	C	C	A	C	C	C	T	T	T	C	G	A	T	A	201
G	T	C	G	T	T	C	T	T	T	C	G	A	T	A	201
T	C	C	G	T	T	C	T	T	T	C	G	A	T	A	201
G	C	A	G	T	T	C	T	C	T	C	G	A	T	A	202
T	T	A	G	T	C	C	T	C	T	C	G	A	T	A	202
T	T	A	G	T	T	C	T	C	T	C	G	A	T	A	202
G	T	A	G	T	T	C	T	C	T	C	G	A	T	A	202
T	T	C	A	C	C	C	T	T	T	C	G	A	T	A	202
G	T	A	G	T	T	C	T	C	T	C	G	A	T	G	202
G	T	A	G	T	T	C	T	T	T	C	G	A	T	A	202
T	T	A	A	T	C	C	T	T	T	C	G	A	T	A	401
T	C	A	A	T	C	C	T	T	T	C	G	A	T	A	401
T	T	A	A	C	C	C	T	T	T	C	G	A	T	A	401
G	C	A	G	T	C	C	T	T	T	C	G	A	T	A	401
T	C	C	A	T	C	C	T	T	T	C	G	A	C	A	402
G	T	A	G	T	T	C	T	T	T	C	G	A	C	A	402
T	T	A	G	T	T	C	T	C	C	T	A	C	T	G	501
G	T	A	G	T	T	C	T	C	C	T	A	C	T	G	501
T	T	A	G	T	T	C	T	C	C	T	A	A	T	A	501
G	T	C	G	T	T	C	T	C	C	T	A	C	T	G	501

T	T	C	G	T	T	C	T	C	C	T	A	C	T	G	501
T	T	A	G	T	T	C	T	C	C	T	A	C	T	A	501
G	T	A	G	T	C	C	T	C	C	T	A	C	T	G	501
G	C	A	G	T	T	C	T	C	C	T	A	C	T	G	501
T	C	A	G	T	T	C	T	C	C	T	A	C	T	G	501
G	T	A	G	T	T	C	T	C	C	T	A	A	T	A	501
G	T	C	A	C	C	C	T	C	C	T	A	C	T	G	501
T	T	A	A	T	C	C	T	C	C	T	A	C	T	G	501
T	T	A	G	T	C	C	T	T	C	T	A	C	T	A	501
G	C	A	G	T	T	C	T	C	C	T	A	C	T	A	501
G	T	A	G	T	C	T	A	C	C	T	A	C	T	A	901
G	T	A	G	T	T	C	A	C	C	T	A	A	T	A	902
T	C	A	G	T	T	C	A	C	C	T	A	A	T	A	902
T	T	A	G	T	T	C	T	C	C	T	A	C	T	G	902
G	C	A	G	T	T	C	A	C	C	T	A	A	T	A	902
T	C	A	G	T	C	C	A	C	C	T	A	A	T	A	902
T	C	A	G	C	T	C	A	C	C	T	A	A	T	A	902
T	C	C	G	T	T	C	A	C	C	T	A	A	T	A	902
T	T	A	G	T	T	C	A	C	C	T	A	A	T	A	1301
G	T	C	G	T	C	C	A	C	C	T	A	A	T	A	1301
T	C	A	G	T	C	C	A	C	C	T	A	A	T	A	1301
G	C	A	G	T	T	C	A	C	C	T	A	A	T	A	1301
G	T	A	G	T	T	C	A	C	C	T	A	A	T	A	1301

G	T	A	G	T	C	C	T	C	C	T	A	A	T	A	1301
T	C	C	A	T	T	C	A	C	C	T	A	A	T	A	1301
T	C	A	G	T	T	C	A	C	C	T	A	A	T	A	1301
T	T	A	G	T	C	T	A	C	C	T	A	C	T	A	1401
G	T	C	G	T	C	T	A	C	C	T	A	C	T	A	1401
G	T	A	G	T	C	C	T	T	T	C	G	A	C	A	1701
T	T	A	G	T	C	C	T	T	C	T	A	C	T	A	1901
G	T	A	G	T	T	C	T	C	C	T	A	A	T	A	1901
T	C	A	G	T	C	C	T	T	C	T	A	C	T	A	1901
G	C	A	A	T	C	C	A	C	C	T	A	C	T	A	2101
T	T	A	A	T	C	C	A	C	C	T	A	A	T	A	2101
T	T	A	G	T	T	C	T	T	T	C	G	A	T	G	3101
T	T	C	A	C	C	C	T	T	T	C	G	A	T	A	4801

Without impute, *HLA-DPB1*, Illumina550K

T	C	C	G	G	G	A	T	G	G	G	G	G	C	101
C	T	T	G	A	G	G	T	A	G	G	A	G	T	102
C	T	T	G	A	G	A	C	G	G	T	G	G	T	102
C	C	C	A	A	A	A	C	G	G	T	G	G	C	201
C	C	T	A	A	A	A	C	G	G	T	G	G	T	201
C	C	C	G	A	G	A	C	G	G	T	G	G	T	201
C	C	C	A	A	A	A	C	G	G	T	G	G	T	201
T	T	T	A	A	A	A	C	G	G	T	G	G	T	201

T	T	T	G	A	A	A	C	G	G	T	G	G	T	201
C	T	T	A	A	A	A	C	G	G	T	G	G	C	201
C	T	T	A	A	A	A	C	G	G	T	G	G	T	201
C	C	C	A	A	G	A	C	G	G	T	G	G	C	201
T	C	T	A	A	A	A	C	G	G	T	G	G	T	201
C	C	T	A	A	A	A	C	G	G	T	G	G	C	201
C	T	T	G	A	G	A	C	G	G	T	G	G	T	201
C	T	T	G	A	G	A	C	G	G	T	G	G	C	201
C	C	T	A	A	G	A	C	G	G	T	G	G	C	201
T	C	C	G	A	A	A	C	G	G	T	G	G	T	201
C	C	C	A	A	G	A	C	G	G	T	G	A	C	201
C	C	T	G	A	A	A	C	G	G	T	G	G	C	201
C	C	T	G	A	G	A	C	G	G	T	G	G	C	202
T	C	T	G	A	G	A	C	G	G	T	G	G	C	202
C	T	T	G	A	A	A	C	G	G	T	G	G	C	202
C	C	T	G	A	G	A	C	G	G	T	G	G	T	202
C	C	C	G	A	G	A	C	G	G	T	G	G	C	202
C	T	T	G	A	G	A	C	G	G	T	G	G	C	202
C	C	C	A	A	G	A	C	G	G	T	G	G	T	202
T	C	C	G	A	A	A	C	G	G	T	G	G	C	202
T	T	T	A	A	A	A	C	G	G	T	G	G	T	202
T	C	T	G	A	A	A	C	G	G	T	G	G	T	202
C	C	C	A	A	G	A	C	G	G	T	G	A	C	401

C	T	T	A	A	G	A	C	G	G	T	G	A	C	401
C	C	T	A	A	G	A	C	G	G	T	G	A	C	401
T	C	T	G	A	A	A	C	G	G	T	G	A	C	401
C	T	T	A	A	A	A	C	G	T	T	G	G	T	402
T	C	T	G	A	G	A	C	G	T	T	G	G	C	402
T	T	T	A	A	A	A	C	G	T	T	G	G	C	402
C	T	T	A	A	A	A	C	G	T	T	G	G	C	402
C	T	T	G	A	G	G	T	A	G	G	A	G	T	501
C	C	C	G	A	G	G	T	A	G	G	A	G	T	501
T	T	T	G	A	G	G	T	A	G	G	A	G	T	501
C	C	T	G	A	G	G	T	A	G	G	A	G	T	501
C	T	T	G	A	G	G	T	A	G	G	A	G	C	501
C	T	T	G	A	G	G	T	G	G	T	G	G	C	501
T	C	T	G	A	G	G	T	A	G	G	A	G	T	501
T	T	T	G	A	G	G	T	A	G	G	A	G	C	501
C	T	T	G	A	A	G	T	A	G	G	A	G	T	501
T	T	T	G	A	G	G	T	G	G	T	G	G	C	501
C	C	T	G	A	A	G	T	A	G	G	A	G	T	501
C	C	T	G	A	G	G	T	A	G	G	A	G	C	501
T	T	T	G	A	A	G	T	A	G	G	A	G	T	501
T	T	T	G	A	G	G	T	G	G	G	G	G	C	501
C	T	T	G	A	G	G	T	G	G	G	G	G	C	501
C	C	C	A	A	G	G	T	A	G	G	A	G	T	501

C	T	T	G	A	G	G	T	A	G	G	G	G	T	501
C	C	C	A	A	A	G	T	A	G	G	A	G	T	501
C	C	T	A	A	G	G	T	A	G	G	A	G	T	501
C	C	C	G	G	G	G	T	A	G	G	A	G	T	501
T	C	T	G	A	G	G	T	G	G	G	G	G	T	501
C	T	T	G	A	A	G	T	A	G	G	G	G	C	501
C	T	T	G	A	G	G	T	A	G	G	G	G	C	501
T	C	C	G	G	G	G	T	G	G	G	G	G	C	901
C	C	T	A	A	G	G	T	G	G	T	G	G	C	902
T	C	T	A	A	G	G	T	G	G	T	G	G	C	902
C	C	T	G	A	G	G	T	G	G	T	G	G	C	902
T	T	T	G	A	G	G	T	A	G	G	G	G	T	902
C	C	T	A	A	A	G	T	G	G	T	G	G	C	902
C	T	T	G	A	A	G	T	G	G	T	G	G	C	1301
T	C	T	G	A	G	G	T	G	G	T	G	G	C	1301
T	T	T	G	G	G	G	T	G	G	T	G	G	C	1301
C	C	C	G	G	A	G	T	G	G	T	G	G	T	1301
C	T	T	G	A	G	G	T	G	G	T	G	G	C	1301
C	C	T	A	A	G	G	T	G	G	T	G	G	C	1301
C	C	T	G	A	G	G	T	G	G	T	G	G	C	1301
C	C	T	A	A	A	G	T	G	G	T	G	G	T	1301
C	T	T	G	A	A	G	T	G	G	T	G	G	T	1301
T	C	C	G	G	G	G	T	G	G	T	G	G	C	1301

C	T	T	G	G	G	G	T	G	G	T	G	G	C	1301
C	C	T	A	G	G	G	T	G	G	T	G	G	C	1301
T	C	T	A	A	G	G	T	G	G	T	G	G	C	1301
C	T	T	G	G	G	G	T	G	G	G	G	G	C	1401
C	C	C	G	G	G	G	T	G	G	G	G	G	C	1401
C	C	C	G	G	G	G	T	G	G	G	G	G	T	1401
T	T	T	G	G	G	A	C	G	T	T	G	G	C	1701
C	T	T	G	G	G	A	C	G	T	T	G	G	C	1701
T	T	T	G	A	A	G	T	A	G	G	G	G	C	1901
C	T	T	G	A	A	G	T	A	G	G	G	G	C	1901
T	T	T	G	A	G	G	T	G	G	T	G	G	C	1901
T	C	T	A	A	G	G	T	G	G	G	G	G	C	2101
T	T	T	G	A	G	G	T	G	G	G	G	G	C	2101
C	T	T	A	A	G	G	T	G	G	T	G	G	C	2101
C	C	T	G	A	A	A	C	G	G	T	G	G	C	3101
C	T	T	A	A	A	A	C	G	G	T	G	G	T	4801

Without impute, *HLA-DPB1*, Union

A	C	A	G	T	C	G	A	C	T	C	G	G	G	A	A	G	101
A	C	A	A	T	C	A	A	T	C	C	G	G	G	A	A	G	102
A	C	C	A	T	C	G	G	C	T	T	G	A	G	G	A	G	102
A	C	A	A	T	T	A	A	T	C	C	G	G	G	A	A	G	201
A	C	C	A	T	T	A	A	T	C	C	G	G	G	A	A	G	201

G	C	C	A	T	T	A	A	T	C	C	G	G	G	A	A	G	201
G	C	A	A	T	T	A	A	T	C	C	G	G	G	A	A	G	201
A	C	C	A	T	T	A	A	T	C	C	G	G	G	G	A	G	201
A	T	A	A	T	T	A	A	T	C	C	G	G	G	G	A	G	201
G	C	A	A	T	T	A	A	T	C	C	G	G	A	A	A	G	201
A	C	A	A	T	C	A	A	T	C	C	G	G	G	A	A	G	202
G	C	C	A	T	C	A	A	T	C	C	G	G	G	A	A	G	202
A	C	A	A	T	T	A	A	T	C	C	G	G	G	A	A	G	202
G	C	C	A	T	T	A	A	T	C	C	G	G	G	A	A	G	202
A	T	A	A	T	T	A	A	T	C	C	G	G	A	A	A	G	401
A	C	A	A	T	T	A	A	T	C	C	G	G	A	A	A	G	401
A	C	C	A	T	T	A	A	T	C	C	T	G	G	A	A	G	402
G	C	C	A	T	T	A	A	T	C	C	T	G	G	A	A	G	402
A	C	A	A	T	C	G	G	C	T	T	G	A	G	G	A	G	501
A	C	C	A	T	C	G	G	C	T	T	G	A	G	G	A	G	501
G	C	C	A	T	C	G	G	C	T	T	G	A	G	G	A	G	501
G	C	A	A	T	C	G	G	C	T	T	G	A	G	G	A	G	501
A	C	A	A	T	C	G	G	C	T	C	G	G	G	A	A	G	501
A	C	A	A	T	C	G	G	C	T	T	G	G	G	A	A	G	501
A	C	A	A	T	C	G	G	C	T	T	G	G	G	G	A	G	501
G	C	A	A	T	C	G	G	C	T	C	G	G	G	A	A	G	501
A	C	A	G	T	C	G	G	C	T	T	G	A	G	G	A	G	501
A	C	A	A	T	T	A	G	C	T	T	G	G	G	A	A	A	501

G	C	A	G	A	C	G	G	C	T	T	G	G	G	A	G	G	901
A	C	C	A	A	C	G	G	C	T	C	G	G	G	A	A	G	902
G	C	C	A	A	C	G	G	C	T	C	G	G	G	A	A	G	902
A	C	A	A	A	C	G	G	C	T	C	G	G	G	A	A	G	902
A	C	A	A	T	C	G	G	C	T	T	G	G	G	G	A	G	902
A	C	A	A	A	C	G	G	C	T	C	G	G	G	A	A	G	1301
A	C	C	A	A	C	G	G	C	T	C	G	G	G	A	A	G	1301
A	C	C	G	A	C	G	G	C	T	C	G	G	G	A	A	G	1301
G	C	A	G	A	C	G	G	C	T	C	G	G	G	A	A	G	1301
G	C	C	A	A	C	G	G	C	T	C	G	G	G	A	A	G	1301
A	C	A	G	T	C	G	G	C	T	C	G	G	G	A	A	A	1301
A	C	A	G	A	C	G	G	C	T	T	G	G	G	A	G	G	1401
A	T	A	G	A	C	G	G	C	T	C	G	G	G	A	A	G	1401
G	C	A	G	A	C	G	G	C	T	T	G	G	G	A	G	G	1401
A	C	A	G	T	T	A	A	T	C	C	T	G	G	A	A	G	1701
A	C	A	A	T	T	A	G	C	T	T	G	G	G	A	A	A	1901
G	C	A	A	T	C	G	G	C	T	C	G	G	G	A	A	G	1901
G	C	C	A	T	T	A	G	C	T	T	G	G	G	A	A	A	1901
A	C	A	A	A	C	G	G	C	T	T	G	G	G	A	A	G	2101
G	C	C	A	A	C	G	G	C	T	T	G	G	G	G	A	G	2101
A	C	A	A	T	T	A	A	T	C	C	G	G	G	G	A	G	3101
A	C	A	A	T	T	A	A	T	C	C	G	G	G	A	A	G	4801

Without impute, *HLA-DQB1*, Affy5.0

C	C	C	T	T	G	T	C	G	T	A	G	A	201
C	C	C	T	T	G	C	C	G	T	A	G	A	201
C	C	G	T	G	G	T	G	A	T	C	T	A	202
C	C	G	T	G	G	C	G	A	T	C	T	A	202
G	C	C	G	T	G	C	C	G	T	C	G	G	301
C	C	C	T	T	G	C	C	A	C	C	G	A	301
G	C	C	T	T	G	C	C	A	C	C	G	A	301
G	C	C	G	T	G	C	C	A	C	C	G	A	301
C	C	G	T	G	G	C	C	A	C	C	G	A	301
C	C	G	T	G	G	C	G	G	C	C	G	G	302
C	C	C	T	G	G	C	C	A	C	C	G	A	303
C	C	G	T	G	G	C	G	G	C	C	G	G	303
C	A	G	G	G	A	C	C	G	T	A	G	A	303
C	C	G	T	G	G	C	G	A	C	C	G	G	401
C	C	C	T	T	G	T	C	G	T	A	G	A	401
C	C	C	T	G	A	C	C	G	T	A	G	A	401
G	C	C	G	T	G	C	G	A	C	C	G	G	402
C	C	G	T	G	G	C	G	A	C	C	G	G	402
G	A	G	G	G	A	C	C	A	C	C	G	G	501
C	C	C	T	G	A	C	C	A	C	C	G	G	501
C	C	G	T	G	A	C	C	A	C	C	G	G	501
C	C	C	T	G	G	C	C	A	C	C	G	A	501

C	A	G	G	G	A	C	C	A	C	C	G	A	502
C	C	C	T	G	A	C	C	A	C	C	G	A	502
G	A	G	G	G	A	C	C	A	C	C	G	A	502
G	C	C	G	G	A	C	C	A	C	C	G	A	502
C	C	C	T	G	A	C	C	G	T	A	G	A	503
C	A	G	G	G	A	C	C	G	T	A	G	A	503
G	C	C	T	G	A	C	C	G	T	A	G	A	503
C	C	C	T	G	A	C	G	A	T	C	T	A	601
C	A	G	G	G	A	C	G	A	T	C	T	A	601
G	A	G	G	G	A	C	G	A	T	C	T	A	601
C	C	G	T	G	G	C	G	G	C	C	G	G	601
C	C	C	G	G	A	C	G	A	T	C	T	A	601
C	A	G	G	G	A	C	C	G	T	A	G	A	602
G	C	C	T	G	A	C	C	G	T	A	G	A	609
C	A	G	G	G	A	C	C	G	T	A	G	A	610

Without impute, *HLA-DQB1*, Affy6.0

A	C	T	A	G	C	C	T	T	A	T	A	201
A	T	T	A	G	C	C	T	T	A	T	A	201
A	C	T	G	G	C	C	T	T	A	T	A	201
C	T	T	G	G	C	T	C	T	A	T	A	202
A	C	T	A	G	C	T	T	C	G	C	A	301
A	T	G	A	G	C	T	T	C	A	T	G	301

A	T	G	A	G	C	T	T	C	G	C	A	301
A	T	T	G	G	C	T	T	C	G	C	A	301
A	T	T	G	G	T	T	T	T	G	C	G	302
A	C	T	G	G	T	T	T	C	G	C	A	303
A	T	T	G	G	T	T	T	T	G	C	G	303
A	T	G	G	C	C	T	T	T	A	T	A	303
A	T	T	G	G	T	T	C	C	G	C	G	401
A	C	T	G	C	C	T	T	T	A	T	A	401
A	C	T	A	G	C	C	T	T	A	T	A	401
A	T	G	A	G	C	T	C	C	G	C	G	402
A	T	T	G	G	T	T	C	C	G	C	G	402
A	T	G	G	C	C	T	T	T	A	C	G	501
A	C	T	G	C	C	T	T	T	A	C	G	501
A	T	T	G	C	C	T	T	T	A	C	G	501
A	C	T	G	G	T	T	T	C	G	C	A	501
A	T	G	G	C	C	T	T	C	G	C	A	502
A	C	T	G	C	C	T	T	C	G	C	A	502
A	T	T	G	C	C	T	T	C	G	C	A	502
A	C	T	G	C	C	T	T	T	A	T	A	503
A	T	T	A	C	C	T	T	T	A	T	A	503
A	T	G	G	C	C	T	T	T	A	T	A	503
A	T	T	G	C	C	T	C	T	A	T	A	601
A	T	G	G	C	C	T	C	T	A	T	A	601

A	T	T	G	G	T	T	T	T	G	C	G	601
A	T	G	G	C	C	T	T	T	A	T	A	602
A	T	T	G	C	C	T	T	T	A	T	A	609
A	C	T	A	C	C	T	T	T	A	T	A	609
A	T	T	G	G	C	T	T	T	A	T	A	609
A	T	G	G	C	C	T	T	T	A	T	A	610

Without impute, *HLA-DQB1*, Illumina550K

T	T	A	G	A	G	T	T	T	T	A	201
C	C	A	A	G	G	G	T	C	C	A	202
T	T	A	G	A	G	G	T	T	T	G	301
T	T	G	A	G	G	G	T	T	T	G	301
T	T	A	G	A	G	G	C	T	T	G	301
C	T	G	A	G	G	G	T	T	T	G	301
T	T	G	A	G	G	G	C	T	T	G	301
C	T	G	A	G	G	G	C	C	T	G	302
T	T	A	G	G	G	G	T	T	T	G	303
T	T	A	G	G	G	G	C	T	T	G	303
T	T	A	G	A	G	T	T	T	T	A	303
C	T	G	A	G	G	G	C	C	T	G	303
C	T	G	A	G	G	G	T	C	C	G	401
T	T	A	G	G	T	T	T	T	T	A	401
T	T	G	A	G	G	G	T	C	C	G	402

C	T	G	A	G	G	G	T	C	C	G	402
T	T	A	G	G	T	T	T	C	T	G	501
T	T	A	G	G	G	G	C	T	T	G	501
T	T	A	G	G	T	G	T	T	T	G	502
T	T	G	A	G	T	G	T	T	T	G	502
T	T	A	G	G	T	G	C	T	T	G	502
T	T	A	G	G	T	T	T	C	T	A	503
T	T	A	A	G	T	G	T	C	C	A	601
T	T	A	G	G	T	G	T	C	C	A	601
C	T	G	A	G	G	G	C	C	T	G	601
T	T	A	G	G	T	T	T	T	T	A	602
T	T	G	A	G	T	T	T	T	T	A	602
T	T	A	A	G	T	T	T	T	T	A	609
T	T	A	G	G	T	T	T	T	T	A	610

Without impute, *HLA-DQB1*, Union

G	T	C	T	C	G	T	C	T	A	T	G	A	201
G	G	C	T	C	G	G	T	C	A	T	A	A	202
G	T	C	T	C	G	G	T	T	G	C	A	G	301
G	T	C	T	C	G	G	T	T	G	C	G	G	301
G	G	C	C	C	G	G	T	T	G	C	A	G	301
G	G	C	C	T	G	G	T	C	A	T	G	G	302
G	G	C	C	T	G	G	T	T	G	C	A	G	303

G	G	C	C	T	G	G	T	C	A	T	G	G	303
G	G	C	T	C	T	T	T	T	A	T	G	A	303
G	G	C	C	T	G	G	T	C	A	C	A	G	401
T	G	A	T	C	T	T	T	T	A	T	G	A	401
G	T	C	T	C	G	T	C	T	A	T	G	A	401
G	T	C	T	C	G	G	T	C	A	C	A	G	402
G	G	C	C	T	G	G	T	C	A	C	A	G	402
G	G	C	T	C	T	T	T	C	A	T	A	G	501
T	G	A	T	C	T	T	T	C	A	T	A	G	501
G	G	C	C	T	G	G	T	T	G	C	A	G	501
G	G	C	T	C	T	G	T	T	G	C	A	G	502
T	G	A	T	C	T	G	T	T	G	C	A	G	502
T	G	A	T	C	T	T	T	C	A	T	G	A	503
G	G	C	T	C	T	T	T	C	A	T	G	A	503
G	G	A	T	C	T	G	T	C	A	T	A	A	601
G	G	C	T	C	T	G	T	C	A	T	A	A	601
G	G	C	C	T	G	G	T	C	A	T	G	G	601
G	G	C	T	C	T	T	T	T	A	T	G	A	602
G	G	A	T	C	T	T	T	T	A	T	G	A	609
G	G	C	T	C	T	T	T	T	A	T	G	A	610

Without impute, *HLA-DRB1*, Affy5.0

C	C	A	A	C	T	G	T	G	G	C	T	A	T	C	G	101

T	C	A	A	C	C	C	T	T	A	C	T	G	C	A	G	301
T	C	A	A	C	T	C	T	T	A	C	T	G	C	A	G	301
T	C	A	A	C	C	C	T	T	A	C	T	A	T	C	G	301
T	C	A	G	C	T	G	T	G	A	C	T	A	T	C	G	401
T	C	A	G	C	T	G	T	G	A	G	T	G	T	C	G	403
T	C	G	G	C	T	G	T	G	A	G	T	G	T	C	G	403
T	C	A	G	C	T	G	T	G	A	G	T	G	T	C	G	404
T	C	A	G	C	T	G	T	G	A	G	C	A	T	C	G	405
T	C	A	G	C	T	G	T	G	A	G	T	G	T	C	G	405
T	C	A	A	C	C	C	T	T	A	C	T	G	C	A	G	405
T	C	A	G	C	T	G	T	G	A	G	T	G	T	C	G	406
T	C	A	G	C	T	G	T	G	A	G	T	G	T	C	G	407
T	C	A	G	C	T	G	T	G	A	G	C	A	T	C	G	410
T	C	A	G	C	T	G	T	G	A	G	C	A	C	C	T	701
T	A	G	G	G	T	C	G	T	A	G	C	A	T	C	G	802
C	C	A	A	C	T	C	T	G	G	G	C	A	C	C	T	803
C	C	A	A	C	T	C	T	G	G	C	T	G	C	A	G	803
C	C	A	A	C	T	C	G	G	G	G	C	A	C	C	T	803
T	A	G	G	G	T	C	G	T	A	G	C	A	T	C	G	809
C	C	A	G	G	T	C	G	T	A	G	C	A	T	C	G	809
T	C	A	G	C	T	C	T	G	A	C	T	A	T	C	G	901
T	C	A	G	C	T	G	T	G	A	G	T	G	T	C	G	901
C	C	A	A	C	T	G	G	G	G	C	T	G	C	A	G	901

C	C	A	A	C	T	C	T	G	G	C	T	A	T	C	G	1001
T	C	A	G	C	T	C	T	G	A	C	T	A	T	C	G	1001
T	C	A	A	C	T	C	T	T	A	C	T	A	T	C	G	1101
T	A	G	G	G	T	C	T	T	A	C	T	A	T	C	G	1106
C	A	G	G	G	T	C	T	T	A	C	T	A	T	C	G	1201
T	C	A	A	C	T	C	T	T	A	C	T	A	T	C	G	1201
C	C	G	G	G	T	C	G	T	A	C	T	G	C	C	G	1202
C	C	G	G	G	T	C	G	T	A	C	T	A	T	C	G	1202
C	C	G	G	G	T	C	G	G	G	C	T	A	T	C	G	1202
T	A	A	G	G	T	C	G	T	A	C	T	G	C	C	G	1202
C	C	G	G	G	T	C	T	T	A	C	T	A	T	C	G	1202
C	C	G	G	G	T	C	T	G	G	C	T	A	T	C	G	1202
C	C	G	G	G	T	C	T	G	G	C	T	G	C	A	G	1301
C	C	G	G	G	T	C	T	G	G	C	T	G	C	A	G	1302
T	C	A	A	C	T	C	T	T	A	C	T	A	T	C	G	1312
T	C	G	A	C	T	C	T	T	A	C	T	A	T	C	G	1312
T	C	A	A	C	T	C	T	G	G	C	T	A	T	C	G	1401
T	C	A	A	C	T	C	T	G	G	C	T	G	C	A	G	1401
C	A	G	G	G	C	C	T	T	A	C	T	A	T	C	G	1403
T	C	A	A	C	T	C	T	G	G	C	T	G	C	A	G	1404
T	A	G	G	G	T	C	T	G	G	C	T	G	C	A	G	1404
T	C	A	A	C	T	C	T	G	G	C	T	G	C	A	G	1405
T	A	G	G	G	T	C	T	G	G	C	T	G	C	A	G	1410

T	C	A	A	C	T	C	T	G	G	C	T	G	C	A	G	1418
C	C	A	A	C	T	G	G	G	G	C	T	G	C	A	G	1501
C	C	A	A	C	T	G	G	G	G	G	C	A	C	C	T	1501
C	C	A	A	C	T	G	G	G	G	C	T	A	T	C	G	1501
T	A	G	G	G	T	G	G	G	G	C	T	A	T	C	G	1502
C	A	G	G	G	T	G	G	G	G	C	T	A	T	C	G	1502
C	A	G	G	G	T	G	G	G	G	G	C	A	C	C	T	1502
C	C	A	A	C	T	G	G	G	G	C	T	A	T	C	G	1602
T	C	A	A	C	T	G	G	G	G	C	T	A	T	C	G	1602

Without impute, *HLA-DRB1*, Affy6.0

A	C	C	C	A	G	C	T	T	G	G	C	C	T	A	101
A	C	C	C	A	G	C	T	T	A	G	C	C	T	A	101
A	T	T	C	A	C	T	C	T	A	G	T	C	C	A	301
A	T	T	C	A	C	T	C	T	A	G	C	C	T	G	301
G	C	C	C	A	G	C	T	A	A	A	C	C	T	G	401
G	C	C	C	A	G	C	T	A	A	A	C	T	T	A	403
G	C	C	C	A	G	C	T	A	A	A	C	T	T	A	404
G	C	C	C	A	G	C	T	T	A	A	C	T	T	A	405
G	C	C	C	A	G	C	T	A	A	A	C	T	T	A	405
A	T	T	C	A	C	T	C	T	A	G	T	C	C	A	405
G	C	C	C	A	G	C	T	A	A	A	C	T	T	A	406
G	C	C	C	A	G	C	T	A	A	A	C	T	T	A	407

G	C	C	C	A	G	C	T	T	A	A	C	T	T	A	410
G	C	C	C	C	G	C	T	T	A	A	C	C	T	A	701
G	T	C	C	A	C	C	T	T	G	A	C	C	T	A	802
A	T	T	T	A	C	C	T	T	A	G	C	C	T	A	803
A	C	C	C	A	C	T	C	T	A	G	C	C	T	A	803
G	T	C	C	A	C	C	T	T	G	A	C	C	T	A	809
G	C	C	C	A	C	T	C	T	A	A	C	T	T	G	901
G	C	C	C	A	G	C	T	A	A	A	C	T	T	A	901
A	T	T	C	A	G	C	T	T	G	A	C	C	T	A	901
A	C	C	C	A	C	T	C	T	A	G	C	C	T	A	1001
G	C	C	C	A	C	T	C	T	A	A	C	T	T	G	1001
A	T	T	C	A	C	T	C	T	A	G	C	C	T	G	1101
G	T	C	C	A	C	T	C	T	A	G	C	C	T	G	1106
G	T	C	C	A	C	T	C	T	A	G	C	C	T	G	1201
A	T	T	C	A	C	T	C	T	A	G	C	C	T	G	1201
G	T	C	C	A	C	T	C	T	A	G	T	C	T	G	1201
G	T	C	T	A	C	C	T	T	G	A	C	C	T	G	1202
G	T	C	T	A	C	C	T	T	A	G	C	C	T	A	1301
G	T	C	T	A	C	C	T	T	A	G	C	C	T	A	1302
A	T	T	C	A	C	T	C	T	A	G	T	C	T	G	1312
A	T	T	C	A	C	C	C	T	A	G	C	C	T	G	1401
A	T	T	C	A	C	C	C	T	A	G	C	C	T	A	1401
G	T	C	C	A	C	T	C	T	A	G	T	C	T	G	1403

A	T	T	C	A	C	C	C	T	A	G	C	C	T	A	1404
A	T	T	C	A	C	C	C	T	A	G	C	C	T	A	1405
G	T	C	C	A	C	C	C	T	A	G	C	C	T	A	1410
A	T	T	C	A	C	C	C	T	A	G	C	C	T	A	1418
A	T	T	C	A	G	C	T	T	G	A	C	C	T	A	1501
A	T	T	C	A	G	C	T	T	G	A	C	C	T	G	1501
G	T	C	C	A	G	C	T	T	G	A	C	C	T	A	1502
G	T	C	C	A	G	C	T	T	G	A	C	C	T	G	1502
A	T	T	C	A	G	C	T	T	A	A	C	C	T	G	1602

Without impute, *HLA-DRB1*, Illumina550K

G	C	A	C	T	A	A	A	G	A	C	T	T	G	G	T	C	C	101
G	C	G	C	C	A	A	A	A	C	T	T	T	G	A	G	T	C	301
G	C	G	C	C	G	A	A	A	C	T	T	T	G	A	G	T	C	301
G	C	G	C	C	A	A	A	A	C	C	G	T	G	G	T	T	A	301
G	C	G	C	T	A	G	A	G	C	C	G	T	G	G	T	T	A	401
G	C	G	C	T	A	G	A	G	C	C	G	C	T	G	T	T	A	403
A	C	G	C	T	A	G	A	G	C	C	G	C	T	G	T	T	A	403
G	C	G	C	T	A	G	A	G	C	C	G	C	G	G	T	T	A	404
G	C	G	C	T	A	G	A	G	C	C	G	C	T	G	T	T	A	404
A	A	G	C	T	G	G	A	G	C	C	G	T	G	G	T	C	C	405
G	A	G	C	T	G	G	A	G	C	C	G	T	G	G	T	C	C	405
G	C	G	C	T	A	G	A	G	C	C	G	C	T	G	T	T	A	405

G	A	G	C	T	G	G	A	G	C	C	G	T	G	G	T	C	A	405
A	A	G	C	T	G	G	A	G	C	C	G	C	T	G	T	T	A	405
G	C	G	C	T	G	G	A	G	C	C	G	T	G	G	T	C	C	405
G	C	G	C	C	A	A	A	A	C	T	T	T	G	A	G	T	C	405
G	A	G	T	C	A	A	G	G	C	C	T	T	G	A	G	T	C	405
G	C	G	C	T	A	G	A	G	C	C	G	C	T	G	T	T	A	406
G	C	G	C	T	A	G	A	G	C	C	G	C	T	G	T	T	A	407
G	C	G	C	T	G	G	A	G	C	C	G	T	G	G	T	C	A	410
G	C	G	C	T	A	A	A	G	C	C	G	T	G	G	T	T	A	701
G	A	G	C	T	A	A	A	G	C	C	G	T	G	G	T	T	A	701
G	A	G	T	T	A	G	A	G	C	C	G	T	G	G	T	C	A	802
G	C	G	T	C	A	A	A	G	A	C	G	T	G	G	T	T	A	803
A	C	A	C	T	A	A	A	G	A	C	T	T	G	A	G	T	A	803
G	A	G	T	T	A	G	A	G	C	C	G	T	G	G	T	C	A	809
A	C	G	C	T	G	A	A	G	C	C	G	T	G	G	T	T	A	901
G	C	G	C	T	G	A	A	G	C	C	G	T	G	G	T	T	A	901
G	C	G	C	T	G	A	A	G	C	C	G	C	G	G	T	T	A	901
A	C	G	C	T	G	A	A	G	C	C	G	C	G	G	T	T	A	901
G	A	G	C	T	G	A	A	G	C	C	G	T	G	G	T	T	A	901
G	C	G	C	T	A	G	A	G	C	C	G	C	T	G	T	T	A	901
G	A	G	C	T	G	A	A	G	C	C	G	C	G	G	T	T	A	901
G	C	G	C	C	A	A	A	A	C	T	T	T	G	A	G	T	C	901
G	A	G	T	C	A	A	G	G	C	C	T	T	G	A	G	T	C	901

A	C	A	C	T	A	A	A	G	A	C	T	T	G	G	T	C	A	1001
A	C	G	C	T	G	A	A	G	C	C	G	C	G	G	T	T	A	1001
G	C	G	C	C	G	A	A	A	C	C	G	T	G	G	T	T	A	1101
G	C	G	C	C	G	A	A	A	C	C	G	C	G	G	T	T	A	1101
A	A	G	T	T	G	A	A	A	C	C	G	T	G	G	T	T	A	1106
G	C	G	T	T	G	A	A	A	C	C	G	T	G	G	T	T	A	1201
G	A	G	T	T	G	A	A	A	C	C	G	T	G	G	T	T	A	1201
G	C	G	C	C	G	A	A	A	C	C	G	C	G	G	T	T	A	1201
A	A	G	T	T	G	A	A	A	C	C	G	T	G	G	T	T	A	1201
G	C	G	T	T	G	A	A	A	C	C	G	T	G	G	T	T	C	1201
G	A	G	T	T	G	A	A	A	C	T	G	T	G	G	T	T	A	1201
G	C	G	T	T	A	G	A	G	C	C	G	T	T	G	T	T	A	1202
G	C	G	T	T	A	G	A	G	C	C	G	T	G	G	T	T	A	1202
G	C	G	T	T	A	G	A	G	C	C	G	C	G	G	T	T	A	1202
G	A	G	T	T	A	A	A	G	A	C	T	T	G	A	G	T	C	1301
G	A	G	T	T	A	A	A	G	A	C	T	T	G	A	G	T	C	1302
G	C	G	C	C	G	A	A	A	C	T	G	T	G	G	T	T	A	1312
G	C	G	C	C	G	A	A	A	C	T	G	C	G	G	T	T	A	1312
A	C	G	C	C	A	A	A	G	A	C	G	T	G	G	T	T	A	1401
G	C	G	C	C	A	A	A	G	A	C	T	T	G	A	G	T	A	1401
G	C	G	C	C	A	A	A	G	A	C	G	T	G	G	T	T	A	1401
G	C	G	T	C	G	A	A	A	C	T	G	T	G	G	T	T	A	1403
G	C	G	C	C	A	A	A	G	A	C	T	T	G	A	G	T	A	1404

G	C	G	C	C	A	A	A	G	A	C	T	T	G	A	G	T	C	1405
G	C	G	C	C	A	A	A	G	A	C	T	T	G	A	G	T	A	1405
G	A	G	T	T	A	A	A	G	A	C	T	T	G	A	G	T	C	1410
G	C	G	C	C	A	A	A	G	A	C	T	T	G	A	G	T	C	1418
G	A	G	T	C	A	A	G	G	C	C	G	T	G	G	T	T	A	1501
G	A	G	T	C	A	A	G	G	C	C	T	T	G	A	G	T	C	1501
G	A	G	T	C	A	A	G	G	C	C	T	T	G	A	G	T	A	1501
G	A	G	T	T	A	A	G	G	C	C	T	T	G	G	T	C	A	1502
A	A	G	T	T	A	A	G	G	C	C	G	T	G	G	T	T	A	1502
G	C	G	T	T	A	A	G	G	C	C	G	T	G	G	T	T	A	1502
G	A	G	T	C	A	A	A	G	C	C	G	T	G	G	T	T	A	1602
G	C	G	T	C	A	A	A	G	C	C	G	T	G	G	T	T	A	1602
G	A	G	T	C	A	A	A	G	C	C	G	C	G	G	T	T	A	1602
G	C	G	C	C	A	A	A	G	C	C	G	C	G	G	T	T	A	1602

Without impute, *HLA-DRB1*, Union

G	G	C	T	A	C	C	T	T	T	G	A	C	T	T	A	C	C	T	G	T	C	101
G	G	T	C	A	C	T	C	T	T	C	A	T	C	T	A	T	C	T	A	C	C	301
G	G	T	C	A	C	T	T	T	T	C	A	T	C	T	A	T	C	T	A	C	C	301
G	G	T	C	A	C	T	C	T	T	C	A	T	C	T	A	C	C	G	G	T	A	301
A	G	T	T	G	C	C	T	T	C	G	G	C	T	A	A	C	C	G	A	T	C	401
A	G	T	T	G	C	C	T	T	C	G	G	C	T	A	A	C	T	G	A	T	C	403
G	A	T	T	G	C	C	T	T	C	G	G	C	T	A	A	C	T	G	A	T	C	403

A	G	T	T	G	C	C	T	T	C	G	G	C	T	A	A	C	T	G	A	T	C	404
G	G	T	T	G	C	C	T	T	C	G	G	C	T	T	A	C	T	G	A	T	C	405
A	G	T	T	G	C	C	T	T	C	G	G	C	T	A	A	C	T	G	A	T	C	405
G	G	T	C	A	C	T	C	T	T	C	A	T	C	T	A	T	C	T	A	C	C	405
A	G	T	T	G	C	C	T	T	C	G	G	C	T	A	A	C	T	G	A	T	C	406
A	G	T	T	G	C	C	T	T	C	G	G	C	T	A	A	C	T	G	A	T	C	407
G	A	T	T	G	C	C	T	T	C	G	G	C	T	T	A	C	T	G	A	T	C	410
G	G	T	T	G	C	C	T	T	C	G	A	C	T	T	A	C	C	G	A	T	C	701
G	G	T	T	G	C	C	T	T	C	G	A	C	T	T	A	C	C	G	A	T	A	701
G	G	T	T	G	G	C	T	T	T	C	G	C	T	T	A	C	C	G	A	T	C	802
G	G	C	C	A	C	T	T	C	T	C	A	C	T	T	A	C	C	G	A	T	C	803
G	G	C	C	A	C	T	T	C	T	C	A	C	T	T	A	C	C	G	A	T	A	803
G	G	C	T	A	C	C	T	T	T	C	A	T	C	T	A	C	C	T	A	T	A	803
G	G	T	T	G	G	C	T	T	T	C	G	C	T	T	A	C	C	G	A	T	C	809
G	A	T	T	G	C	C	T	T	T	C	A	T	C	T	A	C	T	G	A	T	A	901
G	G	T	T	G	C	C	T	T	T	C	A	T	C	T	A	C	T	G	A	T	A	901
A	G	T	T	G	C	C	T	T	C	G	G	C	T	A	A	C	T	G	A	T	C	901
G	G	C	C	A	C	T	T	T	T	G	A	C	T	T	G	C	C	T	A	T	C	901
G	G	C	T	A	C	C	T	T	T	C	A	T	C	T	A	C	C	T	G	T	A	1001
G	A	T	T	G	C	C	T	T	T	C	A	T	C	T	A	C	T	G	A	T	A	1001
G	G	T	C	A	C	T	T	T	T	C	A	T	C	T	A	C	C	G	A	T	A	1101
G	G	T	T	G	G	C	T	T	T	C	A	T	C	T	A	C	C	G	A	T	A	1106
G	G	C	T	G	G	C	T	T	T	C	A	T	C	T	A	C	C	G	A	T	A	1201

G	G	T	T	G	G	C	T	T	T	C	A	T	C	T	A	C	C	G	A	T	A	1201
G	G	C	T	G	G	C	T	T	T	C	A	T	C	T	A	C	C	G	A	T	C	1201
G	G	T	C	A	C	T	T	T	T	C	A	T	C	T	A	C	C	G	A	T	A	1201
G	G	C	T	G	G	C	T	T	T	C	A	T	C	T	A	T	C	G	A	T	A	1201
G	G	C	T	G	G	C	T	C	T	C	G	C	T	T	A	C	C	G	A	T	C	1202
G	G	C	T	G	G	C	T	C	T	C	G	C	T	T	A	C	C	G	A	T	A	1202
G	G	C	T	G	G	C	T	C	T	C	A	C	T	T	A	C	C	T	A	T	C	1301
G	G	C	T	G	G	C	T	C	T	C	A	C	T	T	A	C	C	T	A	T	C	1302
G	G	T	C	A	C	T	T	T	T	C	A	T	C	T	A	T	C	G	A	T	A	1312
G	A	T	C	A	C	T	T	T	T	C	A	C	C	T	A	C	C	G	A	T	A	1401
G	G	T	C	A	C	T	T	T	T	C	A	C	C	T	A	C	C	T	A	T	C	1401
G	G	T	C	A	C	T	T	T	T	C	A	C	C	T	A	C	C	G	A	T	A	1401
G	A	T	C	A	C	T	T	T	T	C	A	C	C	T	A	C	C	T	A	T	C	1401
G	A	C	C	G	G	C	C	T	T	C	A	T	C	T	A	T	C	G	A	T	A	1403
G	G	T	C	A	C	T	T	T	T	C	A	C	C	T	A	C	C	T	A	T	C	1404
G	G	T	C	A	C	T	T	T	T	C	A	C	C	T	A	C	C	T	A	T	A	1405
G	G	T	C	A	C	T	T	T	T	C	A	C	C	T	A	C	C	T	A	T	C	1405
G	G	T	T	G	G	C	T	T	T	C	A	C	C	T	A	C	C	T	A	T	C	1410
G	G	T	C	A	C	T	T	T	T	C	A	C	C	T	A	C	C	T	A	T	A	1418
G	G	C	C	A	C	T	T	T	T	G	A	C	T	T	G	C	C	T	A	T	C	1501
G	G	C	C	A	C	T	T	T	T	G	A	C	T	T	G	C	C	G	A	T	C	1501
G	G	C	C	A	C	T	T	T	T	G	A	C	T	T	G	C	C	G	A	T	A	1501
G	G	C	T	G	G	C	T	T	T	G	A	C	T	T	G	C	C	G	A	T	A	1502

G	G	T	T	G	G	C	T	T	T	G	A	C	T	T	G	C	C	T	G	T	C	1502
G	G	C	T	G	G	C	T	T	T	G	A	C	T	T	G	C	C	T	G	T	C	1502
G	G	T	T	G	G	C	T	T	T	G	A	C	T	T	G	C	C	T	A	T	C	1502
G	G	C	C	A	C	T	T	T	T	G	A	C	T	T	A	C	C	G	A	T	A	1602
G	G	T	C	A	C	T	T	T	T	G	A	C	T	T	A	C	C	G	A	T	A	1602

With impute, *HLA-A*, Affy5.0

A	G	C	A	G	G	G	C	C	G	T	A	T	T	T	G	A	G	C	T	G	201
G	A	C	A	G	G	G	C	C	G	T	A	T	T	T	G	A	G	C	T	G	201
A	G	C	A	G	G	G	C	C	G	T	A	T	T	T	G	A	G	C	C	G	201
A	A	C	A	G	G	G	C	C	G	T	A	T	T	T	G	A	G	C	T	G	201
A	G	C	A	G	G	G	C	C	G	T	A	T	T	T	G	A	A	C	T	G	201
A	A	C	A	G	G	G	C	C	G	T	A	T	T	T	G	A	G	C	T	G	203
A	G	C	A	G	G	G	C	C	G	T	A	T	T	T	G	A	G	C	T	G	203
A	A	C	A	G	G	G	C	C	G	T	A	C	T	T	G	A	G	C	T	G	203
G	A	C	G	G	G	G	C	C	G	C	A	T	T	T	G	A	A	C	T	A	203
G	A	C	A	G	G	G	C	C	G	T	A	T	T	T	G	A	A	C	T	A	203
A	G	C	A	G	G	G	C	C	G	T	A	C	T	T	G	A	G	C	T	G	206
A	A	C	A	G	G	G	C	C	G	T	A	C	T	T	G	A	G	C	T	G	206
G	A	C	A	G	G	G	C	C	G	T	A	C	T	T	G	A	G	C	T	G	206
A	G	C	A	G	G	G	C	C	G	T	A	T	T	T	G	A	G	C	C	G	207
A	G	C	A	G	G	A	C	C	A	T	A	T	T	T	G	A	G	C	C	G	207
A	G	C	A	G	G	G	C	C	G	T	A	T	T	T	G	A	G	C	T	G	207

G	A	T	G	G	G	G	C	C	G	C	G	T	C	G	A	A	A	A	T	G	207
G	A	C	A	G	G	G	C	C	G	T	A	T	C	G	A	A	A	A	T	G	207
G	A	T	G	G	G	A	C	T	A	T	G	T	T	T	A	A	A	C	T	G	301
G	A	C	G	G	G	G	C	C	G	C	A	T	T	T	G	A	A	C	T	A	2402
G	A	C	G	G	G	G	C	C	G	C	A	T	T	T	G	A	G	C	T	G	2402
A	G	C	G	G	G	G	C	C	G	C	A	T	T	T	G	A	A	C	T	A	2402
G	A	C	G	G	G	G	C	C	G	C	A	T	T	T	G	A	A	C	T	A	2403
G	A	C	G	G	G	G	C	C	G	C	A	T	T	T	G	A	A	C	T	A	2407
G	A	C	G	G	G	G	C	C	G	C	A	T	T	T	G	A	A	C	T	A	2410
G	A	C	A	T	T	G	T	C	G	C	A	T	T	T	G	A	A	C	T	G	2601
G	A	T	G	G	G	A	C	C	A	T	A	T	T	T	G	A	A	C	T	G	2601
G	A	C	A	T	T	G	T	C	G	C	A	T	T	T	A	A	A	A	T	G	2601
G	A	C	A	T	T	G	T	C	G	C	A	T	T	T	G	A	A	C	T	G	2602
G	A	T	G	G	G	A	C	C	A	T	A	T	T	T	G	A	A	C	T	A	2901
G	A	C	A	G	G	A	C	T	A	T	A	C	T	T	A	A	A	A	T	G	3001
G	A	T	G	G	G	G	C	C	G	C	G	T	C	G	A	G	A	C	T	G	3101
G	A	C	A	T	G	G	C	C	G	T	A	T	C	G	G	A	A	C	T	A	3201
G	A	T	G	G	G	G	C	C	G	C	G	T	C	G	A	A	A	A	T	G	3303
A	G	C	A	T	T	G	T	C	G	C	A	T	C	G	A	A	A	A	T	G	3303
A	G	C	A	G	G	A	C	C	A	T	A	T	T	T	G	A	G	A	T	G	3401
G	A	C	A	T	G	G	C	C	G	T	A	T	T	T	G	A	A	C	T	A	7401

With impute, *HLA-A*, Affy6.0

A	A	G	G	C	C	A	T	T	T	G	A	T	201
A	A	G	G	C	C	A	T	T	T	G	A	C	201
A	A	G	G	C	C	A	T	C	T	G	A	T	201
A	A	G	G	C	C	A	T	T	T	G	A	T	203
A	A	G	G	C	C	A	T	C	T	G	A	T	203
G	A	G	G	C	C	C	C	T	T	G	A	T	203
A	A	G	G	C	C	A	T	C	T	G	A	T	206
A	A	G	G	C	C	A	T	T	T	G	A	T	206
A	A	G	G	C	C	A	T	T	T	G	A	C	207
A	A	G	G	C	C	A	T	T	T	G	A	T	207
A	A	G	G	C	C	C	T	T	C	A	A	T	207
A	A	G	G	C	C	C	C	T	C	A	A	T	207
G	A	G	A	C	T	C	T	T	T	A	A	T	301
G	A	G	G	C	C	C	C	T	T	G	A	T	2402
G	A	G	G	C	C	C	C	T	T	G	A	T	2403
A	A	G	G	C	C	C	C	T	T	G	A	T	2407
G	A	G	G	C	C	C	C	T	T	G	A	T	2410
A	A	G	G	C	C	C	C	T	T	G	A	T	2410
A	G	T	G	T	C	C	C	T	T	G	A	T	2601
G	A	G	A	C	C	C	T	T	T	G	A	T	2601
A	G	T	G	T	C	C	C	T	T	G	A	T	2602
G	A	G	A	C	C	C	T	T	T	G	A	T	2901
A	A	G	A	C	T	C	T	C	T	A	A	T	3001

G	A	G	G	C	C	C	C	T	C	A	G	T	3101
A	A	G	G	C	C	C	T	T	C	G	A	T	3201
G	A	G	G	C	C	C	C	T	C	A	A	T	3303
A	G	G	G	C	C	C	C	T	C	A	A	T	3303
A	A	G	G	C	C	C	C	T	T	G	A	T	3401
A	A	G	G	C	C	C	T	T	T	G	A	T	7401

With impute, *HLA-A*, Illumina550K

C	T	G	G	C	C	G	A	T	T	A	T	A	G	T	A	C	T	C	201
T	T	G	G	C	C	G	A	T	T	A	T	A	G	T	A	C	C	C	201
C	T	G	G	C	C	G	A	T	T	A	C	A	G	T	A	C	T	C	201
T	T	G	G	C	C	G	A	T	T	A	T	A	G	T	A	C	T	C	203
C	T	G	G	C	C	G	A	T	T	A	T	A	A	T	A	C	T	T	203
C	T	G	G	C	C	G	C	C	T	A	T	A	A	T	A	C	T	T	203
C	T	G	G	C	C	G	A	T	T	A	C	A	G	T	A	C	T	C	206
T	T	G	G	C	C	G	A	T	T	A	T	A	G	T	A	C	C	C	207
C	T	G	G	C	C	G	A	T	T	A	T	A	A	T	A	C	C	C	207
C	T	G	G	C	C	G	A	T	T	A	T	A	G	T	A	C	T	C	207
C	T	T	G	T	C	G	C	C	T	A	T	T	G	C	A	T	T	T	207
T	T	G	A	C	C	G	C	C	T	G	T	T	G	C	A	T	T	T	207
C	T	G	G	C	C	G	C	C	T	G	T	T	G	C	A	T	T	T	207
C	T	G	G	C	T	A	C	T	G	G	T	A	G	T	A	C	T	C	301
C	T	G	A	C	C	G	C	C	T	A	T	A	A	T	A	C	T	T	2402

C	T	G	G	C	C	G	C	C	T	A	T	A	A	T	A	C	T	C	2402
C	T	G	G	C	C	G	C	C	T	A	T	A	G	T	A	C	T	C	2402
C	T	G	G	C	C	G	C	C	T	A	T	A	A	T	A	C	T	T	2403
C	T	G	G	C	C	G	C	C	T	A	T	A	A	T	A	C	T	T	2407
C	T	G	G	C	C	G	C	C	T	A	T	A	A	T	A	C	T	T	2410
T	T	G	G	T	C	G	C	C	T	A	T	A	G	T	A	C	T	T	2601
T	T	G	G	C	C	A	C	T	G	A	T	A	G	T	A	C	T	C	2601
C	T	G	G	T	C	G	C	C	T	A	T	A	G	T	A	C	T	T	2602
C	T	T	G	C	C	A	C	T	G	A	T	A	G	T	A	C	T	T	2901
C	T	G	G	C	T	A	C	T	G	A	C	A	G	T	A	T	T	T	3001
C	C	G	G	C	C	G	C	C	T	G	T	T	G	C	G	T	T	T	3101
C	C	G	G	C	C	G	C	T	T	A	T	T	G	C	A	C	T	T	3201
C	T	T	G	C	C	G	C	C	T	G	T	T	G	C	A	T	T	T	3303
C	T	G	G	C	C	G	C	C	T	G	T	T	G	C	A	T	T	T	3303
C	T	G	A	C	C	A	C	C	T	A	T	A	A	T	A	T	T	T	3401
T	T	G	A	C	C	G	C	T	T	A	T	T	A	T	A	C	T	T	7401

With impute, *HLA-A*, Union

C	C	T	G	G	C	C	A	T	A	T	C	A	T	C	C	201
G	C	T	G	G	C	C	A	T	A	T	C	A	T	C	C	201
C	C	T	G	G	C	C	A	T	A	T	C	A	C	C	C	201
G	C	T	G	G	C	C	A	T	A	C	C	A	T	C	C	201
G	C	T	G	G	C	C	A	T	A	T	C	A	T	C	C	203

G	C	T	G	G	C	C	A	T	A	T	T	A	T	C	C	203
C	C	T	G	G	C	C	A	T	A	T	C	A	T	C	C	203
C	C	T	G	G	C	C	A	T	A	T	C	A	T	T	C	203
G	T	T	G	G	C	C	C	C	A	T	C	A	T	T	C	203
G	C	T	G	G	C	C	A	T	A	C	C	A	T	C	C	206
C	C	T	G	G	C	C	A	T	A	C	C	A	T	C	C	206
C	C	T	G	G	C	C	A	T	A	T	C	A	C	C	C	207
C	C	T	G	G	C	C	A	T	A	T	C	A	T	C	C	207
G	C	T	G	A	C	C	C	C	G	T	C	A	T	T	C	207
C	C	C	T	G	T	C	C	C	A	T	T	A	T	T	C	207
G	C	T	G	G	C	T	C	T	G	T	T	A	T	C	C	301
G	T	T	G	G	C	C	C	C	A	T	C	A	T	T	C	2402
G	T	T	G	G	C	C	C	C	A	T	C	A	T	C	C	2402
G	T	T	G	G	C	C	C	C	A	T	C	A	T	T	C	2403
G	T	T	G	G	C	C	C	C	A	T	C	A	T	T	C	2407
G	T	T	G	G	C	C	C	C	A	T	C	A	T	T	C	2410
G	C	T	T	G	T	C	C	C	A	T	T	A	T	T	C	2601
G	T	T	G	G	C	C	C	T	A	T	T	A	T	C	C	2601
G	C	T	T	G	T	C	C	C	A	T	T	A	T	T	C	2602
G	T	T	G	G	C	C	C	T	A	T	T	A	T	T	C	2901
G	T	T	G	G	C	T	C	T	A	C	C	A	T	T	T	3001
G	C	T	G	A	C	C	C	C	G	T	C	G	T	T	C	3101
G	T	C	G	G	C	C	C	T	A	T	T	A	T	T	C	3201

G C T G A C C C C G T C A T T C 3303

G T T G G C C C C G T C A T T C 3401

G T C G G C C C T A T C A T T C 7401

With impute, *HLA-B*, Affy5.0

A C A T A A A C A C T T G T C C A C G G G G G A A G A C G

G G C 702

G C G T C G A C G T T T A T A T G C G G G G G A A G A C G

G G C 705

G C A T C G G C G C T T G T C T A C G G A A G A A G A C G

G G C 801

G C A T A A A C A C T T G T C C A C G A G G G A A G A T G

G G C 1301

G C A T C G G C G T G T G G A T G C G A G G G A A A A T G

G G C 1301

A C A C C G A T G T T T G G A T G C G A G G G A A G A T G

G G C 1301

A C A C C G A C G C T T G G C T A C G A G G G A A G A C G

G G C 1301

G C G T A G A C G T G T A T C T A C G G G G G A A G A C G

G G C 1302

G C A T C G G C G T G T G G A T G T A A A A A G A A G C G

G G C 1501

A C A T A A A C G C T T G T C T A T A A A A A G A A G C G

G G C 1501

A C A C C G A T G C T T G G A T A T A A G G G A A G A T G

G G C 1501

G C G T C G A T G T T G A G A T G T A A A A A G A A G C G

G G C 1501

A A A T C G A T G T G T A T C T A T A A A A A G A A G C G

G G C 1501

A C A C C G A T G T T T G G C T A T A A A A A G A A G C G

G G C 1501

A C A T A A A C G C T T G T C T A T A A A A G A A G A C G

G G C 1502

G C A T A A A C G C T T A T A T G T A A G A G G A A A T G

G G C 1502

G C A C C G A T G T G T A T C T A T A A A A A G A A G C G

G G C 1502

G C G T A G A C G T T G A G A T G T A A A A G A A G A C G

G G C 1502

A A A T C G A T G C T T G T C C A T A G G G G A A G A T G

G G C 1503

A C A C C G A T G T T T G G A T G T A A A A G G A G A C G

G G C 1503

G C A T A G A T G T T T G T C T A T A A A A A G A A G C G

G G C 1508

G C G T A G A C G T T T G G C T A T A A A A A G A A G C G

G G C 1511

A A A T C G A T G T G T A T C T A T A A G A G G A A A T A

G A C 1518

A C A C C G A T G T T T G G A T G T A A G A G G A A A T G

G G C 1518

A A A T C G A T G T T G A G A T G T A A G A G G A G A T G

G G C 1525

G C G T C G A C G T T T A T A T G T A A G A G G A G A T G

G G C 1525

A A A T C G G C G T G T G G A T G T A A G A G A A G A C G

G G C 1525

A A A T C G G C G T G T G G A T G T A A A A A G A A G C G

G G C 1527

G C A T C G G C G T G T A G A T G T A G G G G A A A G C G

G G C 1532

A C A T C G G C G T G T G G A T G C G A A A G A A G A C G

G G C 2704

G C A T C G G C G C T T G T C T A C G A A A G A A G A T G

G G C 2704

A A A T C G A C A C T T G G A T A C G A G G G A G G A T G

G G C 2704

A C A C C G A T G T T T A G A T G C G A A A G A A A G C G

G G C 2704

G C G T A G A C G T T T A T A T G C G A G G G A A G A T G

G G C 2705

G C A T C G G C G T G T G G A T G C G A G G G A G G A T G

G G C 2706

G C A T A A A C G C T T G T C T A C G G G G G A A G A C G

G G C 3501

G C A T A A A C A T T T A T C C A C G G G G G A A G A C G

G G C 3501

A C A C C G A T G T T T G G A T G C G G G G G A A G A C G

G G C 3501

G C G T A G A C G C T T G T C C A C G G G G G A A G A C G

G G C 3501

G C G T A G A C G T T T G T C C A C G G G G G A A G A T G

G G C 3502

G C G T A G A C G T T T G T C C A C G G G G G A A G A T G

G G C 3505

G C G T A G A C G C T T G T C C A C G A G G G A G G A T G

G A C 3701

A C A T A A A C A C T T G T C C A C G A G G G A A G A T G

G G C 3802

G C A T A A A C A C T T G T C C A C G A G G G A A G A C G

G G C 3802

G C A T A A A C A T T T A T C C A C A G G G G A A G A T G

G G C 3802

G C G T C G A T G T T G A G A T G T A A G A G G A G A T G

G G C 3901

G C A T C G G C G C T T G T C T A C G A G G G A A G A C G

G G C 3901

A C A T A A A C G C T T G T C T A T A G G G G A A G A T G

G G C 4001

A C A T A A A C A C T T G T C C A T A G G G G A A G A T G

G G C 4001

G C G T C G A C G T T T A T A T G T A G G G G A A G A T G

G G C 4001

G C A T C G G C G C T T G T C T A T A G G G G A A G A T G

G G C 4001

G C A T C G G C G T G T G G A T G T A G G G G A A G A C G

G G C 4001

G C A T C G G C G T G T G G A T G T A G G G G A A G A T G

G G C 4001

A C A C C G A T G T T T G G C T A T A G G G G A A G A C G

G G C 4001

G C A T A A A C G C T T A T A T G T A G G G G A A G A C G

G G C 4001

G C A T A A A C G C T T A T A T G T A G G G G A A G A C G

G G C 4001

A A A T C G A T G T T G A G A T G C G G G G G A A G A C G

A G A 4001

A A A T C G A T G T T G A G A T G T A A A A G G A G A T G

G G C 4001

G C A T C G G C G T G T A G A T G C G A G G G A G G A T G

G G C 4002

G C A T C G G C G T G T A T C T A C G A G G G A G G A T G

G G C 4002

G C A T C G G C G T G T A T C T A C G A G G G A G G A T G

G G C 4002

G C G T A G A T G T T T G T C T A C G G G G G A A G A T G

G G C 4002

G C A T A A A C G T G T G G A T G C G A G G G A G G A T G

G G C 4002

G C A T A A A C G T G T G G A T G C G A G G G A G G A T G

G G C 4003

A C A T A G A C G T G T A T C T A C G A G G G A G G A T G

G G C 4006

A C A T A G A C G T G T A T C T A T G G G G G A A G A C G

G G C 4403

A C A T A A A C G T G T G G A T G T G G G G G A A G A T G

G G C 4403

A C A T A A A C G C T T A T A T G T A A A A A G A A G C G

G G C 4601

G C G T A G A C G C T T G T C T A T A A A A A G A G A T G

G G C 4601

G C G T C G A C G T T T A T A T G T A A A A A G A A G C G

G G C 4601

G C G T C G A T G T T G A G A T G C G G G G G A A G A C G

G G C 4601

G C A T A G A T G T T T G T C T A T A A G A G G A G A T G

G G C 4601

A C A T A A A C G T G T A T A T G C G G G G G A A G A C G

A G A 4601

G C A T C G G C G C T T G T C T G C G G G G G A A G A T G

G G C 4801

G C A T C G G C G T G T G G A T A C A A A A A G A A G C G

G G C 4801

G C G T C G A T G T T G A G A T G C G G A A G A A G A C G

G G C 4801

G C A T A G A T G T T T G T C T A C G G G G G A A G A C G

G G C 4801

A C A C C G A T G T T G A G A T G T A A G G G A A G A T G

G G C 5101

G C G T C G A T G T T T G G A T G T A A G G G A A G A T G

G G C 5101

A C A C C G A T G C T T G T C T A C A G G G G A A G A T G

G G C 5101

A C A T A A A C A C T T G T C T A T A A G G G A A G A T G

G G C 5101

G C G T A G A C G T T T G G C T A T A A G G G A A G A T G

G G C 5101

G C G T A G A C G T T T G T C T A C G A G G G A A G A C G

G G C 5101

A C A C C G A T G C T T G T C T A C G A G G G A A G A C G

G G C 5101

G C A T A A A C G T T G A G A T G T A A G G G A A G A T G

G G C 5102

G C G T C G A T G T T T G T C T A T A A G G G A A G A T G

G G C 5102

A C A C C G A T G T G T A T A T G T A A G G G A A G A T G

G G C 5201

G C A T C G A T G T T T G G A T G C A G G G G A A G A T G

G G C 5401

A A A T C G A C G T T G A G A T G C A G G G G A A G A T G

G G C 5401

G C A T C G A C G C T T G G C T A C A G G G G A A G A T G

G G C 5502

A C G T C G A T G C T T G G C C A C A G G G G A A G A T G

G G C 5502

G C G T A G A C G C T T G G C T A C A G G G G A A G A T G

G G C 5502

G C A T C G G C G C T T G T C T A C A G G G G A A G A T G

G G C 5502

A C A C C G A C G C T T G G C T A C A G G G G A A G A T G

G G C 5502

G C G T A G A T G T T T G T C T A C A G G G G A A G A T G

G G C 5502

A C A C C G A T G T T T A G A T G T A A A A A G A A G C G

G G C 5504

G C A T A A A C G T T G A G A T G C A G G G G A A G A T G

G G C 5601

A C A T A A A C G T G T A T A T G C A G G G G A A G A T G

G G C 5601

G C G T A G A T G T T T G T C T A C A G G G G A A G A C G

G G C 5601

G C A T C G G C G T G T A T C T G T A A G A G G A G A T G

G G C 5601

G C G T A G A T G T T T G T C T A C G G G G G A A G A C G

A G A 5601

A	C	A	C	C	G	A	T	G	C	T	T	G	T	C	C	A	C	A	G	G	G	G	A	A	G	A	T	G
	G	G	C	5603																								
G	C	G	T	C	G	A	T	G	T	T	G	A	G	A	T	G	C	A	G	G	G	G	A	A	G	A	T	G
	G	G	C	5604																								
G	C	A	T	C	G	G	C	G	T	G	T	A	T	C	T	G	T	G	G	G	G	G	A	A	G	A	T	G
	G	G	C	5701																								
G	C	G	T	A	G	A	C	G	T	T	T	G	G	C	T	A	C	G	G	G	G	G	A	A	G	A	C	G
	A	G	A	5801																								
G	C	G	T	A	G	A	T	G	T	T	T	G	T	C	T	A	C	G	G	G	G	G	A	A	G	A	T	G
	G	G	C	5801																								
G	C	G	T	A	G	A	T	G	T	T	T	G	T	C	T	A	C	G	G	G	G	G	A	A	G	A	C	G
	G	G	C	5801																								
A	A	A	T	C	G	A	C	A	C	T	T	G	G	A	T	A	C	A	G	G	G	G	A	A	G	A	T	G
	G	G	C	5901																								
A	A	A	T	C	G	A	C	A	C	T	T	G	G	A	T	A	C	G	A	G	G	G	A	A	G	A	C	G
	G	G	C	6701																								

With impute, *HLA-B*, Affy6.0

G	A	A	T	C	G	C	G	A	T	G	G	A	G	T	C	A	C	G	T	G	702
G	G	G	T	A	A	C	G	A	T	A	T	A	A	G	T	G	T	A	T	G	705
G	G	G	T	C	G	T	G	A	T	G	G	A	G	G	C	A	C	G	T	G	801
G	A	G	T	A	A	C	G	A	T	G	G	A	A	G	T	A	C	G	C	G	1301
G	G	G	T	C	G	C	G	A	T	G	G	A	A	G	T	A	C	G	T	G	1301

G	G	A	T	A	G	C	G	A	T	G	G	A	A	G	T	A	C	G	C	G	1301
G	A	G	C	C	G	C	A	A	T	G	G	A	A	G	T	A	C	G	C	G	1301
G	G	G	C	C	G	T	G	A	T	G	G	A	A	T	C	A	C	G	T	G	1302
G	A	A	T	C	G	T	A	A	T	G	G	A	A	T	C	A	C	G	T	G	1302
G	G	G	T	C	G	C	G	A	T	G	T	G	A	G	T	A	T	G	T	G	1501
G	A	G	T	A	A	T	G	A	T	G	T	G	A	G	T	A	T	G	T	G	1501
G	G	G	T	C	G	C	G	A	T	G	T	G	A	G	T	A	T	G	T	G	1501
G	G	G	C	C	G	C	G	A	T	G	T	G	A	G	T	A	T	G	T	G	1501
G	G	G	C	C	G	C	G	A	T	G	T	G	A	G	T	A	C	A	C	G	1501
G	A	G	T	A	A	T	G	A	T	G	T	G	A	G	T	A	T	G	T	G	1502
G	G	G	T	C	G	T	G	A	T	G	T	G	A	G	T	A	T	G	C	G	1502
T	A	A	T	A	G	T	G	A	T	G	T	G	A	G	T	A	T	G	T	G	1502
G	G	A	T	A	G	T	G	A	T	G	T	G	A	G	T	A	T	G	T	G	1503
G	A	G	T	A	A	C	A	A	T	G	T	A	A	G	C	A	C	G	C	G	1503
G	G	G	C	C	G	C	G	A	T	G	T	G	A	G	T	A	T	G	T	G	1508
G	A	G	T	A	G	C	G	A	T	G	T	G	A	G	T	A	T	G	T	G	1511
T	A	A	T	A	G	T	G	A	T	G	T	G	A	G	T	A	T	G	C	A	1518
G	A	G	T	C	G	T	G	A	T	G	T	G	A	G	T	A	T	G	C	G	1518
G	A	G	C	C	G	T	G	A	T	G	T	G	A	G	T	A	T	G	T	G	1525
G	A	G	T	A	A	T	G	A	T	G	G	A	A	G	T	A	T	G	C	G	1525
G	G	G	T	C	G	C	G	A	T	G	T	G	A	G	T	A	T	G	T	G	1527
G	G	G	T	C	G	T	G	A	T	G	T	G	A	G	T	A	T	G	T	G	1532
G	G	G	T	C	G	T	G	A	T	G	T	A	A	G	C	A	C	G	C	G	1532

G	G	G	C	C	G	T	G	A	G	A	G	A	A	G	T	G	C	G	T	G	2704
G	G	G	T	C	G	T	G	A	G	A	G	A	A	G	T	G	C	G	C	G	2704
G	A	G	T	A	A	C	G	C	T	G	G	G	A	G	T	G	C	A	C	G	2704
G	G	G	T	A	G	T	G	A	G	A	G	A	A	G	T	G	C	A	C	G	2705
G	G	A	C	C	G	C	G	A	G	A	G	A	A	G	T	G	C	G	C	G	2706
G	G	G	C	C	G	C	G	A	T	A	T	A	A	G	T	G	T	A	T	G	3501
G	A	G	T	A	G	C	A	A	T	A	T	A	A	G	T	G	T	A	T	G	3501
G	G	A	T	A	G	C	G	A	T	A	T	A	A	G	T	G	T	A	T	G	3501
G	G	A	T	A	G	C	G	C	T	A	T	A	A	G	T	G	T	A	T	G	3501
G	G	G	C	C	G	C	G	A	T	G	T	G	A	G	T	G	T	A	T	G	3501
G	G	G	C	C	G	T	G	A	T	A	T	A	A	G	T	A	T	A	C	G	3501
G	G	G	T	C	G	T	G	C	T	A	T	A	A	G	T	G	T	A	C	G	3502
G	G	G	T	C	G	C	G	C	T	A	T	A	A	G	T	G	T	A	T	G	3505
G	G	G	C	C	G	T	G	A	T	A	G	A	A	G	T	G	C	G	C	G	3701
G	A	G	C	C	G	T	G	A	T	G	G	A	A	G	T	G	C	A	C	G	3802
G	G	G	T	C	G	T	G	A	T	G	G	A	A	G	T	G	C	A	T	G	3802
G	G	G	T	C	G	T	G	A	G	A	G	A	A	G	T	G	C	G	C	G	3802
G	A	G	C	C	G	C	G	C	T	G	G	G	A	G	T	G	C	A	C	G	3802
G	A	G	T	A	A	T	G	A	T	G	G	A	A	G	T	A	T	G	C	G	3901
G	G	G	T	C	G	T	G	A	T	G	G	A	A	G	T	G	C	A	T	G	3901
T	A	A	T	A	G	C	A	A	T	G	T	A	A	G	C	A	C	G	C	G	4001
G	A	G	T	C	G	C	A	A	T	G	T	A	A	G	C	A	C	G	C	G	4001
G	G	G	T	C	G	T	G	A	T	G	T	A	A	G	C	A	C	G	C	G	4001

G	G	G	T	C	G	C	G	A	T	G	T	A	A	G	C	A	C	G	C	G	4001
G	G	G	T	C	G	C	G	A	T	G	T	A	A	G	C	A	C	G	T	G	4001
G	A	A	T	C	G	C	G	A	T	A	T	A	A	G	T	G	T	A	T	G	4001
G	G	G	C	C	G	T	G	A	T	G	T	G	A	G	T	A	T	G	T	G	4001
G	A	G	T	C	G	T	G	A	T	G	T	A	A	G	C	A	C	G	C	G	4001
G	G	G	T	A	G	T	G	A	G	A	G	A	A	G	T	G	C	G	C	G	4002
G	G	G	T	A	A	T	G	A	G	A	G	A	A	G	T	G	C	G	C	G	4002
G	G	G	T	A	A	T	G	A	G	A	G	A	A	G	T	G	C	G	C	G	4002
G	A	A	T	C	G	C	G	A	G	A	G	A	A	G	T	G	C	A	C	G	4002
G	A	A	T	C	G	T	G	A	G	A	G	A	A	G	T	G	C	G	C	G	4003
G	A	G	T	C	G	C	G	A	G	A	G	A	A	G	T	G	C	G	C	G	4006
G	G	G	C	C	G	C	A	A	T	A	T	G	A	G	T	A	T	G	T	G	4403
G	G	G	C	C	G	C	G	A	T	G	T	G	A	G	T	A	T	G	T	G	4601
T	A	A	T	A	G	T	G	A	T	G	T	G	A	G	T	A	T	G	T	G	4601
G	G	G	T	C	G	C	G	A	T	G	T	G	A	G	T	A	T	G	C	G	4601
G	G	G	T	C	G	C	G	A	T	G	T	G	A	G	T	A	T	G	T	G	4601
G	A	G	T	A	A	C	G	A	T	A	T	A	A	G	T	G	T	A	T	G	4601
G	A	G	T	A	A	C	G	A	T	G	G	A	G	G	T	A	C	G	C	G	4801
G	G	G	T	C	G	C	G	A	T	G	G	A	G	G	T	A	C	G	T	G	4801
G	G	G	T	C	G	T	G	A	T	G	G	A	G	G	T	A	T	G	T	G	4801
G	A	G	T	A	A	C	G	C	T	A	T	A	A	G	T	A	C	A	C	G	5101
T	A	A	T	A	G	C	G	C	T	A	T	A	A	G	T	G	C	A	T	G	5101
G	G	G	T	A	A	T	G	C	T	A	G	G	A	G	T	G	C	A	C	G	5101

T	A	A	T	A	G	T	G	C	T	A	T	A	A	G	T	A	C	A	C	G	5101
T	A	G	T	A	G	C	G	C	T	A	T	A	A	G	T	A	C	A	C	G	5101
G	A	G	T	A	A	T	G	C	T	A	T	A	A	G	T	A	C	A	C	G	5102
G	G	G	C	C	G	T	G	C	T	A	T	A	A	G	T	A	T	G	C	G	5201
G	A	G	C	C	G	T	G	C	T	A	T	A	A	G	T	A	C	A	C	G	5201
G	A	G	T	A	A	C	G	C	T	G	G	G	A	G	T	G	C	A	C	G	5401
G	A	G	C	C	G	C	G	C	T	G	G	G	A	G	T	G	C	A	T	G	5401
G	G	G	T	A	A	C	G	C	T	G	G	G	A	G	T	G	C	A	C	G	5401
T	A	A	T	A	G	C	G	C	T	G	G	G	A	G	T	G	C	A	C	G	5502
G	G	G	T	A	A	T	G	C	T	G	G	G	A	G	T	G	C	A	C	G	5502
G	A	G	T	A	A	C	G	C	T	G	G	G	A	G	T	G	C	A	C	G	5502
G	G	G	T	C	G	C	G	C	T	G	T	G	A	G	T	G	C	A	C	G	5502
G	G	G	T	C	G	C	G	C	T	G	G	G	A	G	T	G	C	A	C	G	5502
G	A	G	T	A	A	C	G	C	T	G	G	G	A	G	T	G	C	A	C	G	5502
G	A	A	T	C	G	T	G	A	T	G	G	G	A	G	T	G	C	A	C	G	5502
G	A	G	T	C	G	C	G	C	T	G	G	G	A	G	T	A	T	G	T	G	5504
G	A	G	T	C	G	C	G	C	T	G	G	G	A	G	T	G	C	A	T	G	5601
G	G	G	T	A	G	C	G	C	T	G	G	G	A	G	T	G	C	A	C	G	5601
G	G	G	T	A	G	T	A	A	T	G	G	A	G	T	C	A	C	G	T	G	5601
G	A	G	T	C	G	C	G	C	T	G	G	G	A	G	T	G	C	A	C	G	5603
G	A	G	T	C	G	C	G	C	T	G	G	G	A	G	T	G	C	A	C	G	5604
G	A	G	T	A	A	C	G	A	T	G	T	G	A	G	T	G	T	A	T	G	5701
G	A	G	T	A	A	T	G	A	T	A	T	A	A	G	T	A	T	A	C	G	5701

G	G	A	C	C	G	C	G	A	T	A	T	A	A	G	T	G	T	A	T	G	5801
G	G	A	C	C	G	C	G	C	T	G	G	G	A	G	T	G	C	A	C	G	5901
G	G	A	C	C	G	T	G	A	T	G	G	A	A	G	T	G	C	A	T	G	6701
G	G	A	C	C	G	T	G	A	T	G	G	A	A	G	T	G	C	A	T	G	6701

With impute, *HLA-B*, Illumina550K

T	G	C	A	C	G	A	C	T	C	C	G	T	A	A	T	102
T	G	C	A	C	G	A	C	T	C	C	G	G	A	A	C	102
G	G	C	A	C	G	A	C	T	C	C	G	T	A	A	C	102
G	A	C	G	T	G	A	T	T	C	C	G	T	A	A	T	102
T	G	C	A	C	G	A	C	T	C	C	G	G	A	G	T	102
T	A	C	A	T	G	A	C	T	C	T	G	T	A	A	C	102
T	A	C	G	C	G	A	T	T	A	C	A	G	T	A	T	102
T	G	C	A	C	G	A	T	T	A	T	G	T	A	A	T	102
T	G	C	A	C	G	A	C	T	C	C	G	T	A	A	T	103
T	A	C	A	T	T	C	C	T	C	T	G	G	A	G	C	202
T	A	C	G	T	G	A	T	T	C	C	G	T	A	A	T	302
G	A	C	G	T	G	A	T	T	A	C	G	T	A	A	T	303
T	G	C	A	C	G	A	C	T	C	C	G	G	A	A	C	303
T	G	C	A	C	G	A	C	T	C	C	G	T	A	A	T	303
T	A	C	G	T	G	A	T	T	A	C	G	G	A	G	C	303
T	A	C	G	T	G	A	T	T	A	C	A	T	A	A	C	304
T	A	C	G	T	T	C	C	C	C	C	G	G	A	A	C	304

T	A	C	A	T	G	A	C	T	C	T	G	G	A	G	C	304
T	A	C	G	T	G	A	T	T	A	C	G	T	A	A	C	304
G	A	C	A	T	G	A	T	T	A	C	G	T	A	A	T	401
T	A	C	G	T	G	A	T	T	A	C	A	T	A	A	C	401
T	A	C	A	T	G	A	T	T	A	C	A	T	A	A	C	401
T	A	C	A	T	G	A	T	T	A	C	G	G	A	A	T	401
G	A	C	A	T	G	A	T	T	A	C	G	T	A	A	T	401
T	A	C	A	T	G	A	T	T	A	T	G	T	A	A	C	403
T	A	C	A	T	G	A	T	T	A	T	G	T	A	A	T	403
T	A	C	G	T	T	A	C	C	C	T	G	G	A	A	C	403
T	A	T	G	T	T	A	C	C	C	T	G	G	T	A	C	602
T	A	T	G	T	T	A	C	C	C	T	G	G	A	G	C	602
G	A	T	G	T	T	A	C	C	C	T	G	T	A	A	T	602
T	A	T	G	T	G	A	C	T	C	C	A	G	T	A	C	602
T	A	C	A	T	G	A	T	T	A	C	A	T	A	A	T	701
T	A	C	G	T	T	A	T	T	A	C	A	T	A	A	C	702
T	A	C	G	T	T	A	C	C	C	T	G	G	A	A	C	702
T	A	C	G	T	T	A	C	C	C	T	G	G	A	A	T	702
T	G	C	A	C	G	A	C	T	C	C	G	G	A	A	C	702
T	A	C	G	T	T	A	T	T	A	C	G	G	A	A	C	702
T	A	C	G	T	T	A	C	C	C	T	G	G	A	G	C	702
T	A	C	G	T	G	A	T	T	A	C	G	T	A	A	T	702
T	A	C	G	T	T	A	C	C	C	T	G	T	A	A	C	702

T	A	C	G	T	T	A	C	C	C	T	G	T	A	A	T	702
T	A	C	G	T	T	A	C	C	C	T	G	G	T	A	C	702
G	A	C	A	T	G	A	T	T	A	C	A	T	A	A	T	702
T	A	C	G	T	G	A	T	T	C	T	G	G	A	A	C	702
G	A	C	A	T	G	A	C	T	C	C	G	G	T	A	T	702
T	G	C	A	C	G	A	T	T	A	T	G	T	A	A	T	704
T	A	C	A	T	G	A	T	T	A	T	G	T	A	A	T	801
G	A	C	G	C	G	A	T	T	A	C	G	G	A	G	C	801
T	A	C	G	C	G	A	T	T	A	C	G	G	T	A	C	801
T	A	C	G	C	G	A	T	T	A	T	G	T	A	A	T	801
T	A	C	G	C	G	A	T	T	A	C	G	T	A	A	C	801
T	A	C	G	C	G	A	T	T	A	C	G	T	A	A	T	801
T	A	C	G	T	T	A	T	T	A	C	A	T	A	A	C	801
T	G	C	G	C	G	A	T	T	C	T	G	G	A	G	C	1202
G	A	C	A	T	T	C	C	C	C	T	G	T	A	A	T	1202
G	A	C	A	T	T	C	C	C	C	T	G	T	A	A	C	1202
G	A	C	A	T	G	A	C	T	C	T	G	G	A	G	C	1202
T	G	C	G	C	G	A	T	T	C	T	G	T	A	A	T	1202
G	A	T	G	T	T	A	C	C	C	C	G	G	A	A	C	1203
T	A	T	G	T	T	A	C	C	C	T	G	T	A	A	T	1203
T	G	C	G	C	G	A	T	T	A	C	G	T	A	A	C	1402
T	G	C	A	C	G	A	C	T	C	C	G	T	A	A	T	1402
T	G	C	G	C	G	A	T	T	A	C	G	G	T	A	T	1402

T	A	C	A	T	G	A	C	T	C	T	G	T	A	A	C	1502
T	A	C	A	T	G	A	C	T	C	T	G	G	A	A	C	1502
T	G	C	A	C	G	A	C	T	C	C	G	G	A	A	C	1502
G	A	C	G	T	G	A	T	T	A	C	G	T	A	A	T	1505

With impute, *HLA-B*, Union

A	C	T	A	G	G	G	G	C	A	A	T	T	C	A	G	G	A	C	A	T	C	G	G	T	G	702
G	T	C	A	G	G	G	G	A	A	G	T	T	C	A	G	A	G	A	A	T	C	G	G	C	G	705
A	C	T	A	G	G	A	G	C	A	G	T	T	T	A	G	A	G	C	G	C	C	A	G	T	A	801
A	C	T	A	A	G	G	A	C	A	G	T	T	C	A	G	G	G	C	G	C	C	G	G	T	G	1301
A	C	T	A	G	G	G	G	A	A	G	T	T	C	A	G	G	G	C	G	C	C	G	G	T	G	1301
G	C	T	G	G	A	A	G	C	G	G	T	T	C	A	G	G	G	C	G	C	C	G	G	T	G	1301
A	C	T	A	G	G	G	G	C	A	G	T	T	C	A	G	G	A	C	A	T	C	G	G	T	G	1302
G	T	C	A	G	A	G	G	A	A	G	T	T	C	G	A	A	G	C	A	C	C	A	A	T	A	1501
A	C	T	A	G	G	G	G	C	A	G	T	T	C	G	A	A	G	C	A	C	C	A	A	T	A	1501
G	C	T	G	G	A	A	G	C	G	G	T	T	C	G	A	A	G	C	A	C	C	A	A	T	A	1501
G	C	T	G	G	G	A	G	C	A	G	T	T	C	G	A	A	G	C	A	C	C	A	A	T	A	1501
G	C	T	G	G	A	A	G	C	G	G	T	T	C	G	A	A	G	C	A	C	C	A	A	T	G	1501
G	C	T	A	G	A	A	G	A	A	G	T	T	C	G	A	A	G	C	A	C	C	G	A	T	A	1502
G	C	T	G	G	A	A	G	A	A	G	T	T	C	G	A	A	G	C	A	C	C	G	A	T	A	1502
G	C	T	A	G	G	A	G	A	A	G	C	T	C	G	A	A	G	C	A	C	C	A	A	T	G	1503
A	C	T	A	G	G	G	G	A	A	A	T	T	C	A	G	A	G	C	G	C	C	A	A	T	G	1503
G	C	T	G	G	A	A	G	C	G	G	T	T	C	G	A	A	G	C	A	C	C	A	A	T	A	1508

G	T	C	A	G	G	G	G	A	A	G	T	T	C	G	A	A	G	C	A	C	C	A	A	T	A	1511
G	C	T	G	G	A	A	G	A	A	G	C	T	C	G	A	A	G	C	A	C	C	A	A	T	G	1518
G	C	T	A	G	G	A	G	A	A	G	C	T	C	G	A	A	G	C	A	C	C	A	A	T	G	1518
A	C	T	A	G	A	A	G	A	A	G	T	T	C	G	A	A	G	C	A	C	C	G	A	T	A	1525
A	C	T	A	G	G	G	G	C	A	G	C	T	C	G	A	A	G	C	A	C	C	G	A	T	A	1525
G	T	C	A	G	A	G	G	A	A	G	T	T	C	G	A	A	G	C	A	C	C	A	A	T	A	1527
A	C	T	A	G	G	G	G	C	A	G	T	T	C	G	A	A	G	C	A	C	C	A	A	T	G	1532
G	C	T	G	G	G	A	G	C	A	G	C	G	C	A	G	A	G	A	A	T	T	G	G	T	A	2704
G	T	C	A	G	A	G	G	C	A	G	C	G	C	A	G	A	G	A	A	T	T	G	G	T	G	2704
G	C	C	A	G	A	G	G	C	A	G	C	G	C	A	G	A	G	A	A	T	T	G	G	T	G	2705
G	T	C	A	G	G	G	G	A	A	A	T	G	C	A	G	A	G	A	A	T	T	G	G	T	G	2706
G	T	C	A	G	G	G	G	A	A	G	T	T	C	A	G	A	G	A	A	T	C	G	G	C	G	3501
G	T	C	A	G	A	G	G	A	A	G	C	T	C	A	G	A	G	A	A	T	C	G	G	C	G	3501
A	C	T	A	G	G	G	G	C	A	G	C	T	C	A	G	A	G	A	A	T	C	G	G	C	G	3502
G	C	C	A	G	A	G	G	A	A	G	C	T	C	A	G	A	G	A	A	T	C	G	G	C	G	3505
A	C	T	A	G	G	G	G	C	A	G	C	T	C	A	G	A	G	A	A	T	T	G	G	T	G	3701
A	C	T	A	G	G	G	G	C	A	G	C	T	T	A	G	A	G	A	A	T	C	G	G	C	G	3802
G	C	T	G	G	A	A	G	C	G	G	C	T	C	G	G	A	G	A	A	C	C	A	A	T	G	3802
A	C	T	A	G	G	G	G	C	A	G	C	T	T	A	A	A	G	C	A	C	C	A	A	T	G	3901
A	C	T	A	G	G	G	G	C	A	G	C	T	T	A	G	A	G	A	A	T	C	G	G	C	G	3901
A	C	T	A	G	G	G	G	A	A	A	T	T	C	A	G	A	G	C	G	C	C	A	A	T	G	4001
G	T	C	A	G	G	G	G	A	A	A	T	T	C	A	G	A	G	C	G	C	C	A	A	T	G	4001
A	C	T	A	G	A	A	G	A	A	G	T	T	C	A	G	A	G	C	G	C	C	A	A	T	G	4001

G	T	C	A	G	A	G	G	C	A	G	T	T	C	A	G	A	G	C	G	C	C	A	A	T	G	4001
G	T	C	A	G	A	G	G	A	A	A	T	T	C	A	G	A	G	C	G	C	C	A	A	T	G	4001
G	T	C	A	G	G	G	G	A	A	G	T	T	C	A	G	A	G	A	A	T	C	G	G	C	G	4001
G	T	C	A	G	G	G	G	A	A	G	T	T	C	A	G	A	G	C	G	C	C	A	A	T	G	4001
G	C	T	A	G	A	A	G	A	A	G	C	T	C	A	G	A	G	C	G	C	C	A	A	T	G	4001
G	T	C	A	G	A	G	G	C	A	G	C	G	C	A	G	A	G	A	A	T	T	G	G	T	G	4002
A	C	T	A	G	G	G	G	C	A	G	C	G	C	A	G	A	G	A	A	T	T	G	G	T	G	4002
G	C	T	G	G	G	A	G	C	A	G	C	G	C	A	G	A	G	A	A	T	T	G	G	T	G	4002
G	T	C	A	G	G	G	G	A	A	G	C	G	C	A	G	A	G	A	A	T	T	G	G	T	G	4002
G	T	C	A	G	A	G	G	C	A	G	C	G	C	A	G	A	G	A	A	T	T	G	G	T	G	4003
G	C	T	A	G	G	A	G	A	A	G	C	G	C	A	G	A	G	A	A	T	T	G	G	T	G	4006
G	C	C	A	G	A	G	G	A	A	A	C	T	C	G	G	G	G	C	G	C	C	A	G	T	G	4403
G	T	C	A	G	A	G	G	A	A	G	C	T	C	G	G	G	G	C	G	C	C	A	G	T	G	4403
G	C	T	G	G	A	A	G	C	G	G	T	T	C	G	A	A	G	C	A	C	C	A	A	T	A	4601
G	T	C	A	G	A	G	G	A	A	G	T	T	C	G	A	A	G	C	A	C	C	A	A	T	A	4601
G	T	C	A	G	G	A	G	C	A	G	C	T	C	A	G	A	G	A	A	T	C	G	G	C	G	4601
G	T	C	A	G	A	G	G	C	A	G	C	T	C	A	A	A	G	C	A	C	C	A	A	T	G	4601
G	C	T	A	G	G	A	G	A	A	G	C	T	C	A	A	A	G	C	A	C	C	G	G	T	G	4801
G	T	C	A	G	A	G	G	C	A	G	C	T	C	A	A	A	G	C	A	C	C	A	A	T	A	4801
G	C	T	A	G	G	A	G	A	A	G	T	T	C	A	A	A	G	C	A	C	C	G	G	T	A	4801
G	C	T	G	G	G	A	G	A	A	G	C	T	C	A	A	A	G	C	A	C	C	A	A	T	G	5101
G	C	T	G	G	G	A	G	A	A	G	C	T	C	A	G	A	G	A	A	T	C	G	G	C	G	5101
A	C	T	A	G	G	G	G	C	A	G	C	T	C	A	A	A	G	C	A	C	C	A	A	T	G	5101

G	T	C	A	G	A	G	G	C	A	G	C	T	C	G	G	A	G	A	A	C	C	A	A	T	G	5101
G	C	T	G	G	A	A	G	C	G	G	C	T	C	A	A	A	G	C	A	C	C	A	A	T	G	5101
G	T	C	A	G	A	G	G	C	A	G	C	T	C	A	G	A	G	A	A	C	C	A	A	T	G	5101
G	T	C	A	G	A	G	G	C	A	G	C	T	C	A	A	A	G	C	A	C	C	A	A	T	G	5102
A	C	T	A	G	G	G	G	C	A	G	C	T	C	A	A	A	G	C	A	C	C	A	A	T	G	5102
G	T	C	A	G	A	G	A	C	A	G	C	T	C	A	A	A	G	C	A	C	C	A	A	T	G	5201
G	C	T	G	G	A	A	G	C	G	G	C	T	C	G	G	A	G	A	A	C	C	A	A	T	G	5401
A	C	T	A	G	G	G	G	A	A	G	C	T	C	G	G	A	G	A	A	C	C	A	A	T	G	5401
G	C	T	G	G	A	A	G	C	G	G	C	T	C	G	G	A	G	A	A	C	C	A	A	T	G	5502
G	T	C	A	G	A	G	G	C	A	G	C	T	C	G	G	A	G	A	A	C	C	A	A	T	G	5502
A	C	T	A	G	G	G	G	C	A	G	C	T	C	G	G	A	G	A	A	C	C	A	A	C	G	5502
G	T	C	A	G	G	G	G	A	A	A	T	T	C	G	G	A	G	A	A	C	C	A	A	T	G	5502
G	C	T	A	G	G	A	G	A	A	G	C	T	C	G	G	A	G	A	A	C	C	A	A	T	G	5502
G	C	T	G	G	A	A	G	C	G	G	C	T	C	G	G	A	G	C	A	C	C	A	A	T	A	5504
G	C	T	G	G	A	A	G	C	G	G	C	T	C	G	G	A	G	A	A	C	C	A	A	T	G	5601
G	T	C	A	G	G	G	G	A	A	G	T	T	C	G	G	A	G	A	A	C	C	A	A	T	G	5601
G	C	T	A	G	G	A	G	A	A	A	T	T	C	G	G	G	A	C	A	T	C	G	G	T	G	5601
G	C	T	G	G	A	A	G	C	G	G	T	T	C	G	G	A	G	A	A	C	C	A	A	T	G	5603
G	C	T	G	G	A	A	G	C	G	G	C	T	C	G	G	A	G	A	A	C	C	A	A	T	G	5604
A	C	T	A	G	G	G	G	C	A	G	T	T	C	G	A	A	G	C	A	C	C	A	G	C	G	5701
G	T	C	A	G	G	A	G	C	A	G	C	T	C	A	G	A	G	A	A	T	C	G	G	C	G	5801
G	C	T	G	G	A	A	G	C	G	G	C	T	C	G	G	A	G	A	A	C	C	A	A	T	G	5901
A	C	T	A	G	G	G	G	C	A	G	C	T	T	A	G	A	G	A	A	T	C	G	G	C	G	6701

A	T	T	A	G	G	G	G	C	A	G	C	T	T	A	G	A	G	A	A	T	C	G	G	C	G	6701

With impute, *HLA-C*, Affy5.0

T	C	C	G	A	C	G	C	A	G	T	G	C	A	T	A	C	A	A	G	C	102
T	T	T	G	A	A	G	T	A	G	T	G	C	A	T	A	C	A	A	G	C	102
G	C	C	G	A	C	G	C	A	G	T	G	C	A	T	A	C	A	A	G	C	102
G	C	T	G	A	A	G	T	A	A	T	T	C	A	T	A	T	A	A	G	T	102
G	C	T	G	A	C	G	C	A	G	T	T	T	A	T	A	C	A	C	G	T	102
T	C	T	G	A	A	G	T	G	G	T	T	T	A	T	G	C	A	C	G	T	102
T	C	C	G	A	C	G	C	A	G	T	G	C	A	T	A	C	A	A	G	C	103
G	C	T	G	A	A	G	T	G	G	T	T	C	G	T	A	C	A	A	G	C	202
G	C	T	G	G	A	G	T	G	G	T	T	T	A	T	G	C	A	C	G	T	302
G	T	T	G	A	A	G	T	G	G	T	T	T	A	T	G	C	A	C	G	T	303
T	T	T	G	A	A	G	T	A	G	T	G	C	A	T	A	C	A	A	G	C	303
T	C	T	G	A	A	G	T	G	G	T	T	T	A	T	G	C	A	C	G	T	303
T	C	T	G	A	A	G	T	A	G	T	T	C	A	T	A	C	A	C	G	T	303
G	C	T	G	A	A	G	C	A	G	T	T	C	A	T	A	C	A	C	G	T	303
T	T	T	G	A	A	G	T	A	G	T	T	T	A	T	G	C	A	A	A	T	304
G	T	T	G	A	A	G	T	G	G	T	T	T	A	T	G	C	A	C	G	T	304
T	T	T	G	A	A	G	T	A	G	T	T	T	A	T	G	C	A	C	G	T	304
T	T	T	G	A	A	G	T	G	G	T	T	T	A	T	G	C	A	C	G	T	304
G	C	T	G	A	A	G	T	A	G	T	T	C	G	T	A	C	A	C	G	T	401
G	C	T	G	A	A	G	T	G	G	T	T	C	G	T	A	C	A	C	G	T	401

G	C	T	G	A	A	G	T	A	G	T	T	C	A	C	G	C	A	C	G	T	401
G	C	T	G	A	A	G	T	A	G	T	T	C	A	C	G	C	A	A	G	T	403
G	C	T	A	A	A	A	T	A	G	T	T	C	A	C	A	C	A	A	G	T	403
G	C	T	A	A	A	G	T	A	G	T	T	C	A	C	A	C	A	A	G	T	403
G	C	T	A	A	A	G	C	A	G	C	T	T	A	C	A	C	G	A	A	T	403
G	C	C	G	A	A	A	T	G	G	T	T	C	A	C	G	C	A	A	A	T	602
G	C	T	G	G	A	G	C	A	G	C	T	T	A	T	A	T	A	A	G	C	701
G	C	T	G	A	A	G	C	G	G	C	T	T	A	C	A	C	G	A	A	T	702
G	C	T	G	A	A	G	C	A	G	C	T	T	A	C	A	C	G	A	A	T	702
G	C	T	G	A	A	G	C	G	G	C	T	T	A	C	A	C	G	C	G	T	702
T	T	T	G	A	A	G	T	A	G	T	T	T	A	T	G	C	A	C	G	T	702
G	C	T	G	G	A	G	C	A	G	C	T	T	A	T	A	T	A	A	G	C	702
G	C	T	G	A	A	G	T	A	G	T	G	C	A	T	A	C	A	A	G	C	702
G	C	T	G	G	A	G	T	G	G	T	T	T	A	T	G	C	A	C	G	T	702
G	C	T	G	A	A	G	C	G	G	C	T	T	A	C	G	C	A	C	G	T	704
G	C	T	G	A	C	G	C	A	G	T	T	T	A	T	A	C	A	C	G	T	704
G	C	T	G	A	A	G	T	A	A	T	T	C	A	T	A	T	A	A	G	T	801
G	C	T	G	A	A	G	C	G	G	C	T	T	A	C	A	C	G	A	A	T	801
G	C	T	G	A	A	G	T	A	G	T	T	C	A	C	G	C	A	A	G	C	1202
G	C	T	G	A	A	G	T	A	G	T	T	C	A	C	G	C	A	C	G	T	1202
T	T	T	G	A	A	G	T	A	G	T	G	C	A	T	A	C	A	A	G	C	1202
G	C	T	A	A	A	A	T	A	G	T	T	C	A	C	A	C	A	A	A	T	1203
T	C	C	G	A	A	A	T	G	G	T	T	C	A	C	G	C	A	A	A	T	1203

G	C	T	G	A	A	G	T	A	G	T	T	C	A	T	A	C	A	C	G	T	1402
G	C	T	G	A	A	G	T	A	G	T	T	C	A	T	A	C	A	A	G	C	1402
G	C	T	G	A	C	G	C	A	G	T	T	T	A	T	A	C	A	C	G	T	1502
G	T	T	G	A	C	G	C	A	G	T	T	T	A	T	A	C	A	C	G	T	1502
G	C	T	G	A	C	G	C	A	G	T	T	T	A	T	A	C	A	A	G	C	1502
T	C	C	G	A	A	G	C	A	G	T	T	T	A	T	A	C	A	C	G	T	1502
G	T	T	G	A	A	G	T	G	G	T	T	T	A	T	G	C	A	C	G	T	1505

With impute, *HLA-C*, Affy6.0

G	A	G	G	C	G	T	G	A	C	A	T	G	T	A	A	A	T	C	G	102
A	A	G	G	C	G	T	G	A	C	A	T	G	T	A	A	A	T	C	G	102
G	A	G	G	C	G	T	T	A	T	A	T	G	T	A	A	C	T	T	G	102
G	A	G	G	C	A	T	T	G	C	A	T	G	T	A	A	A	T	T	G	102
G	G	G	G	T	G	T	T	G	T	A	T	A	T	A	A	C	C	T	G	102
G	A	G	G	C	G	T	G	A	C	A	T	G	T	A	A	A	T	C	G	103
G	A	G	G	C	G	T	T	A	C	G	T	G	T	A	A	A	T	T	G	202
G	G	G	G	T	G	T	T	G	T	A	T	A	T	A	A	C	C	T	G	302
A	A	G	G	T	G	T	T	G	T	A	T	A	T	A	A	C	C	T	G	303
G	A	G	G	T	G	T	T	G	T	A	T	A	T	A	A	C	C	T	G	303
A	A	G	G	T	G	T	T	G	T	A	T	A	T	A	A	A	T	C	G	303
A	A	G	G	T	G	T	T	G	T	A	T	A	T	A	A	C	C	T	G	304
A	A	G	G	T	G	T	T	G	T	A	T	A	T	A	A	A	T	T	A	304
A	A	G	G	T	G	T	T	G	T	A	T	A	T	A	A	C	T	T	G	304

G	A	A	G	C	G	T	T	A	C	G	T	G	T	A	A	C	C	T	G	401
G	A	G	G	C	G	T	T	A	C	A	C	A	T	A	A	A	T	T	G	403
G	A	A	G	C	G	T	T	A	C	A	C	G	T	A	A	A	T	T	G	403
G	A	G	G	C	G	T	T	G	T	A	C	G	G	G	G	A	T	T	G	403
G	A	A	A	C	G	T	T	A	C	A	C	A	T	A	A	A	T	T	G	602
G	G	A	G	C	G	C	T	G	T	A	T	G	T	A	A	A	T	C	G	701
G	A	G	G	C	G	C	T	G	T	A	C	G	G	G	G	A	T	T	G	702
G	G	A	G	C	G	C	T	G	T	A	T	G	T	A	A	A	T	C	G	702
G	A	G	G	C	G	C	T	G	T	A	C	G	G	G	G	C	C	T	G	702
G	A	G	G	C	G	C	T	G	T	A	T	G	T	A	A	A	T	C	G	702
A	A	G	G	T	G	T	T	G	T	A	T	A	T	A	A	C	T	T	G	702
G	A	G	G	T	G	T	T	G	T	A	T	A	T	A	A	C	C	T	G	702
G	A	G	G	C	G	C	T	G	T	A	C	A	T	A	G	C	T	T	G	704
G	A	G	G	C	A	T	T	G	C	A	T	G	T	A	A	A	T	T	G	801
G	A	G	G	C	G	C	T	G	T	A	C	G	G	G	G	A	T	T	G	801
G	A	G	G	C	G	T	T	A	C	A	C	A	T	A	A	A	T	C	G	1202
G	A	G	G	C	G	T	T	A	C	A	C	A	T	A	A	C	C	T	G	1202
A	A	G	G	T	G	T	T	G	T	A	T	A	T	A	A	A	T	C	G	1202
G	A	A	A	C	G	T	T	A	C	A	C	G	T	A	A	A	T	T	G	1203
G	A	A	A	C	G	T	T	A	C	A	C	A	T	A	A	A	T	T	G	1203
G	A	A	G	C	G	T	T	A	C	A	T	G	T	A	A	C	C	T	G	1402
G	A	G	G	C	G	T	T	A	T	A	T	G	T	A	A	C	T	T	G	1502
G	A	G	G	C	G	T	T	A	T	A	T	G	T	A	A	A	T	C	G	1502

A	A	G	G	T	G	T	T	G	T	A	T	A	T	A	A	C	C	T	G	1505

With impute, *HLA-C*, Illumina550K

T	C	A	C	C	C	G	C	A	C	G	A	G	A	G	A	G	102
T	A	A	A	T	C	G	C	A	C	G	A	G	A	G	A	G	102
C	A	A	A	T	C	T	C	A	T	A	A	C	G	G	G	G	102
C	A	A	C	C	C	T	T	A	T	G	A	G	A	G	G	G	102
C	A	A	A	T	T	T	T	A	T	G	A	G	G	G	A	G	102
T	C	A	C	C	C	G	C	A	C	G	A	G	A	G	A	G	103
C	A	A	A	C	C	T	C	A	C	G	G	G	A	A	A	G	202
C	A	G	A	T	T	T	T	A	T	G	A	G	G	G	A	G	302
T	A	A	A	T	T	T	T	A	T	G	A	G	G	G	A	G	303
C	A	A	A	T	T	T	T	A	T	G	A	G	G	G	A	G	303
T	A	A	A	T	T	T	T	A	T	G	A	G	A	G	A	G	303
T	A	A	A	T	T	T	T	A	T	G	A	G	G	G	A	G	304
T	A	A	A	T	T	T	T	A	T	G	A	G	G	A	A	A	304
T	A	A	A	T	T	T	T	A	T	G	A	G	G	G	G	G	304
C	A	A	A	T	C	T	C	G	C	G	A	G	G	G	A	G	401
T	A	A	A	T	C	T	C	G	C	G	A	G	G	G	A	G	401
T	A	A	A	T	C	T	C	G	C	G	A	G	A	A	A	G	403
T	C	A	A	T	C	T	C	A	C	G	G	G	A	A	A	G	602
C	A	G	A	C	C	T	T	A	T	G	A	G	G	G	A	G	701
C	A	A	A	C	C	T	T	A	C	G	A	G	A	A	A	G	702

C	A	A	A	C	C	T	T	A	C	G	A	G	A	G	A	G	702
T	A	A	A	T	T	T	T	A	T	G	A	G	G	G	A	G	702
C	A	G	A	C	C	T	T	A	T	G	A	G	G	G	A	G	702
C	A	G	A	T	T	T	T	A	T	G	A	G	G	G	A	G	702
C	A	A	A	C	C	T	T	A	T	G	A	G	G	G	G	G	704
C	A	A	A	T	C	T	C	A	T	A	A	C	G	G	G	G	801
C	A	A	A	C	C	T	T	A	C	G	A	G	A	A	A	G	801
T	A	A	A	T	C	T	C	A	C	G	A	G	G	G	A	G	1202
T	A	A	A	T	T	T	T	A	T	G	A	G	A	G	G	G	1202
T	A	A	A	T	C	T	C	A	C	G	G	G	A	A	A	G	1203
T	C	A	C	C	C	T	C	A	C	G	G	G	A	A	A	G	1203
T	A	A	A	T	C	T	C	A	C	G	A	G	A	G	A	G	1402
C	A	A	C	C	C	T	T	A	T	G	A	G	A	G	G	G	1502
C	A	A	C	C	C	T	T	A	T	G	A	G	A	G	A	G	1502
T	C	A	C	C	C	T	T	A	T	G	A	G	A	G	G	G	1502
T	A	A	A	T	T	T	T	A	T	G	A	G	G	G	A	G	1505

With impute, *HLA-C*, Union

C	G	G	A	C	A	A	C	A	T	C	G	C	C	102
T	A	G	A	T	A	A	C	A	T	C	G	C	C	102
C	G	G	A	C	A	A	T	A	T	T	G	C	C	102
C	G	G	A	T	A	G	C	A	T	T	G	C	C	102
C	G	G	A	T	G	G	T	A	T	T	G	T	C	102

C	G	G	A	C	A	A	C	A	T	C	G	C	C	103
C	G	G	A	C	A	A	C	A	C	C	A	C	C	202
C	G	G	G	T	G	G	T	A	T	T	G	T	C	302
T	A	G	A	T	G	G	T	A	T	T	G	T	C	303
C	G	G	A	T	G	G	T	A	T	T	G	T	C	303
T	A	G	A	T	G	G	T	A	T	T	G	C	C	303
C	A	G	A	T	A	G	T	A	T	T	G	T	C	303
T	A	G	A	T	A	G	T	A	T	T	A	C	C	304
T	A	G	A	T	G	G	T	A	T	T	G	T	C	304
T	A	G	A	T	A	G	T	A	T	T	G	T	C	304
T	A	G	A	T	A	G	T	A	T	T	G	C	C	304
C	G	G	A	T	A	A	C	G	T	C	G	T	C	401
C	G	G	A	T	G	A	C	G	T	C	G	T	C	401
C	G	A	A	T	G	A	C	G	T	C	A	C	C	403
C	G	G	A	T	G	A	C	A	C	C	A	C	T	602
C	G	G	G	C	A	G	T	A	T	T	G	C	C	701
C	G	G	A	C	G	G	T	A	C	C	A	C	C	702
C	G	G	A	C	A	G	T	A	C	C	A	C	C	702
C	G	G	A	C	G	G	T	A	C	C	G	C	C	702
C	G	G	A	C	G	G	T	A	C	C	G	T	C	702
T	A	G	A	T	A	G	T	A	T	T	G	C	C	702
C	G	G	G	C	A	G	T	A	T	T	G	C	C	702
C	G	G	G	T	G	G	T	A	T	T	G	T	C	702

C	G	G	A	C	G	G	T	A	C	T	G	C	C	704
C	G	G	A	T	A	G	C	A	T	T	G	C	C	801
C	G	G	A	C	G	G	T	A	C	C	A	C	C	801
C	G	G	A	T	A	A	C	A	C	C	G	C	C	1202
C	G	G	A	T	A	A	C	A	C	C	G	T	C	1202
T	A	G	A	T	G	G	T	A	T	T	G	C	C	1202
C	G	A	A	T	A	A	C	A	C	C	A	C	T	1203
C	G	G	A	C	A	A	C	A	C	C	A	C	T	1203
C	G	G	A	T	A	A	C	A	T	C	G	T	C	1402
C	G	G	A	C	A	A	T	A	T	T	G	C	C	1502
T	G	G	A	C	A	A	T	A	T	T	G	C	C	1502
T	A	G	A	T	G	G	T	A	T	T	G	T	C	1505

With impute, *HLA-DPB1*, Affy5.0

T	T	A	A	C	T	T	G	C	A	T	G	A	A	A	G	G	C	T	T	A	C	G	T	G	C	G	C	G	T	C	A	A	G	101
T	T	G	A	C	T	C	G	C	A	T	G	A	A	A	G	G	C	T	T	A	C	G	T	G	C	G	C	A	C	C	C	G	G	102
T	T	G	A	C	T	C	G	C	A	C	A	G	G	C	A	A	T	C	G	G	T	A	C	A	T	T	T	G	C	C	C	G	G	102
C	C	A	A	T	T	T	A	T	A	C	A	G	G	C	A	A	T	C	G	G	T	A	C	A	T	T	T	G	C	C	C	G	G	201
T	T	G	A	C	T	C	G	T	A	C	A	G	G	C	A	A	T	C	G	G	T	A	C	A	T	T	T	G						

C C C G G 201

C T A A T T T G T A C A G G C A A T C G G T A C A T T T G

C C C G G 201

T T A A T T T A T A C A G G C A A T C G G T A C A T T T G

C C C G G 201

C C A A T T T A T A C A G G C A A T C G G T A C A T T T G

T C A A G 201

C T A A T T T G T A C A G G C A A T C G G T A C A T T T G

C C C G A 201

T T G A C T C A T A C A G G C A A T C G G T A C A T T T G

C C C G G 201

T T A A C T T G T A C A G G C A A T C G G T A C A T T T G

C C C G G 201

C T A A T T T A T G C A G G C A A T C G G T A C A T T T G

T C A A G 201

T T G A C T C G C A C A G G C A A T C G G T A C A T T T G

C C C G G 202

T T A A T T T A C A C A G G C A A T C G G T A C A T T T G

C C C G G 202

C C A A T T T A T A C A G G C A A T C G G T A C A T T T G

C C C G G 202

T T G A T T T A T A C A G G C A A T C G G T A C A T T T G

C C C G G 202

T T G A C T T G C A C A G G C A A T C G G T A C A T T T G

C C C G G 202

C T A A T T T G T A C A G G C A A T C G G T A C A T T T G

C C C G A 401

C T A A T T T G T A C A G G C A A T C G G T A C A T T T G

C C C G G 401

T T A A T T T A T A C A G G C A A T C G G T A C A T T T G

C C C G A 401

C T A A T T T A T A C A G G C A A T C G G T A C A T T T G

C C C G G 402

T T A G C C T G T A C A G G C A A T C G G T A C A T T T G

C C C G G 402

T T G A C T C G C A T G A A A G G C T T A C G T G C G C A

C C C G G 501

T T A A C T C G C A T G A A A G G C T T A C G T G C G C A

C T C G G 501

T T G A C T T G C A T G A A A G G C T T A C G T G C G C A

C C C G G 501

T T A A T T T A C A T G A A A G G C T T A C G T G C G C A

C C C G G 501

C C A A T T T A C A T G A A A G G C T T A C G T G C G C A

C C C G G 501

C T A A T T T G C A T G A A A G G C T T A C G T G C G C A

	C	C	C	G	G	501																						
T	T	A	G	C	C	C	A	C	A	T	G	A	A	A	G	G	C	T	T	A	C	G	T	G	C	G	C	A
	C	C	C	G	G	501																						
T	T	G	A	C	T	C	G	C	G	T	G	A	A	A	G	G	C	T	T	A	C	G	T	G	C	G	C	A
	T	C	A	A	G	501																						
T	T	A	A	T	T	T	A	T	A	C	A	A	A	A	G	G	C	T	T	A	C	G	T	G	C	G	C	A
	C	C	C	G	G	501																						
T	C	A	A	T	T	T	A	C	A	T	G	A	A	A	G	G	C	T	T	A	C	G	T	G	C	G	C	A
	C	C	C	G	G	501																						
T	T	G	A	C	T	C	G	C	G	T	G	A	A	A	G	G	C	T	T	A	C	G	T	G	C	G	C	A
	T	C	C	G	G	501																						
T	T	A	G	C	C	T	G	C	G	T	G	A	A	A	G	G	C	T	T	A	C	G	T	G	C	G	C	A
	T	C	C	A	G	901																						
T	T	G	A	C	T	T	G	C	A	T	G	A	A	A	G	G	C	T	T	A	C	G	T	G	C	G	C	A
	C	T	C	G	G	902																						
C	T	A	A	C	T	C	G	C	A	T	G	A	A	A	G	G	C	T	T	A	C	G	T	G	C	G	C	A
	C	T	C	G	G	902																						
T	T	G	A	C	T	C	G	C	A	T	G	A	A	A	G	G	C	T	T	A	C	G	T	G	C	G	C	A
	C	C	C	G	G	902																						
T	T	A	G	C	T	C	G	C	A	T	G	A	A	A	G	G	C	T	T	A	C	G	T	G	C	G	C	A
	C	T	C	G	G	902																						
T	T	G	A	C	T	C	A	C	A	T	G	A	A	A	G	G	C	T	T	A	C	G	T	G	C	G	C	A
	C	T	C	G	G	1301																						

T T A G C C C A C A T G A A A G G C T T A C G T G C G C A

C T C G G 1301

T T G A C T T G C A T G A A A G G C T T A C G T G C G C A

C T C G G 1301

T T A A C T C G C A T G A A A G G C T T A C G T G C G C A

C T C G G 1301

C T A A C T C G C A T G A A A G G C T T A C G T G C G C A

C T C G G 1301

T T G A C T C G C A T G A A A G G C T T A C G T G C G C A

C T C G G 1301

T T A G C C T G C G T G A A A G G C T T A C G T G C G C A

T C C G G 1401

T T A G C C T G C G T G A A A G G C T T A C G T G C G C A

T C C A G 1401

T T A A C T T G C A T G A A A G G C T T A C G T G C G C A

C T C G G 1401

T T A G C C T G C G T G A A A G G C T T A C G T G C G C A

T T C A G 1401

T T A G C C T G T A T A G G C A A T C G G T A C A T T T G

C C C G G 1701

T T A A T T T A T A C A A A A G G C T T A C G T G C G C A

C C C G G 1901

C T A A C T C G C A T G A A A G G C T T A C G T G C G C A

	C	T	C	G	G	1901																						
C	T	A	A	T	T	T	G	C	A	T	G	A	A	A	G	G	C	T	T	A	C	G	T	G	C	G	C	A
	T	C	A	A	G	2101																						
C	T	A	A	T	T	T	G	C	G	T	G	A	A	A	G	G	C	T	T	A	C	G	T	G	C	G	C	A
	T	C	A	A	G	2101																						
T	T	A	A	C	T	T	A	T	A	C	A	G	G	C	A	A	T	C	G	G	T	A	C	A	T	T	T	G
	C	C	C	G	G	3101																						
C	C	A	A	T	T	T	A	T	A	C	A	G	G	C	A	A	T	C	G	G	T	A	C	A	T	T	T	G
	C	C	C	G	G	4801																						

With impute, *HLA-DPB1*, Affy6.0

C	A	C	A	A	T	C	A	G	A	A	A	G	G	C	T	T	A	C	G	T	G	C	G	C	G	T	G	A
	G	A	A	G	101																							
T	G	C	G	G	T	C	A	G	A	A	A	G	G	C	T	T	A	C	G	T	G	C	G	C	A	C	G	G
	G	G	A	G	102																							
T	G	C	G	G	T	C	A	A	G	G	C	A	A	T	C	G	G	T	A	C	A	T	T	T	G	T	G	G
	G	A	A	G	102																							
C	A	C	A	A	T	T	A	A	G	G	C	A	A	T	C	G	G	T	A	C	A	T	T	T	G	T	G	G
	G	A	A	G	201																							
T	G	C	G	G	T	T	A	A	G	G	C	A	A	T	C	G	G	T	A	C	A	T	T	T	G	T	G	G
	G	A	A	G	201																							
C	A	C	A	A	T	T	A	A	G	G	C	A	A	T	C	G	G	T	A	C	A	T	T	T	G	T	G	G
	G	G	A	G	201																							

T G C G A T T A A G G C A A T C G G T A C A T T T G T G G

G A A G 201

C A C A A T T A A G G C A A T C G G T A C A T T T G T G G

G G A T 201

C A C A G T T A A G G C A A T C G G T A C A T T T G T G G

G A A G 201

T A C A G T T A A G G C A A T C G G T A C A T T T G T G G

A A A G 201

T G C G G T C A A G G C A A T C G G T A C A T T T G T G G

G A A G 202

T G C G A T T A A G G C A A T C G G T A C A T T T G T G G

G A A G 202

C A C A A T T A A G G C A A T C G G T A C A T T T G T G G

G A A T 202

T A C A G T T A A G G C A A T C G G T A C A T T T G T G G

A A A G 401

C A C A G T T A A G G C A A T C G G T A C A T T T G T G G

A A G G 401

T G C A A T T A A G G C A A T C G G T A C A T T T G T G G

A A A G 401

T A C A G T T A A G G C A A T C G G T A C A T T T G T G G

G A A G 401

C A T A A T T A A G G C A A T C G G T A C A T T T G T T G

G A A G 402

T G C G A T T A A G G C A A T C G G T A C A T T T G T T G

G A A G 402

C A T A G T T A A G G C A A T C G G T A C A T T T G T T G

G A A G 402

T G C G G T C A G A A A G G C T T A C G T G C G C A C G G

G G A G 501

T G C G G T C A G A A A G G C T T A C G T G C G C A C G G

G G A T 501

T G C G G T C A G A A A G G C T T A C G T G C G C A C G G

G A A G 501

T G C G G T C A G A A A G G C T T A C G T G C G C A C G A

G A A G 501

C A C A A T C A G A A A G G C T T A C G T G C G C A C G G

G G A G 501

T G C A A T T A A A A A G G C T T A C G T G C G C A C G G

G A A G 501

T A C A G T C A G A A A G G C T T A C G T G C G C A C G G

G G A G 501

C A C A A T C A G A A A G G C T T A C G T G C G C A C G G

G G A T 501

C A C A G A C G G A A A G G C T T A C G T G C G C A C G A

G A A G 901

T	G	C	G	G	A	C	A	G	A	A	A	G	G	C	T	T	A	C	G	T	G	C	G	C	A	C	G	G
	G	A	A	G	902																							
C	A	C	A	G	A	C	A	G	A	A	A	G	G	C	T	T	A	C	G	T	G	C	G	C	A	C	G	G
	G	A	A	G	902																							
T	G	C	G	G	T	C	A	G	A	A	A	G	G	C	T	T	A	C	G	T	G	C	G	C	A	C	G	G
	G	G	A	T	902																							
T	G	C	A	G	A	C	A	G	A	A	A	G	G	C	T	T	A	C	G	T	G	C	G	C	A	C	G	G
	G	A	A	G	902																							
T	G	C	A	G	A	C	A	G	A	A	A	G	G	C	T	T	A	C	G	T	G	C	G	C	A	C	G	G
	G	A	A	G	1301																							
C	A	C	A	A	A	C	A	G	A	A	A	G	G	C	T	T	A	C	G	T	G	C	G	C	A	C	G	G
	G	A	A	G	1301																							
T	G	C	G	G	A	C	A	G	A	A	A	G	G	C	T	T	A	C	G	T	G	C	G	C	A	C	G	G
	G	A	A	G	1301																							
T	G	C	G	A	A	C	A	G	A	A	A	G	G	C	T	T	A	C	G	T	G	C	G	C	A	C	G	G
	G	A	A	G	1301																							
C	A	C	A	A	T	C	A	G	A	A	A	G	G	C	T	T	A	C	G	T	G	C	G	C	A	C	G	G
	G	A	A	G	1301																							
C	A	C	A	G	A	C	G	G	A	A	A	G	G	C	T	T	A	C	G	T	G	C	G	C	A	C	G	G
	G	A	A	G	1401																							
C	A	C	A	G	A	C	G	G	A	A	A	G	G	C	T	T	A	C	G	T	G	C	G	C	A	C	G	A
	G	A	A	G	1401																							
C	A	C	A	G	T	T	A	A	G	G	C	A	A	T	C	G	G	T	A	C	A	T	T	T	G	T	T	G

G A A T 1701

T G C A A T T A A A A A G G C T T A C G T G C G C A C G G

G A A G 1901

T G C G G T C A G A A A G G C T T A C G T G C G C A C G G

G A A G 1901

C A C A G A C A G A A A G G C T T A C G T G C G C A C G A

G A A G 2101

T G C G G A C A G A A A G G C T T A C G T G C G C A C G A

G A A G 2101

T A C A G A C A G A A A G G C T T A C G T G C G C A C G A

G A A G 2101

C A C A G A C G G A A A G G C T T A C G T G C G C A C G A

G G A G 2101

T G C G A T T A A G G C A A T C G G T A C A T T T G T G G

G G A G 3101

C A C A A T T A A G G C A A T C G G T A C A T T T G T G G

G A A G 4801

With impute, *HLA-DPB1*, Illumina550K

G C C T A C G T A A A G G C A A T C G G T A C A T T T G C

C G G C A G T A 101

G T C T G C G C A G G A A A G G C T T A C G T G C G C A T

T G G C G A T G 102

G T C T G C G T A A A G G C A A T C G G T A C A T T T G C

C G T C G A C G 102

G C C C A T A T A A A G G C A A T C G G T A C A T T T G C

C G T C G A C G 201

A C C C A T A T A A A G G C A A T C G G T A C A T T T G C

C G T C G A C G 201

A T C T G C G T A A A G G C A A T C G G T A C A T T T G C

C G T C G A C G 201

G T C T G C A T A A A G G C A A T C G G T A C A T T T G C

C G T C G A C G 201

G T C T G C G C A A A G G C A A T C G G T A C A T T T G C

C G T C G A C G 201

A C C T A T G T A A A G G C A A T C G G T A C A T T T G C

C G T C G A C G 201

G T C T G T A T A A A G G C A A T C G G T A C A T T T G C

C G T C G A C G 201

A T C T G C G C A A A G G C A A T C G G T A C A T T T G C

C G T C G A C G 201

A T C T A T G T A A A G G C A A T C G G T A C A T T T G C

C G T C A A C G 201

G T C T G C G C A A A G G C A A T C G G T A C A T T T G C

C G T C G A C G 202

G T C T G C A T A A A G G C A A T C G G T A C A T T T G C

C G T C G A C G 202

A T C T G C G C A A A G G C A A T C G G T A C A T T T G C

C G T C G A C G 202

G T C T G C G T A A A G G C A A T C G G T A C A T T T G C

C G T C G A C G 202

G C C C A T A T A A A G G C A A T C G G T A C A T T T G C

C G T C G A C G 202

A T C T G C A C A A A G G C A A T C G G T A C A T T T G C

C G T C G A C G 202

A T C T A T G T A A A G G C A A T C G G T A C A T T T G C

C G T C A A C G 401

G T C T A T G T A A A G G C A A T C G G T A C A T T T G C

C G T C A A C G 401

G C C T A T G T A A A G G C A A T C G G T A C A T T T G C

C G T C A A C G 401

A C C T A T G T A A A G G C A A T C G G T A C A T T T G C

C G T C A A C G 401

G T C T A T A T A A A G G C A A T C G G T A C A T T T G C

C G T C A A C G 401

G C T T A T A T A A A G G C A A T C G G T A C A T T T G C

C T T C G A C G 402

A T C T G C G C A A A G G C A A T C G G T A C A T T T G C

C T T C G A C G 402

G T C T G C G C A G G A A A G G C T T A C G T G C G C A T

T G G C G A T G 501

A T C T G C G C A G G A A A G G C T T A C G T G C G C A T

T G G C G A T G 501

G T C T G C G C A G G A A A G G C T T A C G T G C G C G T

C G T T G A C G 501

G T C T G C G C A G G A A A G G C T T A C G T G C G C A T

T G G C A G T A 501

A T C T G C G C A G G A A A G G C T T A C G T G C G C G T

C G T T G A C G 501

A C C T A C G C A G G A A A G G C T T A C G T G C G C A T

T G G C G A T G 501

A C C C A T A T A G G A A A G G C T T A C G T G C G C A T

T G G C G A T G 501

A T C T G C G C G G G A A A G G C T T A C G T G C G C A T

T G G C A G T A 501

G T C T A T A T A A A A A A G G C T T A C G T G C G C A T

T G G C G A T G 501

G T C T G T A C A G G A A A G G C T T A C G T G C G C A T

T G G C G A T G 501

A T C T G C G C G G G A A A G G C T T A C G T G C G C A T

T G G C G A C G 501

A T C T A T G C A G G A A A G G C T T A C G T G C G C A T

	T	G	G	C	G	A	T	G	501																			
G	T	C	C	A	T	A	C	A	G	G	A	A	A	G	G	C	T	T	A	C	G	T	G	C	G	C	A	T
	T	G	G	C	G	A	T	G	501																			
A	C	C	T	A	C	G	C	G	G	G	A	A	A	G	G	C	T	T	A	C	G	T	G	C	G	C	A	T
	T	G	G	C	A	G	T	A	901																			
G	T	C	T	A	C	G	C	A	G	G	A	A	A	G	G	C	T	T	A	C	G	T	G	C	G	C	G	T
	C	G	T	T	G	A	C	G	902																			
G	T	C	T	G	C	G	C	A	G	G	A	A	A	G	G	C	T	T	A	C	G	T	G	C	G	C	A	T
	T	G	G	C	G	A	T	G	902																			
G	T	C	T	G	C	G	C	A	G	G	A	A	A	G	G	C	T	T	A	C	G	T	G	C	G	C	G	T
	C	G	T	T	G	A	C	G	902																			
G	T	C	T	G	C	G	C	A	G	G	A	A	A	G	G	C	T	T	A	C	G	T	G	C	G	C	G	T
	C	G	T	T	G	A	C	G	1301																			
G	T	C	T	G	C	A	C	A	G	G	A	A	A	G	G	C	T	T	A	C	G	T	G	C	G	C	G	T
	C	G	T	T	G	A	C	G	1301																			
G	C	C	T	A	C	A	C	A	G	G	A	A	A	G	G	C	T	T	A	C	G	T	G	C	G	C	G	T
	C	G	T	T	G	A	C	G	1301																			
G	T	C	T	A	C	G	C	A	G	G	A	A	A	G	G	C	T	T	A	C	G	T	G	C	G	C	G	T
	C	G	T	T	G	A	C	G	1301																			
A	C	C	T	A	C	A	C	A	G	G	A	A	A	G	G	C	T	T	A	C	G	T	G	C	G	C	G	T
	C	G	T	T	G	A	C	G	1301																			
G	C	C	T	A	C	G	C	G	G	G	A	A	A	G	G	C	T	T	A	C	G	T	G	C	G	C	A	T
	T	G	G	C	A	G	T	G	1401																			

G C C T A C G C G G G A A A G G C T T A C G T G C G C A T

T G G C A G T A 1401

A C C T A C G C G G G A A A G G C T T A C G T G C G C A T

T G G C A G T G 1401

A C C T A C G C G G G A A A G G C T T A C G T G C G C G T

C G T T G A C G 1401

G C C T A C G T A A A G G C A A T C G G T A C A T T T G C

C T T C G A C G 1701

G T C T A T A T A A A A A A G G C T T A C G T G C G C A T

T G G C G A T G 1901

A T C T G C G C A G G A A A G G C T T A C G T G C G C G T

C G T T G A C G 1901

G C C T A T G C A G G A A A G G C T T A C G T G C G C A T

T G G C A G T A 2101

G T C T G C A C A A A G G C A A T C G G T A C A T T T G C

C G T T G A C G 3101

G C C C A T A T A A A G G C A A T C G G T A C A T T T G C

C G T C G A C G 4801

With impute, *HLA-DPB1*, Union

G C T C T T C A T G G A A A G G C T T A C G T G C G C G G

C A G T A A A 101

G C T T C T C A T G G A A A G G C T T A C G T G C G C A G

	C	G	A	T	G	G	A	102																				
G	C	T	T	C	T	C	A	C	A	A	G	G	C	A	A	T	C	G	G	T	A	C	A	T	T	T	G	G
	C	G	A	C	G	A	A	102																				
G	C	C	T	T	T	T	A	C	A	A	G	G	C	A	A	T	C	G	G	T	A	C	A	T	T	T	G	G
	C	G	A	C	G	A	A	201																				
G	C	T	T	C	T	T	A	C	A	A	G	G	C	A	A	T	C	G	G	T	A	C	A	T	T	T	G	G
	C	G	A	C	G	A	A	201																				
G	C	C	T	T	T	T	A	C	A	A	G	G	C	A	A	T	C	G	G	T	A	C	A	T	T	T	G	G
	C	G	A	C	G	G	A	201																				
G	C	T	T	T	T	T	A	C	A	A	G	G	C	A	A	T	C	G	G	T	A	C	A	T	T	T	G	G
	C	G	A	C	G	A	A	201																				
G	C	T	T	T	T	T	A	C	A	A	G	G	C	A	A	T	C	G	G	T	A	C	A	T	T	T	G	G
	C	A	A	C	G	A	A	201																				
G	C	T	T	C	T	C	A	C	A	A	G	G	C	A	A	T	C	G	G	T	A	C	A	T	T	T	G	G
	C	G	A	C	G	A	A	202																				
G	C	T	T	T	T	C	A	C	A	A	G	G	C	A	A	T	C	G	G	T	A	C	A	T	T	T	G	G
	C	G	A	C	G	A	A	202																				
G	C	C	T	T	T	T	A	C	A	A	G	G	C	A	A	T	C	G	G	T	A	C	A	T	T	T	G	G
	C	G	A	C	G	A	A	202																				
G	C	T	T	T	T	T	A	C	A	A	G	G	C	A	A	T	C	G	G	T	A	C	A	T	T	T	G	G
	C	G	A	C	G	A	A	202																				
G	C	T	T	T	T	T	A	C	A	A	G	G	C	A	A	T	C	G	G	T	A	C	A	T	T	T	G	G
	C	A	A	C	G	A	A	401																				

A T T T T T T A C A A G G C A A T C G G T A C A T T T G T

T G A C G A A 402

G C T T T T T A C A A G G C A A T C G G T A C A T T T G T

T G A C G A A 402

G C T T C T C A T G G A A A G G C T T A C G T G C G C A G

C G A T G G A 501

G C T T T T C A T G G A A A G G C T T A C G T G C G C A G

C G A T G G A 501

G C T T C T C A T G G A A A G G C T T A C G T G C G C A G

C G A C G A A 501

G C T T C T C A T G G A A A G G C T T A C G T G C G C A G

C A G T A A A 501

G C C T T T C A T G G A A A G G C T T A C G T G C G C A G

C G A T G G A 501

G C T T T T T A C A A A A A G G C T T A C G T G C G C A G

C G A T G A A 501

G C T C T T C A T G G A A A G G C T T A C G T G C G C A G

C G A T G G A 501

G C T C T A C G T G G A A A G G C T T A C G T G C G C A G

C A G T A A G 901

G C T T T A C A T G G A A A G G C T T A C G T G C G C A G

C G A C G A A 902

G C T T C A C A T G G A A A G G C T T A C G T G C G C A G

	C	G	A	C	G	A	A	902																				
G	C	C	T	C	A	C	A	T	G	G	A	A	A	G	G	C	T	T	A	C	G	T	G	C	G	C	A	G
	C	G	A	C	G	A	A	902																				
G	C	T	T	C	T	C	A	T	G	G	A	A	A	G	G	C	T	T	A	C	G	T	G	C	G	C	A	G
	C	G	A	T	G	G	A	902																				
G	C	T	T	C	A	C	A	T	G	G	A	A	A	G	G	C	T	T	A	C	G	T	G	C	G	C	A	G
	C	G	A	C	G	A	A	1301																				
G	C	T	C	C	A	C	A	T	G	G	A	A	A	G	G	C	T	T	A	C	G	T	G	C	G	C	A	G
	C	G	A	C	G	A	A	1301																				
G	C	T	T	T	A	C	A	T	G	G	A	A	A	G	G	C	T	T	A	C	G	T	G	C	G	C	A	G
	C	G	A	C	G	A	A	1301																				
G	C	T	C	C	T	C	A	T	G	G	A	A	A	G	G	C	T	T	A	C	G	T	G	C	G	C	A	G
	C	G	A	C	G	A	A	1301																				
G	C	T	C	T	A	C	G	T	G	G	A	A	A	G	G	C	T	T	A	C	G	T	G	C	G	C	A	G
	C	A	G	T	G	A	G	1401																				
G	C	T	C	T	A	C	G	T	G	G	A	A	A	G	G	C	T	T	A	C	G	T	G	C	G	C	A	G
	C	A	G	T	A	A	G	1401																				
G	C	T	C	T	A	C	A	T	G	G	A	A	A	G	G	C	T	T	A	C	G	T	G	C	G	C	A	G
	C	G	A	C	G	A	A	1401																				
G	C	T	C	T	T	T	A	T	A	A	G	G	C	A	A	T	C	G	G	T	A	C	A	T	T	T	G	T
	C	G	A	C	G	A	A	1701																				
G	C	T	T	T	T	T	A	C	A	A	A	A	A	G	G	C	T	T	A	C	G	T	G	C	G	C	A	G
	C	G	A	T	G	A	A	1901																				

G C T T C T C A T G G A A A G G C T T A C G T G C G C A G
C G A C G A A 1901
G C T T T A C A T G G A A A G G C T T A C G T G C G C A G
C A G T A A A 2101
G C T T T T T A C A A G G C A A T C G G T A C A T T T G G
C G A C G G A 3101
G C C T T T T A C A A G G C A A T C G G T A C A T T T G G
C G A C G A A 4801

With impute, *HLA-DQB1*, Affy5.0

G	T	G	T	G	A	T	G	T	T	T	T	G	T	T	A	201
G	T	A	T	G	C	T	A	C	C	T	G	T	C	T	A	202
G	T	A	T	G	C	C	G	C	C	C	G	T	T	C	A	301
G	T	G	T	G	C	C	A	C	C	T	G	T	T	T	A	302
G	T	G	T	G	C	C	A	C	C	C	G	T	T	C	A	303
G	T	G	T	G	C	C	A	C	C	T	G	T	T	T	A	303
G	T	G	T	G	A	T	G	T	T	T	T	G	T	T	A	303
G	T	G	T	G	C	C	A	C	C	T	G	T	C	C	A	401
G	C	G	G	A	C	C	G	T	T	T	T	G	T	T	G	401
G	T	A	T	G	C	C	A	C	C	T	G	T	C	C	A	402
G	T	G	T	G	C	C	A	C	C	T	G	T	C	C	A	402
G	C	G	G	G	C	C	G	T	T	T	T	G	T	T	A	501
G	T	G	T	G	C	C	A	C	C	C	G	T	T	C	A	501

G	C	G	G	G	C	C	G	T	C	C	G	T	T	C	A	502
G	C	G	G	G	C	C	G	T	T	T	T	T	T	C	A	502
G	C	A	G	G	C	C	G	C	C	C	G	T	T	C	A	502
G	C	G	G	G	C	C	G	T	C	T	T	G	T	T	A	503
G	C	G	G	A	C	C	G	T	T	T	T	G	T	T	A	503
G	C	G	G	A	C	C	G	T	T	T	T	G	T	T	G	503
A	C	A	G	G	C	C	G	C	C	T	G	T	C	T	A	601
G	C	A	G	G	C	C	G	C	C	T	G	T	C	T	A	601
G	T	G	T	G	C	C	A	C	C	T	G	T	T	T	A	601
G	C	G	G	A	C	C	G	T	T	T	T	G	T	T	G	602
A	C	G	G	A	C	C	G	T	T	T	T	G	T	T	G	609
G	C	G	G	A	C	C	G	T	T	T	T	G	T	T	G	610

With impute, *HLA-DQB1*, Affy6.0

G	C	G	G	G	A	T	G	A	T	T	A	T	T	T	T	201
G	C	A	G	G	C	T	A	A	C	C	A	T	G	C	T	202
G	C	A	G	G	C	C	G	A	C	C	A	C	G	T	C	301
G	C	G	G	G	C	C	A	G	C	C	A	T	G	T	T	302
G	C	G	G	G	C	C	A	G	C	C	A	C	G	T	C	303
G	C	G	G	G	C	C	A	G	C	C	A	T	G	T	T	303
G	T	G	T	A	C	C	G	A	T	T	G	T	T	T	T	303
G	C	G	G	G	C	C	A	G	C	C	A	T	G	C	C	401
G	T	G	T	G	C	C	G	A	T	T	G	T	T	T	T	401

G	C	G	G	G	A	T	G	A	T	T	A	T	T	T	T	401
G	C	A	G	G	C	C	A	G	C	C	A	T	G	C	C	402
G	C	G	G	G	C	C	A	G	C	C	A	T	G	C	C	402
G	T	G	T	G	C	C	G	A	T	T	A	T	T	T	T	501
G	C	G	G	G	C	C	A	G	C	C	A	C	G	T	C	501
G	T	G	T	G	C	C	G	A	T	C	A	C	G	T	C	502
G	T	A	T	G	C	C	G	A	C	C	A	C	G	T	C	502
G	T	G	T	G	C	C	G	A	T	C	A	T	T	T	T	503
G	T	G	T	A	C	C	G	A	T	T	G	T	T	T	T	503
A	T	A	T	G	C	C	G	A	C	C	A	T	G	C	T	601
G	T	A	T	G	C	C	G	A	C	C	A	T	G	C	T	601
G	C	G	G	G	C	C	A	G	C	C	A	T	G	T	T	601
G	T	G	T	A	C	C	G	A	T	T	G	T	T	T	T	602
A	T	G	T	A	C	C	G	A	T	T	G	T	T	T	T	609
G	T	G	T	A	C	C	G	A	T	T	G	T	T	T	T	610

With impute, *HLA-DQB1*, Illumina550K

A	G	C	C	C	G	A	T	G	T	A	T	T	T	T	201
A	G	C	C	C	G	C	T	A	C	A	T	G	C	T	202
A	G	C	C	C	G	C	C	G	C	A	C	G	T	C	301
G	G	C	C	C	G	C	C	G	C	A	C	G	T	C	301
A	G	C	C	C	G	C	C	A	C	A	T	G	T	T	302
A	G	C	C	C	G	C	C	A	C	A	C	G	T	C	303

A	G	C	C	C	G	C	C	A	C	A	T	G	T	T	303
A	G	C	T	T	T	C	C	G	T	G	T	T	T	T	303
A	G	C	C	C	G	C	C	A	C	A	T	G	C	C	401
A	G	A	T	T	T	C	C	G	T	G	T	T	T	T	401
A	G	C	C	C	G	A	T	G	T	A	T	T	T	T	401
G	G	C	C	C	G	C	C	A	C	A	T	G	C	C	402
A	G	C	T	T	T	C	C	G	T	A	T	T	T	T	501
A	G	A	T	T	T	C	C	G	T	A	T	T	T	T	501
A	G	C	C	C	G	C	C	A	C	A	C	G	T	C	501
A	G	C	T	T	T	C	C	G	C	A	C	G	T	C	502
A	G	A	T	T	T	C	C	G	C	A	C	G	T	C	502
A	G	A	T	T	T	C	C	G	C	A	T	T	T	T	503
A	G	C	T	T	T	C	C	G	C	A	T	T	T	T	503
A	A	A	T	T	T	C	C	G	C	A	T	G	C	T	601
A	G	C	T	T	T	C	C	G	C	A	T	G	C	T	601
A	G	C	C	C	G	C	C	A	C	A	T	G	T	T	601
A	G	C	T	T	T	C	C	G	T	G	T	T	T	T	602
A	A	A	T	T	T	C	C	G	T	G	T	T	T	T	609
A	G	C	T	T	T	C	C	G	T	G	T	T	T	T	610

With impute, *HLA-DQB1*, Union

A	G	C	T	T	G	T	A	T	T	T	T	201
A	G	C	T	T	A	C	A	T	G	C	T	202

A	G	C	T	C	G	C	A	C	G	T	C	301
G	G	T	T	C	G	C	A	C	G	T	C	301
A	G	C	T	C	A	C	A	T	G	T	T	302
A	G	C	T	C	A	C	A	C	G	T	C	303
A	G	C	T	C	A	C	A	T	G	T	T	303
A	G	T	G	C	G	T	G	T	T	T	T	303
A	G	C	T	C	A	C	A	T	G	C	C	401
A	G	C	G	C	G	T	G	T	T	T	T	401
A	G	C	T	T	G	T	A	T	T	T	T	401
G	G	T	T	C	A	C	A	T	G	C	C	402
A	G	T	G	C	G	T	A	T	T	T	T	501
A	G	C	G	C	G	T	A	T	T	T	T	501
A	G	C	T	C	A	C	A	C	G	T	C	501
A	G	T	G	C	G	C	A	C	G	T	C	502
A	G	C	G	C	G	C	A	C	G	T	C	502
A	G	C	G	C	G	C	A	T	T	T	T	503
A	G	T	G	C	G	C	A	T	T	T	T	503
A	A	C	G	C	G	C	A	T	G	C	T	601
A	G	T	G	C	G	C	A	T	G	C	T	601
A	G	C	T	C	A	C	A	T	G	T	T	601
A	G	T	G	C	G	T	G	T	T	T	T	602
A	A	C	G	C	G	T	G	T	T	T	T	609
A	G	T	G	C	G	T	G	T	T	T	T	610

With impute, *HLA-DRB1*, Affy5.0

C	T	G	G	C	A	A	A	A	T	G	A	T	A	G	C	T	A	A	G	A	A	A	101
T	T	G	G	C	G	A	A	C	T	G	G	T	A	G	T	C	C	A	G	A	A	A	301
C	T	G	G	C	G	A	A	C	T	G	G	T	A	G	T	C	C	A	G	A	A	A	301
T	T	G	G	C	G	A	A	C	T	G	G	T	A	G	C	C	A	A	G	G	A	A	301
T	T	A	G	C	G	A	G	A	C	A	A	A	A	G	C	C	A	A	G	G	A	A	401
T	T	A	G	C	G	A	G	A	C	A	A	A	A	G	C	C	A	G	G	G	A	G	403
C	T	G	A	C	G	A	G	A	C	A	A	A	A	G	C	C	A	G	G	G	A	G	403
T	T	A	G	C	G	A	G	A	C	A	A	A	A	G	C	C	A	G	G	G	A	G	404
C	T	G	G	C	G	A	G	A	C	A	A	T	A	G	C	C	A	G	G	G	A	G	405
T	T	A	G	C	G	A	G	A	C	A	A	A	A	G	C	C	A	G	G	G	A	G	405
C	T	G	G	C	G	A	G	A	C	A	A	A	A	G	C	C	A	G	G	G	A	G	405
T	T	G	G	C	G	A	A	C	T	G	G	T	A	G	T	C	C	A	G	A	A	A	405
T	T	A	G	C	G	A	G	A	C	A	A	A	A	G	C	C	A	G	G	G	A	G	406
T	T	A	G	C	G	A	G	A	C	A	A	A	A	G	C	C	A	G	G	G	A	G	407
C	T	G	A	C	G	A	G	A	C	A	A	T	A	G	C	C	A	G	G	G	A	G	410
T	T	G	G	C	G	A	G	A	T	A	A	T	G	G	C	C	A	A	G	G	A	A	701
C	T	G	G	C	G	A	A	A	T	A	A	T	A	G	C	T	A	A	G	G	A	G	802
C	T	G	G	T	G	A	A	A	T	A	A	T	A	A	C	T	A	A	G	G	A	A	803
C	T	G	G	C	A	A	A	A	T	G	G	T	A	G	C	T	A	A	A	A	G	A	803
C	T	G	G	C	G	A	A	A	T	A	A	T	A	G	C	T	A	A	G	G	A	G	809
C	T	G	A	C	G	A	G	A	T	G	G	T	A	G	C	C	A	G	G	G	A	A	901

C	T	G	G	C	G	A	G	A	T	G	G	T	A	G	C	C	A	G	G	G	A	A	901
T	T	A	G	C	G	A	G	A	C	A	A	A	A	G	C	C	A	G	G	G	A	G	901
C	T	G	G	T	G	A	A	A	T	G	A	T	G	G	C	T	A	A	A	A	G	A	901
C	T	G	G	C	A	A	A	A	T	G	G	T	A	G	C	T	A	A	G	A	A	A	1001
C	T	G	A	C	G	A	G	A	T	G	G	T	A	G	C	C	A	G	G	G	A	A	1001
C	T	G	G	C	G	A	A	C	T	G	G	T	A	G	C	C	A	A	G	G	A	A	1101
C	T	G	G	C	G	G	A	C	T	G	G	T	A	G	C	C	A	A	G	G	A	A	1101
C	T	G	G	C	G	A	A	A	T	A	A	T	A	G	C	C	A	A	G	G	A	A	1106
C	T	G	G	T	G	G	A	C	T	G	G	T	A	G	C	C	A	A	G	G	A	A	1201
C	T	G	G	C	G	A	A	C	T	G	G	T	A	G	C	C	A	A	G	G	A	A	1201
C	C	G	G	T	G	G	A	C	T	G	G	T	A	G	C	C	A	A	G	G	A	A	1201
C	C	G	G	T	G	A	A	C	T	G	G	T	A	G	C	C	A	A	G	G	A	A	1201
C	T	G	G	T	G	A	A	C	T	G	G	T	A	G	T	C	A	A	G	G	A	A	1201
C	T	G	G	C	G	G	A	A	T	A	A	T	A	G	C	T	A	A	G	A	A	G	1202
C	T	G	G	C	G	G	A	A	T	A	A	T	A	G	C	T	A	A	G	G	A	A	1202
C	T	G	G	C	G	A	A	A	T	A	A	T	A	A	C	T	A	A	A	A	G	A	1301
C	T	G	G	C	G	A	A	A	T	A	A	T	A	A	C	T	A	A	A	A	G	A	1302
C	T	G	G	C	G	A	A	C	T	G	G	T	A	G	T	C	A	A	G	G	A	A	1312
C	T	G	G	C	G	A	A	C	T	G	G	T	A	G	C	C	A	A	G	G	A	A	1312
C	T	G	A	C	G	G	A	A	T	G	G	T	A	G	C	T	A	A	G	G	A	A	1401
C	T	G	G	C	G	G	A	A	T	G	G	T	A	G	C	T	A	A	G	G	A	A	1401
C	T	G	A	C	G	G	A	A	T	G	G	T	A	G	C	T	A	A	G	A	A	A	1401
C	T	G	G	C	G	G	A	A	T	G	G	T	A	G	C	T	A	A	G	A	A	A	1401

C	C	G	G	T	G	A	A	C	T	G	G	T	A	G	T	C	A	A	G	G	A	A	1403
C	T	G	G	C	G	G	A	A	T	G	G	T	A	G	C	T	A	A	G	G	A	A	1404
C	T	G	G	C	G	G	A	A	T	A	A	T	A	G	C	T	A	A	A	A	A	A	1404
C	T	G	G	C	G	G	A	A	T	G	G	T	A	G	C	T	A	A	G	A	A	A	1405
C	T	G	G	C	G	A	A	A	T	G	G	T	A	G	C	T	A	A	G	A	A	A	1405
T	T	G	G	C	G	G	A	A	T	G	G	T	A	G	C	T	A	A	G	A	A	A	1405
C	T	G	G	C	G	A	A	A	T	G	G	T	A	G	C	T	A	A	G	G	A	A	1410
C	T	G	G	C	G	G	A	A	T	G	G	T	A	G	C	T	A	A	G	A	A	A	1418
C	T	G	G	T	G	A	A	A	T	G	A	T	G	G	C	T	A	A	A	A	G	A	1501
C	T	G	G	T	G	A	A	A	T	G	A	T	G	G	C	T	A	A	G	G	A	A	1501
C	T	G	G	T	G	A	A	A	T	G	A	T	A	G	C	T	A	A	G	G	A	A	1501
C	T	G	G	C	G	A	A	A	T	G	A	T	G	G	C	T	A	A	G	A	A	A	1502
C	T	G	G	C	G	A	A	A	T	G	A	T	G	G	C	T	A	A	G	G	A	A	1502
C	T	G	G	T	G	A	A	A	T	G	A	T	A	G	C	T	A	A	G	G	A	A	1602
C	T	G	G	T	G	A	A	A	T	G	A	T	A	G	C	C	A	A	G	G	A	A	1602
C	T	G	G	C	G	A	A	A	T	G	A	T	A	G	C	T	A	A	G	G	A	A	1602

With impute, *HLA-DRB1*, Affy6.0

G	C	G	T	G	G	A	A	A	G	A	A	T	A	A	T	G	T	T	T	A	C	A	C	G	A	A	C	101
G	C	G	C	T	G	A	G	A	A	A	C	T	A	A	C	G	T	T	T	A	T	C	C	G	A	A	T	301
G	C	G	C	T	G	A	G	A	A	A	C	T	A	A	C	G	T	T	T	A	C	A	C	G	A	G	T	301
G	C	G	T	G	G	A	G	A	A	G	A	T	A	G	C	A	C	A	T	A	C	A	C	G	A	G	T	401
G	C	G	T	G	G	A	G	A	A	G	A	T	A	G	C	A	C	A	T	A	C	A	T	G	A	G	C	403

A	C	G	T	G	G	A	G	A	A	G	A	T	A	G	C	A	C	A	T	A	C	A	T	G	A	G	C	403
G	C	G	T	G	G	A	G	A	A	G	A	T	A	G	C	A	C	A	T	A	C	A	T	G	A	G	C	404
G	C	G	T	G	G	A	G	A	A	G	A	T	A	G	C	A	C	T	T	A	C	A	T	G	A	G	C	405
G	C	G	T	G	G	A	G	A	A	G	A	T	A	G	C	A	C	A	T	A	C	A	T	G	A	G	C	405
G	C	G	C	T	G	A	G	A	A	A	C	T	A	A	C	G	T	T	T	A	T	C	C	G	A	A	T	405
G	C	G	T	G	G	A	G	A	A	G	A	T	A	G	C	A	C	A	T	A	C	A	T	G	A	G	C	406
G	C	G	T	G	G	A	G	A	A	G	A	T	A	G	C	A	C	A	T	A	C	A	T	G	A	G	C	407
A	C	G	T	G	G	A	G	A	A	G	A	T	A	G	C	A	C	T	T	A	C	A	T	G	A	G	C	410
G	C	G	T	G	G	A	G	A	A	G	A	T	A	A	C	A	C	T	T	G	C	A	C	G	A	G	C	701
G	C	G	T	G	A	A	G	A	A	A	A	T	A	G	C	A	C	T	T	A	C	A	C	G	A	G	C	802
G	T	G	C	T	G	A	G	A	A	A	A	T	A	A	C	A	C	T	C	A	C	A	C	G	A	G	C	803
G	C	G	T	G	G	A	A	A	A	A	A	T	A	A	C	G	T	T	T	A	C	A	C	G	G	A	T	803
G	C	G	T	G	A	A	G	A	A	A	A	T	A	G	C	A	C	T	T	A	C	A	C	G	A	G	C	809
A	C	G	T	G	G	A	G	A	A	G	A	T	A	A	C	G	T	T	T	A	C	A	T	G	A	G	T	901
G	C	G	T	G	G	A	G	A	A	G	A	T	A	A	C	G	T	T	T	A	C	A	T	G	A	G	T	901
G	C	G	T	G	G	A	G	A	A	G	A	T	A	G	C	A	C	A	T	A	C	A	T	G	A	G	C	901
G	C	G	C	T	G	A	G	A	G	A	A	G	A	A	T	G	T	T	T	G	C	A	C	A	G	A	T	901
G	C	G	T	G	G	A	A	A	A	A	A	T	A	A	C	G	T	T	T	A	C	A	C	G	A	A	C	1001
A	C	G	T	G	G	A	G	A	A	G	A	T	A	A	C	G	T	T	T	A	C	A	T	G	A	G	T	1001
G	C	G	C	T	G	A	G	A	A	A	C	T	A	A	C	G	T	T	T	A	C	A	C	G	A	G	T	1101
G	C	G	T	G	A	A	G	A	A	A	C	T	A	A	C	G	T	T	T	A	C	A	C	G	A	G	T	1106
G	C	G	T	G	A	A	G	G	A	A	C	T	A	A	C	G	T	T	T	A	C	A	C	G	A	G	T	1201
G	T	G	T	G	A	A	G	G	A	A	C	T	A	A	C	G	T	T	T	A	C	A	C	G	A	G	T	1201

G	C	G	C	T	G	A	G	A	A	A	C	T	A	A	C	G	T	T	T	A	C	A	C	G	A	G	T	1201
A	T	G	C	G	A	A	G	A	A	A	C	T	A	A	C	G	T	T	T	A	T	A	C	G	A	G	T	1201
G	C	G	T	G	A	G	G	G	A	A	A	T	A	G	C	A	C	T	T	A	C	A	C	G	A	A	T	1202
G	C	G	T	G	A	G	G	G	A	A	A	T	A	G	C	A	C	T	T	A	C	A	C	G	A	G	T	1202
G	C	G	T	G	A	A	G	G	A	A	A	T	A	G	C	A	C	T	T	A	C	A	C	G	A	A	T	1202
G	C	A	T	G	A	G	G	A	A	A	A	T	G	A	C	A	C	T	C	A	C	A	C	A	G	A	T	1301
G	C	A	T	G	A	G	G	A	A	A	A	T	G	A	C	A	C	T	C	A	C	A	C	A	G	A	T	1302
G	C	G	C	T	G	A	G	A	A	A	C	T	A	A	C	G	T	T	T	A	T	A	C	G	A	G	T	1312
A	C	G	C	T	G	A	G	G	A	A	A	T	A	A	C	G	T	T	T	A	C	A	C	G	A	G	T	1401
G	C	G	C	T	G	A	G	G	A	A	A	T	A	A	C	G	T	T	T	A	C	A	C	G	A	G	T	1401
G	C	G	C	T	G	A	G	G	A	A	A	T	A	A	C	G	T	T	T	A	C	A	C	G	A	A	C	1401
A	C	G	C	T	G	A	G	G	A	A	A	T	A	A	C	G	T	T	T	A	C	A	C	G	A	A	C	1401
A	T	G	C	G	A	A	G	A	A	A	C	T	A	A	C	G	T	T	T	A	T	A	C	G	A	G	T	1403
G	C	G	C	T	G	A	G	G	A	A	A	T	A	A	C	G	T	T	T	A	C	A	C	G	A	G	C	1404
G	C	G	C	T	G	A	G	A	A	A	A	T	A	A	C	G	T	T	T	A	C	A	C	G	A	A	C	1404
G	C	G	C	T	G	A	G	G	A	A	A	T	A	A	C	G	T	T	T	A	C	A	C	G	A	A	C	1405
G	C	G	C	T	G	A	G	A	A	A	A	T	A	A	C	G	T	T	T	A	C	A	C	G	A	A	C	1405
G	C	G	C	T	G	A	G	A	A	A	A	T	A	A	C	G	T	T	T	A	C	A	C	G	A	G	C	1405
G	C	G	T	G	A	A	G	G	A	A	A	T	A	A	C	G	T	T	T	A	C	A	C	G	A	G	C	1410
G	C	G	C	T	G	A	G	G	A	A	A	T	A	A	C	G	T	T	T	A	C	A	C	G	A	A	C	1418
G	C	G	C	T	G	A	G	A	G	A	A	G	A	A	T	G	T	T	T	G	C	A	C	A	G	A	T	1501
G	C	G	C	T	G	A	G	A	G	A	A	G	A	A	T	G	T	T	T	G	C	A	C	G	A	G	C	1501
G	C	G	C	T	G	A	G	A	G	A	A	G	A	A	T	G	T	T	T	A	C	A	C	G	A	G	T	1501

G	C	G	C	T	G	A	G	A	G	A	A	G	A	A	T	G	T	T	T	G	C	A	C	G	A	G	T	1501
G	C	G	T	G	A	A	G	A	G	A	A	G	A	A	T	G	T	T	T	G	C	A	C	G	A	A	C	1502
G	C	G	T	G	A	A	G	A	G	A	A	G	A	A	T	G	T	T	T	G	C	A	C	G	A	G	T	1502
G	C	G	T	G	A	A	G	A	G	A	A	G	A	A	T	G	T	T	T	G	C	A	C	G	A	G	C	1502
G	C	G	C	T	G	A	G	A	G	A	A	G	A	A	T	G	T	T	T	A	C	A	C	G	A	G	T	1602

With impute, *HLA-DRB1*, Illumina550K

A	C	G	C	C	G	G	T	A	A	A	G	A	A	G	T	A	C	A	C	A	A	C	C	C	101
A	T	G	C	T	T	G	C	G	G	A	A	C	A	G	T	A	T	C	C	A	A	C	C	C	301
G	T	G	C	T	T	G	C	G	G	A	A	C	A	G	T	A	C	A	C	A	A	A	C	C	301
A	C	G	C	T	T	G	C	G	G	A	A	C	A	G	T	A	T	C	C	A	A	C	C	C	301
G	T	G	C	T	G	G	C	A	G	A	A	A	A	A	A	A	C	A	C	A	A	A	C	C	401
G	T	G	C	T	G	G	C	A	G	A	A	A	A	A	A	A	C	A	T	A	G	A	C	C	403
A	C	A	C	T	G	G	C	A	G	A	A	A	A	A	A	A	C	A	T	G	G	C	C	C	403
G	T	G	C	T	G	G	C	A	G	A	A	A	A	A	A	A	C	A	T	A	G	A	C	C	404
A	C	G	C	T	G	G	C	A	G	A	A	A	A	A	T	A	C	A	T	A	G	C	C	C	405
A	C	G	C	T	G	G	C	A	G	A	A	A	A	A	T	A	C	A	T	A	G	A	C	C	405
A	T	G	C	T	T	G	C	G	G	A	A	C	A	G	T	A	T	C	C	A	A	C	C	C	405
G	T	G	C	T	G	G	C	A	G	A	A	A	A	A	A	A	C	A	T	A	A	A	C	C	405
A	C	G	C	C	G	G	T	A	A	A	A	A	A	G	T	A	C	A	C	G	G	A	C	C	405
G	T	G	C	T	G	G	C	A	G	A	A	A	A	A	A	A	C	A	T	A	G	A	C	C	406
G	T	G	C	T	G	G	C	A	G	A	A	A	A	A	A	A	C	A	T	A	G	A	C	C	407
A	C	A	C	T	G	G	C	A	G	A	A	A	A	A	T	A	C	A	T	A	G	A	C	C	410

A	T	G	C	T	G	G	C	A	G	A	A	A	A	A	T	G	C	A	C	A	A	A	C	C	701
A	C	G	C	T	G	G	C	A	G	A	A	A	A	A	T	G	C	A	C	A	A	A	C	C	701
A	C	G	C	T	G	A	C	G	G	A	A	A	A	A	T	A	C	A	C	A	G	A	C	C	802
A	C	G	T	C	T	G	C	G	G	A	A	A	A	A	T	A	C	A	C	A	A	A	C	C	803
G	T	G	C	T	G	G	C	A	G	A	A	A	A	A	A	A	C	A	T	A	A	A	C	C	803
A	C	G	C	C	G	G	T	A	A	A	A	A	A	G	T	A	C	A	C	G	G	A	C	C	803
A	C	G	C	T	G	A	C	G	G	A	A	A	A	A	T	A	C	A	C	A	G	A	C	C	809
A	C	A	C	T	G	G	C	A	G	A	A	A	A	G	T	A	C	A	T	A	A	A	C	C	901
A	C	G	C	T	G	G	C	A	G	A	A	A	A	G	T	A	C	A	T	A	A	A	C	C	901
G	T	G	C	T	G	G	C	A	G	A	A	A	A	A	A	A	C	A	T	A	G	A	C	C	901
A	C	G	T	C	T	G	C	G	G	A	G	A	A	G	T	G	C	A	C	G	A	C	C	C	901
A	C	G	C	C	G	G	T	A	A	A	A	A	A	G	T	A	C	A	C	A	A	A	C	T	1001
A	C	A	C	T	G	G	C	A	G	A	A	A	A	G	T	A	C	A	T	A	A	A	C	C	1001
A	C	G	C	T	T	G	C	G	G	A	A	C	A	G	T	A	C	A	C	A	A	A	C	C	1101
A	C	G	C	C	G	A	C	G	G	A	A	C	A	G	T	A	C	A	C	A	A	A	C	C	1106
A	C	G	T	C	G	A	C	G	G	G	A	C	A	G	T	A	C	A	C	A	A	A	C	C	1201
A	C	G	C	T	T	G	C	G	G	A	A	C	A	G	T	A	C	A	C	A	A	A	C	C	1201
A	C	G	C	C	G	A	C	G	G	A	A	C	A	G	T	A	C	A	C	A	A	A	C	C	1201
A	C	G	T	C	G	A	C	G	G	A	A	C	A	G	T	A	C	A	C	A	A	A	C	C	1201
A	C	G	T	C	G	A	C	G	G	G	A	C	A	G	T	A	T	A	C	A	A	A	C	C	1201
A	C	G	C	C	G	A	C	G	G	G	A	A	A	A	T	A	C	A	C	A	G	A	C	C	1202
A	C	G	C	C	G	A	C	G	G	G	A	A	A	A	T	A	C	A	C	A	A	A	C	C	1202
A	C	G	C	T	G	A	C	G	G	G	A	A	A	A	T	A	C	A	C	A	G	A	C	C	1202

A	C	G	C	T	G	A	C	G	G	G	A	A	A	A	T	A	C	A	C	A	G	C	C	C	1202
A	C	G	C	C	G	A	C	G	G	A	A	A	G	A	T	A	C	A	C	G	A	C	C	C	1301
A	C	G	C	C	G	A	C	G	G	A	A	A	G	A	T	A	C	A	C	G	A	C	T	C	1302
A	C	G	C	T	T	G	C	G	G	A	A	C	A	G	T	A	T	A	C	A	A	A	C	C	1312
A	C	G	C	T	T	G	C	G	G	G	A	A	A	G	T	A	C	A	C	A	A	A	C	C	1401
A	C	A	C	T	T	G	C	G	G	G	A	A	A	G	T	A	C	A	C	A	A	A	C	C	1401
A	C	G	T	C	G	A	C	G	G	A	A	C	A	G	T	A	T	A	C	A	A	A	C	C	1403
A	C	G	C	T	T	G	C	G	G	G	A	A	A	G	T	A	C	A	C	A	A	A	C	C	1404
A	C	G	C	T	T	G	C	G	G	G	A	A	A	G	T	A	C	A	C	A	A	C	C	C	1405
A	C	G	C	T	T	G	C	G	G	A	A	A	A	G	T	A	C	A	C	A	A	C	C	C	1405
A	C	G	C	T	T	G	C	G	G	G	A	A	A	G	T	A	C	A	C	A	A	A	C	C	1405
G	T	G	C	T	T	G	C	G	G	G	A	A	A	G	T	A	C	A	C	A	A	C	C	C	1405
A	C	G	C	C	G	A	C	G	G	A	A	A	A	G	T	A	C	A	C	A	A	C	C	C	1410
A	C	G	C	T	T	G	C	G	G	G	A	A	A	G	T	A	C	A	C	A	A	C	C	C	1418
A	C	G	T	C	T	G	C	G	G	A	G	A	A	G	T	G	C	A	C	A	A	A	C	C	1501
A	C	G	T	C	T	G	C	G	G	A	G	A	A	G	T	G	C	A	C	G	A	C	C	C	1501
A	C	G	C	C	G	A	C	G	G	A	G	A	A	G	T	G	C	A	C	A	A	A	C	C	1502
A	C	G	T	C	T	G	C	G	G	A	G	A	A	G	T	A	C	A	C	A	A	A	C	C	1602
A	C	G	C	T	T	G	C	G	G	A	G	A	A	G	T	A	C	A	C	A	A	A	C	C	1602

With impute, *HLA-DRB1*, Union

G	G	C	A	A	C	A	G	A	T	G	A	G	T	A	C	A	C	A	T	C	101
G	T	C	A	A	C	A	G	C	T	C	A	G	T	A	T	C	C	A	T	C	301

G	T	C	A	A	C	A	G	C	T	C	A	G	T	A	C	A	C	A	G	C	301
G	G	T	A	A	C	C	A	A	C	G	G	A	A	A	C	A	C	A	G	C	401
G	G	T	A	A	C	C	A	A	C	G	G	A	A	A	C	A	T	A	G	C	403
A	G	T	A	A	C	C	A	A	C	G	G	A	A	A	C	A	T	A	G	C	403
G	G	T	A	A	C	C	A	A	C	G	G	A	A	A	C	A	T	A	G	C	404
G	G	T	A	A	C	C	A	A	C	G	G	A	T	A	C	A	T	A	G	C	405
G	G	T	A	A	C	C	A	A	C	G	G	A	A	A	C	A	T	A	G	C	405
G	T	C	A	A	C	A	G	C	T	C	A	G	T	A	T	C	C	A	T	C	405
G	G	T	A	A	C	C	A	A	C	G	G	A	A	A	C	A	T	A	G	C	406
G	G	T	A	A	C	C	A	A	C	G	G	A	A	A	C	A	T	A	G	C	407
A	G	T	A	A	C	C	A	A	C	G	G	A	T	A	C	A	T	A	G	C	410
G	G	T	A	A	C	C	A	A	T	G	A	A	T	G	C	A	C	A	G	C	701
G	G	C	A	A	C	A	G	A	T	C	G	A	T	A	C	A	C	A	G	C	802
G	T	C	A	A	C	A	G	A	T	C	A	A	T	A	C	A	C	A	G	C	803
G	G	C	A	A	C	A	G	A	T	C	A	G	T	A	C	A	C	G	T	C	803
G	T	C	A	A	C	A	G	A	T	C	A	A	T	A	C	A	C	A	G	T	803
G	G	C	A	A	C	A	G	A	T	C	G	A	T	A	C	A	C	A	G	C	809
A	G	T	A	A	C	C	A	A	T	C	A	G	T	A	C	A	T	A	G	C	901
G	G	T	A	A	C	C	A	A	T	C	A	G	T	A	C	A	T	A	G	C	901
G	G	T	A	A	C	C	A	A	C	G	G	A	A	A	C	A	T	A	G	C	901
G	T	C	A	A	T	A	G	A	T	G	A	G	T	G	C	A	C	G	T	C	901
G	G	C	A	A	C	A	G	A	T	C	A	G	T	A	C	A	C	A	T	C	1001
A	G	T	A	A	C	C	A	A	T	C	A	G	T	A	C	A	T	A	G	C	1001

G	T	C	A	A	C	A	G	C	T	C	A	G	T	A	C	A	C	A	G	C	1101
G	G	C	A	A	C	A	G	C	T	C	A	G	T	A	C	A	C	A	G	C	1106
G	G	C	A	G	C	A	G	C	T	C	A	G	T	A	C	A	C	A	G	C	1201
G	T	C	A	A	C	A	G	C	T	C	A	G	T	A	C	A	C	A	G	C	1201
G	G	C	A	G	C	A	G	C	T	C	A	G	T	A	T	A	C	A	G	C	1201
G	G	C	G	G	C	A	G	A	T	C	G	A	T	A	C	A	C	A	G	C	1202
G	G	C	A	G	C	A	G	A	T	C	G	A	T	A	C	A	C	A	G	C	1202
G	G	C	G	A	C	A	G	A	T	C	A	A	T	A	C	A	C	G	T	C	1301
G	G	C	G	A	C	A	G	A	T	C	A	A	T	A	C	A	C	G	T	T	1302
G	T	C	A	A	C	A	G	C	T	C	A	G	T	A	T	A	C	A	G	C	1312
A	T	C	A	G	C	A	G	A	T	C	A	G	T	A	C	A	C	A	G	C	1401
G	T	C	A	G	C	A	G	A	T	C	A	G	T	A	C	A	C	A	G	C	1401
G	T	C	A	G	C	A	G	A	T	C	A	G	T	A	C	A	C	A	T	C	1401
A	T	C	A	G	C	A	G	A	T	C	A	G	T	A	C	A	C	A	T	C	1401
G	G	C	A	A	C	A	G	C	T	C	A	G	T	A	T	A	C	A	G	C	1403
G	T	C	A	G	C	A	G	A	T	C	A	G	T	A	C	A	C	A	T	C	1404
G	T	C	A	A	C	A	G	A	T	C	A	G	T	A	C	A	C	A	T	C	1404
G	T	C	A	G	C	A	G	A	T	C	A	G	T	A	C	A	C	A	T	C	1405
G	T	C	A	A	C	A	G	A	T	C	A	G	T	A	C	A	C	A	T	C	1405
G	G	C	A	A	C	A	G	A	T	C	A	G	T	A	C	A	C	A	T	C	1410
G	T	C	A	G	C	A	G	A	T	C	A	G	T	A	C	A	C	A	T	C	1418
G	T	C	A	A	T	A	G	A	T	G	A	G	T	G	C	A	C	G	T	C	1501
G	T	C	A	A	T	A	G	A	T	G	A	G	T	G	C	A	C	A	G	C	1501

G	T	C	A	A	C	A	G	A	T	G	A	G	T	G	C	A	C	A	G	C	1501
G	T	C	A	A	C	A	G	A	T	G	A	G	T	G	C	A	C	G	T	C	1501
G	T	C	A	A	T	A	G	A	T	G	A	G	T	G	C	A	C	A	T	C	1501
G	G	C	A	A	T	A	G	A	T	G	A	G	T	G	C	A	C	A	T	C	1502
G	G	C	A	A	T	A	G	A	T	G	A	G	T	G	C	A	C	A	G	C	1502
G	T	C	A	A	T	A	G	A	T	G	A	G	T	A	C	A	C	A	G	C	1602
